# An AlFe_2_O_4_@SiO_2_–SO_3_H innovative nanocatalyst: a sustainable approach for the A3 coupling reaction in a DES for 2-thioarylbenzoazoles†

**DOI:** 10.1039/d5na00247h

**Published:** 2025-05-08

**Authors:** Ahmad Sajjadi, Suranjana V. Mayani, Suhas Ballal, Shaker Al-Hasnaawei, Abhayveer Singh, Kattela Chennakesavulu, Kamal Kant Joshi

**Affiliations:** a Young Researchers and Elite Club, Tehran Branch, Islamic Azad University Tehran Iran sajjadiahmmad@gmail.com; b Marwadi University Research Center, Department of Chemistry, Faculty of Science, Marwadi University Rajkot Gujarat India suranjana.mayani@marwadieducation.edu.in; c Department of Chemistry and Biochemistry, School of Sciences, JAIN (Deemed to be University) Bangalore Karnataka India b.suhas@jainuniversity.ac.in; d College of Pharmacy, The Islamic University Najaf Iraq; e Department of Medical Analysis, Medical Laboratory Technique College, The Islamic University of Al Diwaniyah Al Diwaniyah Iraq shakeralhasnawi@iunajaf.edu.iq; f Centre for Research Impact & Outcome, Chitkara University Institute of Engineering and Technology, Chitkara University Rajpura 140401 Punjab India abhayveer_singh@outlook.com; g Department of Chemistry, Sathyabama Institute of Science and Technology Chennai Tamil Nadu India chennakesavulureddy.chemistry@sathyabama.ac.in; h Department of Allied Science, Graphic Era Hill University Dehradun India; i Graphic Era Deemed to be University Dehradun Uttarakhand India kkjoshi@gehu.ac.in

## Abstract

The A3 coupling reaction, which facilitates the synthesis of 2-thioarylbenzoazoles, represents a significant transformation in organic chemistry with implications in drug discovery and materials science. In this study, we introduce a novel nanocatalyst, AlFe_2_O_4_@SiO_2_–SO_3_H, designed to enhance the efficiency and sustainability of this reaction. The catalyst is synthesized by functionalizing aluminum ferrite (AlFe_2_O_4_) with chlorosulfonic acid (Cl–SO_3_H), promoting its role as an effective acid catalyst. Utilizing deep eutectic solvents (DESs) as a green reaction medium further contributes to the sustainability of the process by providing a biodegradable and non-toxic environment. Optimization studies were conducted to determine the optimal reaction conditions, including catalyst loading, temperature, and solvent composition. Characterization of the nanocatalyst was performed using techniques such as X-ray diffraction (XRD), scanning electron microscopy (SEM), and Fourier-transform infrared spectroscopy (FTIR) to confirm successful synthesis and functionalization. The AlFe_2_O_4_@SiO_2_–SO_3_H nanocatalyst exhibited excellent catalytic activity, resulting in high yields of 2-thioarylbenzoazoles under mild conditions while allowing for easy recovery and reuse without significant loss of activity. This work demonstrates the potential of AlFe_2_O_4_@SiO_2_–SO_3_H as a sustainable catalytic system for A3 coupling reactions, contributing to the development of environmentally friendly methodologies in organic synthesis.

## Abstract 2

The study presents a new nanocatalyst, AlFe_2_O_4_@SiO_2_-SO_3_H, designed to improve the efficiency and sustainability of the A3 coupling reaction, which is important for synthesizing 2-thioarylbenzoazoles.

## Graphical abstract

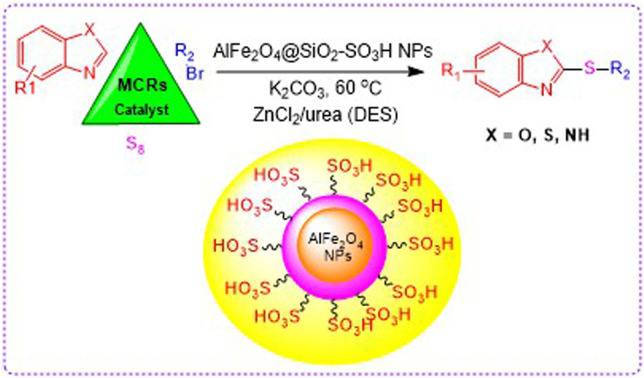

## 1 Introduction

In the realm of modern chemistry, the pursuit of sustainable and environmentally friendly methodologies has become paramount. The increasing global emphasis on green chemistry has driven researchers to explore innovative approaches that minimize environmental impact while maximizing efficiency in chemical processes. One area of particular interest is the development of novel catalysts that can facilitate complex organic syntheses under mild conditions, with reduced waste generation and improved atom economy. Catalysis, a critical process in manufacturing fine chemicals and pharmaceuticals, is fundamental in enhancing reaction rates, selectivity, and product yield while mitigating environmental impact. Within this context, the transition towards greener methodologies has driven the search for novel catalysts that are effective, environmentally benign, and cost-efficient. Due to the unique properties arising from their high surface area to volume ratio, nanocatalysts have emerged as promising candidates in this domain.^1–5^

The core of the nanocatalyst consists of AlFe_2_O_4_, a mixed metal oxide with a spinel structure, which has garnered considerable attention owing to its magnetic properties, chemical stability, and potential for functionalization. This composition was chosen for its stability, magnetic properties, and potential synergistic effects between aluminum and iron in catalysis. The surface of the AlFe_2_O_4_ nanoparticles is functionalized with chlorosulfonic acid, which provides strong acidic sites necessary for catalyzing the desired organic transformations. Incorporating sulfonic acid groups (–SO_3_H) into these ferrites yields hybrid nanocatalysts that exhibit Lewis and Brønsted acid catalytic features.^6–10^

These characteristics are particularly advantageous in promoting various organic transformations, including A3 coupling, a well-established method for synthesizing valuable heterocyclic compounds. Furthermore, introducing a phenyl moiety enhances the dispersion of the catalyst in organic solvents, thereby increasing its efficacy in catalyzing reactions of diverse substrates. The A3 coupling reaction, involving the condensation of an aldehyde, an amine, and a terminal alkyne, has become an invaluable strategy in organic synthesis, allowing for the straightforward formation of multidimensional structures.^11–15^

The resultant products, such as 2-thioaryl-benzothiazoles, 2-thioaryl-benzoxazoles, and 2-thioaryl-benzimidazoles, have significant applications in medicinal chemistry, exhibiting a wide range of biological activities, including antimicrobial, anticancer, and anti-inflammatory properties. However, toxic solvents and lengthy reaction times can often hinder traditional synthesis routes, necessitating the development of more sustainable methods.^16–25^

Deep eutectic solvents (DESs), particularly those based on simple and benign components, offer a green alternative to conventional organic solvents. Using a ZnCl_2_/urea system as a DES facilitates the solubility of reactants, enhances reaction rates, and promotes selective transformations under mild conditions. This approach alleviates the environmental concerns associated with traditional organic solvents and simplifies product recovery through ease of phase separation or extraction. The synergy between the AlFe_2_O_4_@SiO_2_–SO_3_H nanocatalyst and DES contributes to an overall reduction in the ecological footprint of the synthetic process. Using a nanocatalyst with a deep eutectic solvent represents a synergistic approach to green chemistry. The nanocatalyst provides a high surface area and recyclability, while the DES offers a non-volatile, biodegradable reaction medium. This combination simultaneously addresses several principles of green chemistry, including waste prevention, atom economy, and safer solvents and auxiliaries.^26–30^

Recently, the concept of one-pot synthesis has gained traction as a powerful strategy to streamline chemical processes. This approach allows multiple reactions to occur in a single vessel, eliminating the need for intermediate purification steps and significantly reducing solvent waste. When combined with green solvents and efficient catalysts, one-pot syntheses represent a promising direction for sustainable chemistry. The implications of this research extend beyond the specific reactions studied, as the AlFe_2_O_4_@SiO_2_–SO_3_H nanocatalyst exemplifies a versatile platform that can be adapted for a myriad of transformations in organic chemistry. Its efficient design underscores a broader initiative towards developing catalysts that align with the principles of green chemistry, emphasizing minimal environmental impact and maximal efficiency.^31–35^

The research presented in this manuscript introduces a groundbreaking advancement in organic chemistry by developing a novel nanocatalyst, AlFe_2_O_4_@SiO_2_–SO_3_H. This innovative catalyst effectively facilitates the A3 coupling reaction, a key process for synthesizing 2-thioarylbenzoazoles. These compounds are highly desired in drug discovery and materials science, making the catalyst's role particularly crucial.

### Key innovations

(1) Novel nanocatalyst design: the synthesis of AlFe_2_O_4_@SiO_2_–SO_3_H represents a pioneering approach in the field. By functionalizing aluminum ferrite with sulfonic acid groups, we have created a catalyst that enhances its role as an acid catalyst, thereby improving catalytic efficiency in the A3 coupling reaction. This unique design is sure to pique your interest.

(2) Sustainability focus: using deep eutectic solvents (DESs) as a reaction medium significantly shifts toward greener methodologies. A biodegradable and non-toxic DES provides an environmentally friendly alternative to traditional solvents in organic synthesis, aligning with the current trend of sustainable practices in the field.

(3) Optimization and characterization: comprehensive optimization studies involving systematic catalyst loading and temperature variation reveal precise conditions for maximizing the reaction yield. Techniques such as XRD, SEM, and FTIR confirm the catalyst's successful synthesis and functionalization, bolstering the results' reliability.

(4) High yields and reusability: the catalyst's exceptional performance under mild reaction conditions, achieving high yields of the desired products while maintaining robustness through multiple reaction cycles, underscores its potential for practical applications in industrial settings. This is particularly promising in contexts where sustainability and efficiency are paramount, and it gives us reason to be optimistic about its future applications.

In the present study, we elucidate the synthesis of a highly efficient and reusable AlFe_2_O_4_@SiO_2_–SO_3_H nanocatalyst, designed explicitly for the one-pot green synthesis of 2-thioaryl-benzothiazoles, 2-thioaryl-benzoxazoles, and 2-thioaryl-benzimidazoles *via* A3 coupling reactions. The unique structural properties of the AlFe_2_O_4_ matrix combined with the functionalities provided by the phenyl and sulfonic acid groups are expected to enhance catalytic activity while maintaining robustness during cyclical reactions. By employing a deep eutectic solvent system based on ZnCl_2_/urea, we aim to optimize reaction conditions for high selectivity and yield, affirming our commitment to advancing sustainable practices in organic synthesis. By demonstrating the efficacy of the AlFe_2_O_4_@SiO_2_–SO_3_H nanocatalyst in facilitating complex heterocycle syntheses under mild conditions, we hope to inspire further research into the development of innovative catalytic systems that can address the pressing need for more environmentally benign chemical processes.

## Results and discussion

**Scheme 1:**
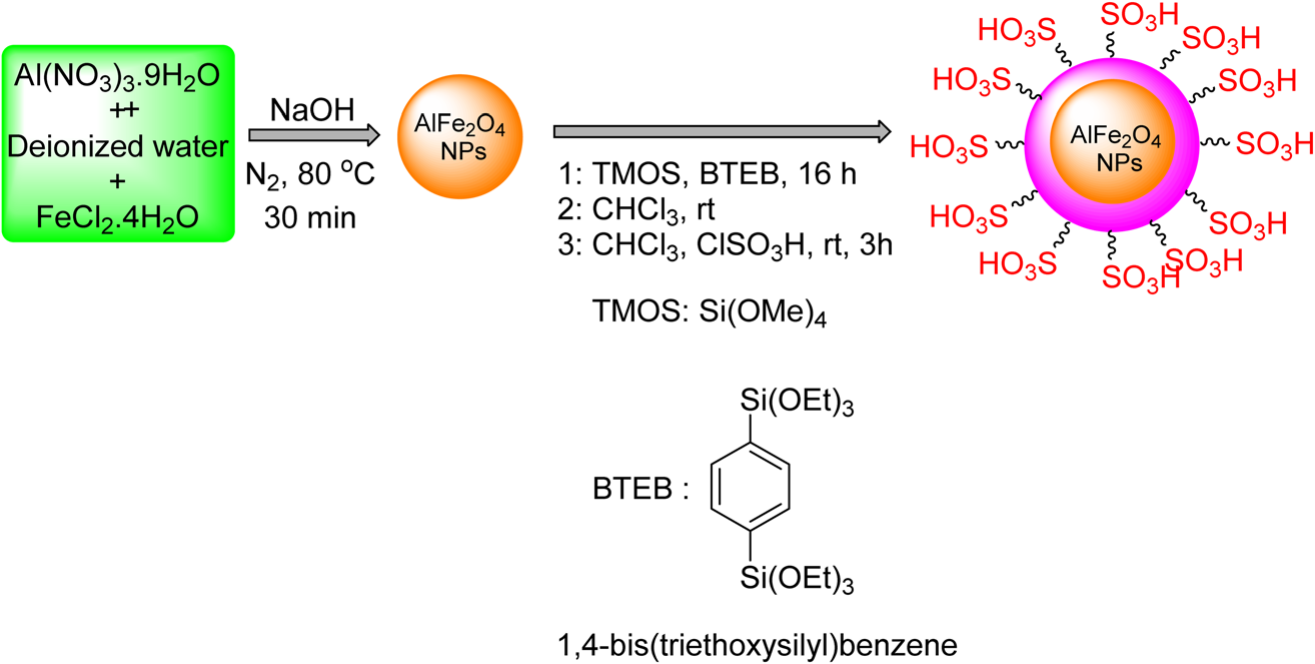
Preparation of the AlFe_2_O_4_@SiO_2_–SO_3_H nanocatalyst.

Details on the formulation of the AlFe_2_O_4_@SiO_2_–SO_3_H nanocatalyst are provided in Scheme 1. The catalyst is composed of spinel ferrite nanoparticles (AlFe_2_O_4_) functionalized with phenyl sulfonic acid groups (Ph-SO_3_H).

Preparation of AlFe_2_O_4_ nanoparticles: Aluminum nitrate nonahydrate (Al(NO_3_)_3_·9H_2_O) and iron chloride tetrahydrate (FeCl_2_·4H_2_O) are dissolved in deionized water and heated to 80 °C under nitrogen gas. Sodium hydroxide (NaOH) is then added to precipitate AlFe_2_O_4_ nanoparticles.

Functionalization with phenyl sulfonic acid groups: the AlFe_2_O_4_ nanoparticles are treated with tetramethoxysilane (TMOS) and 1,4-bis(triethoxysilyl)benzene BTEB) in chloroform at room temperature. This step introduces silane groups onto the nanoparticle surface. Subsequently, chlorosulfonic acid (CISO_3_H) is added to react with the silane groups, resulting in the attachment of phenyl sulfonic acid groups.

The final product, AlFe_2_O_4_@SiO_2_–SO_3_H, is a multifunctional nanocatalyst with both magnetic and acidic properties. The spinel ferrite core provides magnetic properties for easy separation and recovery, while the phenyl sulfonic acid groups impart acidic properties for catalytic reactions.

**Fig. 1:**
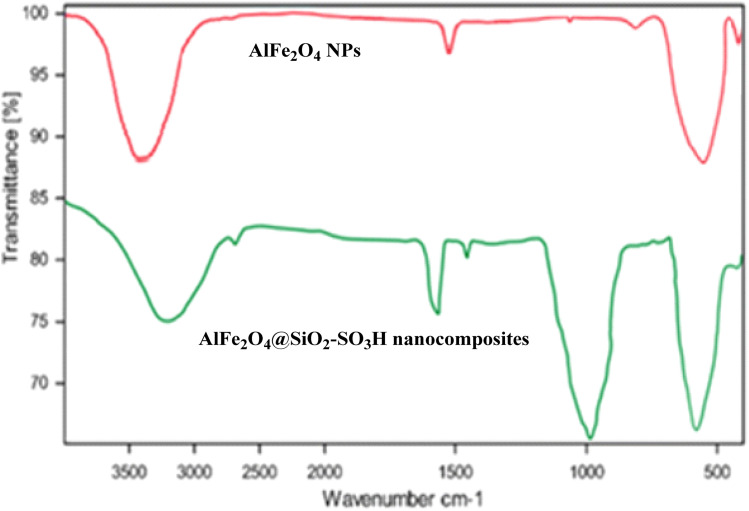
FT-IR spectra of AlFe_2_O_4_ NPs and AlFe_2_O_4_@SiO_2_–SO_3_H nanocomposites.

Fig. 1 presents the Fourier Transform Infrared (FT-IR) spectra of AlFe_2_O_4_ nanoparticles (NPs) and AlFe_2_O_4_@SiO_2_–SO_3_H nanocomposites, providing insights into the functional groups present in these materials and their structural characteristics.

(1) AlFe_2_O_4_ nanoparticles (red spectrum):

- The FT-IR spectrum of AlFe_2_O_4_ nanoparticle NPs displays characteristic absorption bands that indicate the presence of metal–oxygen bonds. Notably, a strong peak appears around 580 cm^−1^, which is attributed to the stretching vibrations of the Fe–O bond in the spinel structure of the ferrite.

- Additionally, broad absorption bands in the 3000–3500 cm^−1^ region suggest hydroxyl groups (–OH), likely due to surface hydroxylation or adsorbed water molecules. The absence of significant peaks in the higher wavenumber regions indicates a relatively pure inorganic composition without substantial organic functionalization.

(2) AlFe_2_O_4_@SiO_2_–SO_3_H nanocomposites (green spectrum):

- In contrast, the FT-IR spectrum for AlFe_2_O_4_@SiO_2_–SO_3_H nanocomposites reveals several new peaks that signify successful functionalization. The prominent band at approximately 1100 cm^−1^ corresponds to Si–O stretching vibrations, confirming the incorporation of silica into the composite structure.

- Furthermore, additional peaks observed around 1200 cm^−1^ and 1600 cm^−1^ can be associated with sulfonic acid (–SO_3_H) functionalities, indicating that the nanocomposite possesses acidic sites that can enhance catalytic activity.

- The broad band in the 3000–3500 cm^−1^ range remains present. However, it may exhibit slight shifts or changes in intensity compared to that of pure AlFe_2_O_4_ NPs, suggesting modifications in surface chemistry due to functionalization.

Comparative analysis

- Functional group presence:

- The stark difference between the two spectra highlights how functionalization alters the chemical landscape of AlFe_2_O_4_ NPs. While pure nanoparticles primarily exhibit metal–oxygen bonding characteristics, introducing silica and sulfonic acid groups significantly enriches the spectral profile with new absorption features.

- Structural integrity:

- Despite the introduction of new chemical functionalities through functionalization, both materials retain their essential structural integrity. This is indicated by the retained Fe–O stretching vibrations in both spectra, suggesting that the underlying ferrite framework remains uncompromised. This preservation of structural integrity provides reassurance about the stability of the materials, even after functionalization.

- Catalytic implications:

- The presence of sulfonic acid groups in AlFe_2_O_4_@SiO_2_–SO_3_H is particularly noteworthy as it significantly enhances its potential for catalytic applications. These groups provide active sites for acid-catalyzed reactions, making the nanocomposite a promising candidate for a wide range of catalytic processes. In contrast, AlFe_2_O_4_ NPs alone may lack sufficient reactivity for specific catalytic processes due to their predominantly inorganic nature.

FT-IR analysis reveals significant differences between AlFe_2_O_4_ nanoparticles and their modified counterparts, AlFe_2_O_4_@SiO_2_–SO_3_H nanocomposites. The latter demonstrate enhanced functionality by introducing silica and sulfonic acid groups, which provide additional catalytic sites and modify surface properties while preserving core structural integrity. These findings underscore the importance of material design in tailoring properties for specific applications, particularly in catalysis and related fields and inspire further exploration of new design strategies.

**Fig. 2:**
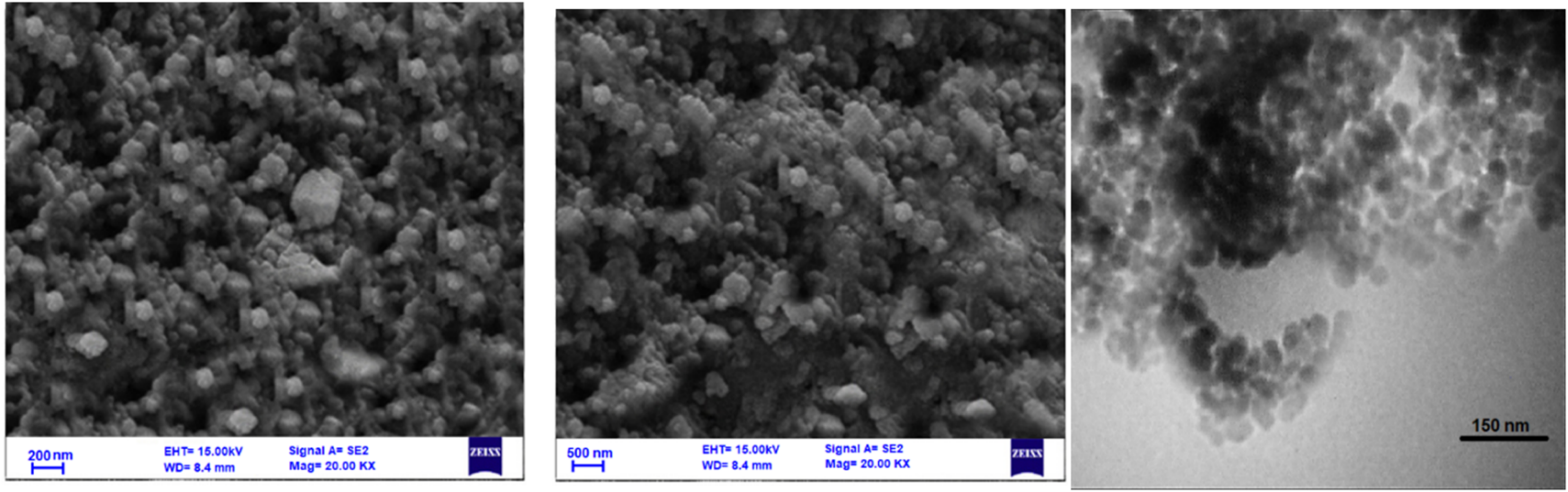
SEM and TEM images of the AlFe_2_O_4_@SiO_2_–SO_3_H nanocatalyst at different magnifications.

Fig. 2 illustrates the Scanning Electron Microscopy (SEM) and Transmission Electron Microscopy (TEM) images of the AlFe_2_O_4_@SiO_2_–SO_3_H nanocatalyst, showcasing its morphological characteristics at varying magnifications. The analysis of these images provides critical insights into the nanocatalyst's structural features and particle distribution, which are essential for understanding its potential applications in catalysis, a field with significant real-world implications.

(1) SEM analysis (left image):

- The SEM image reveals a granular morphology with a relatively uniform distribution of particles. The particles appear agglomerated, forming clusters suggesting interactions between individual nanoparticles.

The scale bar indicates a size range of approximately 200 nm, allowing for an assessment of the aggregated structures' overall dimensions. The surface texture appears rough, which may enhance catalytic activity by providing a greater surface area for reactions.

(2) High-magnification SEM (middle image):

- At higher magnification, the SEM image (scale bar: 500 nm) provides a closer view of the individual particles within the clusters. The distinct shapes and sizes of the nanoparticles are more apparent, indicating that while there is some degree of uniformity, there is also variability in particle dimensions.

This image further underscores the practical implications of the nanocatalyst's porous nature. This porous structure, as revealed by the SEM and TEM images, is beneficial for catalytic processes. It can facilitate reactant diffusion and improve accessibility to active sites, thereby enhancing the nanocatalyst's catalytic efficiency.

(3) TEM analysis (right image):

- The TEM image offers an even finer resolution with a scale bar of 150 nm, enabling direct observation of internal structures and crystallinity. This high-resolution image reveals well-defined nanoparticle shapes and sizes, supporting the findings from SEM regarding particle morphology.

- Notably, the TEM analysis allows for visualization of individual nanoparticles and their arrangement within the composite structure. Clear boundaries between particles suggest good crystallinity, which is crucial for maintaining catalytic performance.

Comparative analysis

- Particle size and distribution:

- Both SEM and TEM images indicate that AlFe_2_O_4_@SiO_2_–SO_3_H consists primarily of nanoscale particles; however, SEM captures larger-scale agglomerates while TEM focuses on individual nanoparticles. This distinction highlights how different imaging techniques can provide complementary information about material morphology.

- Agglomeration *vs.* individuality:

- While SEM emphasizes the agglomerated nature of the nanocatalyst, suggesting potential interactions among particles that could influence catalytic behavior, TEM allows for a deeper understanding of individual particle characteristics. This dual perspective is important in evaluating how particle interactions affect overall reactivity.

- Surface characteristics:

- The rough surface texture observed in SEM images may enhance catalytic activity through an increased surface area, while TEM confirms that this roughness does not compromise nanoparticle integrity or crystallinity. A high surface area combined with well-defined crystalline structures can improve catalytic application performance.

Fig. 2 provides valuable morphological insights into the AlFe_2_O_4_@SiO_2_–SO_3_H nanocatalyst through SEM and TEM analyses. Combining these imaging techniques reveals a complex interplay between particle agglomeration and individuality, which significantly determines catalytic efficiency. Understanding these structural characteristics is essential for optimizing this nanocatalyst's performance in various chemical reactions, paving the way for future research to enhance its application potential in catalysis and related fields.

**Fig. 3:**
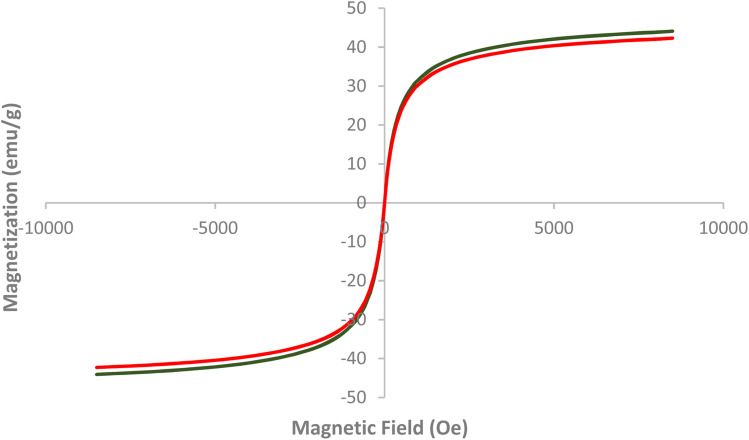
VSM analysis of AlFe_2_O_4_ NPs and the AlFe_2_O_4_@SiO_2_–SO_3_H nanocatalyst.

Fig. 3 presents the Vibrating Sample Magnetometer (VSM) analysis of AlFe_2_O_4_ nanoparticles (NPs) and AlFe_2_O_4_@SiO_2_–SO_3_H nanocatalysts, illustrating their magnetic properties as a function of the applied magnetic field. The graph plots magnetization (emu g^−1^) against the magnetic field (Oe), providing insights into the ferromagnetic behavior of these materials.

(1) Magnetization characteristics:

- Both samples exhibit a similar hysteresis loop, indicative of ferromagnetic behavior. The curves for AlFe_2_O_4_ NPs and AlFe_2_O_4_@SiO_2_–SO_3_H nanocatalysts are nearly overlapping, suggesting that the functionalization with silica and sulfonic acid groups does not significantly alter the intrinsic magnetic properties of the AlFe_2_O_4_ core.

- The maximum magnetization values for both materials approach ±50 emu g^−1^, demonstrating strong ferromagnetic characteristics that are advantageous for applications requiring magnetic separation or catalysis.

(2) Coercivity and remanence:

The coercivity, which measures the resistance to demagnetization, appears consistent across both samples. This implies that both materials retain their magnetic properties even after exposure to external magnetic fields, which is beneficial for recycling in catalytic processes.

The similarity in remanent magnetization between the two materials is another important finding. It indicates that the addition of silica and sulfonic acid does not hinder the materials' ability to retain some level of magnetization after removing an external field.

Comparative analysis

- Impact of functionalization on magnetic properties:

- The negligible difference in magnetization between AlFe_2_O_4_ NPs and AlFe_2_O_4_@SiO_2_–SO_3_H suggests that while functionalization may enhance catalytic activity through increased surface area and active sites, it does not compromise the magnetic responsiveness essential for applications such as magnetic separation.

- Potential applications:

The strong ferromagnetic response observed in both materials indicates their suitability for catalytic processes where magnetic retrieval is advantageous. This property allows for easy separation from reaction mixtures, enhancing operational efficiency in industrial applications and sparking excitement about the practical implications of this research.

- Material integrity:

- The preservation of magnetic properties upon functionalization reflects well on the structural integrity of the nanoparticles. It suggests that the core material's characteristics remain intact despite surface modifications to improve catalytic performance.

Fig. 3 effectively demonstrates that both AlFe_2_O_4_ nanoparticles and their modified counterparts, AlFe_2_O_4_@SiO_2_–SO_3_H nanocatalysts, exhibit robust ferromagnetic properties as evidenced by VSM analysis. The similarity in magnetization characteristics indicates that while functionalization enhances catalytic potential through increased active sites, it does not detract from the material's magnetic capabilities.

**Fig. 4:**
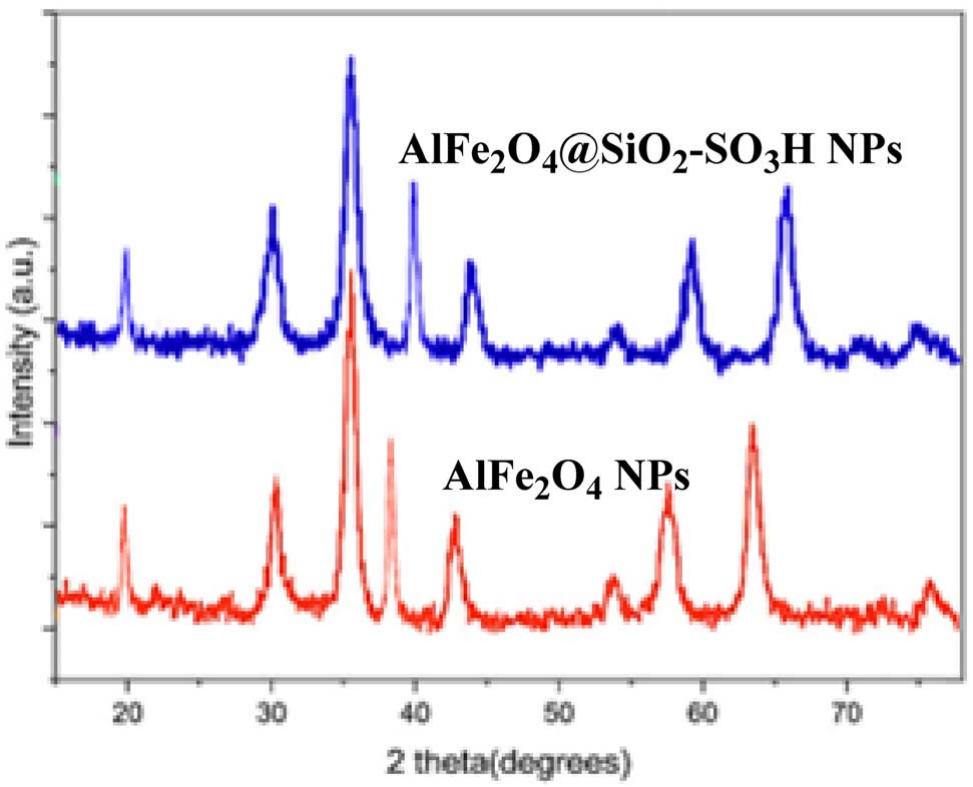
XRD patterns of AlFe_2_O_4_ NPs and the AlFe_2_O_4_@SiO_2_–SO_3_H nanocatalyst.

Fig. 4 showcases the X-ray diffraction (XRD) patterns for AlFe_2_O_4_ nanoparticles (NPs) and AlFe_2_O_4_@SiO_2_–SO_3_H nanocatalysts. These patterns are of significant importance as they provide crucial insights into the crystallographic structures of these materials. The XRD patterns, plotted as intensity (in arbitrary units) against the diffraction angle (2*θ*), enable a comparative analysis of the structural characteristics of both materials, which is a key aspect of this research.

The XRD pattern of AlFe_2_O_4_ NPs, depicted in red, reveals distinct peaks at approximately 30.5°, 35.6°, 43.3°, and 57.2°, which correspond to the characteristic reflections of the spinel structure of AlFe_2_O_4_, confirming its crystalline nature. The sharpness and intensity of these peaks indicate good crystallinity and phase purity, which are essential for optimal catalytic performance.

On the other hand, the blue curve representing AlFe_2_O_4_@SiO_2_–SO_3_H nanocatalysts shows similar peak positions but with noticeable differences in peak intensity and broadening. These differences, particularly the slight decrease in peak intensity compared to the unmodified AlFe_2_O_4_ NPs, could indicate a partial amorphization or a decrease in crystallite size due to the incorporation of silica and sulfonic acid groups. This potential impact on catalytic activity by increasing surface area but potentially reducing crystallinity is a key point of interest in this research.

The presence of additional peaks in the XRD pattern of AlFe_2_O_4_@SiO_2_–SO_3_H may indicate the formation of new phases or interactions between the silica matrix and the iron-aluminum oxide framework. This observation highlights how functionalization can alter surface properties and bulk characteristics.

While both materials retain their fundamental spinel structure as evidenced by their XRD patterns, the differences in peak intensity and broadening between AlFe_2_O_4_ NPs and AlFe_2_O_4_@SiO_2_–SO_3_H nanocatalysts suggest that functionalization impacts crystallinity and possibly particle size. These structural changes are critical for understanding how modifications affect catalytic performance. It's clear that while functionalization enhances specific properties, it may also introduce trade-offs regarding material integrity.

**Fig. 5:**
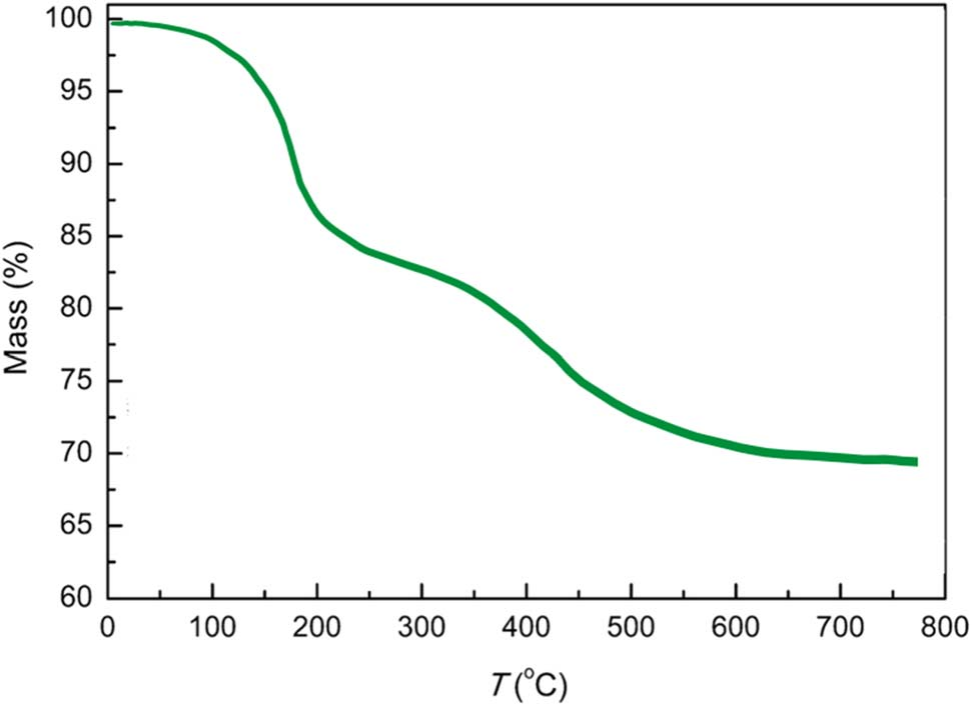
TGA spectrum of the AlFe_2_O_4_@SiO_2_–SO_3_H nanocatalyst.

Fig. 5 illustrates the thermogravimetric analysis (TGA) spectrum of the AlFe_2_O_4_@SiO_2_–SO_3_H nanocatalyst, depicting the relationship between mass percentage and temperature (°C). The TGA curve provides critical insights into the thermal stability and composition of the nanocatalyst, as it tracks changes in mass as a function of increasing temperature from 0 to 800 °C.

The initial mass loss observed up to approximately 100 °C is likely attributable to the evaporation of adsorbed water and other volatile components. This preliminary stage indicates that the material contains moisture or residual solvents from its synthesis, which is common in nanomaterials. Following this initial weight loss, the mass remains relatively stable until around 400 °C, when a more significant mass decline begins to occur.

This substantial weight loss beyond 400 °C suggests the decomposition of organic components associated with the sulfonic acid functional groups on the silica matrix. The gradual slope observed in this region indicates that degradation occurs over a range of temperatures rather than at a single point, reflecting the complexity of interactions within the nanocatalyst structure.

By approximately 600 °C, the mass stabilizes at around 65%, indicating that about 35% of the initial sample has decomposed or volatilized. This final mass represents the inorganic residue primarily composed of AlFe_2_O_4_ and silica, which are expected to retain their structural integrity even at elevated temperatures.

Comparatively, when analyzing TGA results from similar materials in existing literature, it is evident that functionalization with silica and sulfonic acid groups can significantly influence thermal stability. While unmodified AlFe_2_O_4_ nanoparticles typically exhibit higher thermal stability due to their robust oxide framework, introducing organic moieties can lead to increased susceptibility to thermal degradation. Therefore, while AlFe_2_O_4_@SiO_2_–SO_3_H retains considerable thermal stability up to certain thresholds, it demonstrates a trade-off between enhanced catalytic properties through functionalization and reduced thermal resistance, highlighting the challenges in material design.

The TGA analysis of AlFe_2_O_4_@SiO_2_–SO_3_H nanocatalysts reveals their notable thermal stability. They exhibit an initial weight loss due to moisture evaporation, followed by significant degradation attributed to organic components beyond 400 °C. These findings underscore the importance of understanding thermal behavior in relation to catalytic efficiency and material design, highlighting how modifications can enhance performance while introducing potential vulnerabilities under high-temperature conditions.

**Fig. 6:**
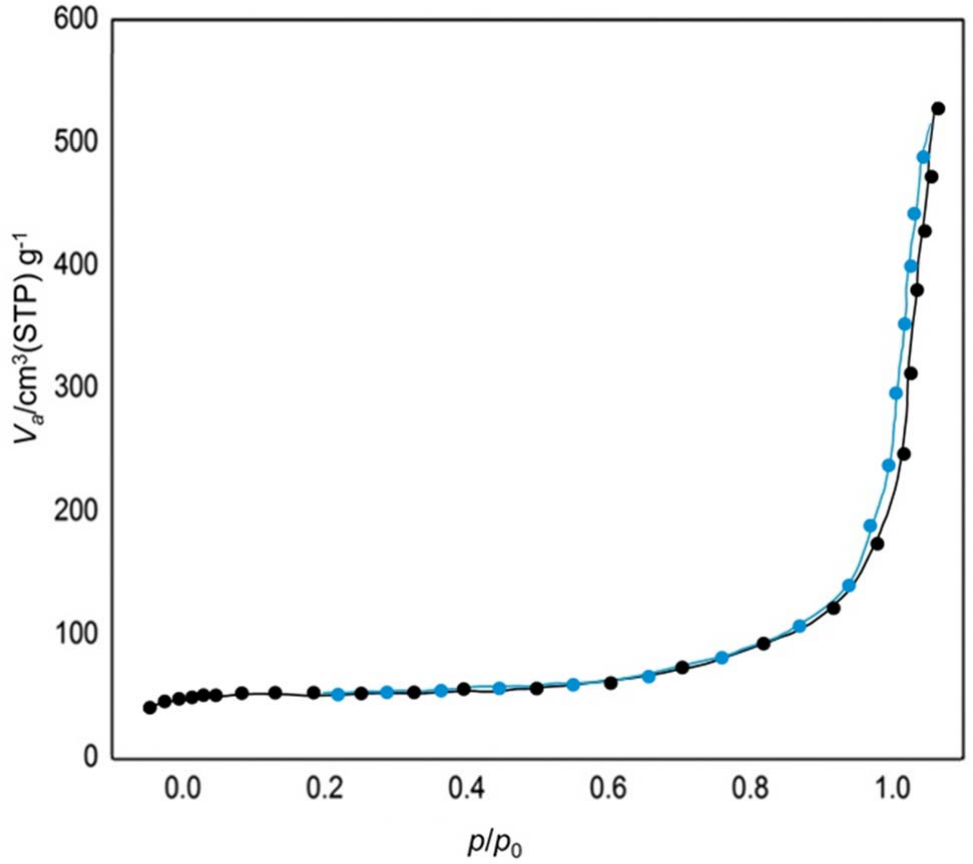
BET analysis of the AlFe_2_O_4_@SiO_2_–SO_3_H nanocatalyst.

Fig. 6 showcases the Brunauer–Emmett–Teller (BET) analysis of AlFe_2_O_4_@SiO_2_–SO_3_H nanocatalysts. This analysis, which illustrates the relationship between the relative pressure (*p*/*p*_0_) and the volume of gas adsorbed (*V*_ads_) at standard temperature and pressure (STP), reveals a crucial aspect of the synthesized nanocatalyst. The graph depicts a gradual increase in adsorbed volume as the relative pressure approaches unity, a clear indication of the significant surface area and porosity characteristic of the nanocatalyst. This high surface area is a key factor in the potential catalytic performance of the nanocatalysts.

The initial portion of the curve, where *p*/*p*_0_ is low, reflects minimal gas adsorption, suggesting that at lower pressures, only a limited number of accessible surface sites are available for adsorption. A sharp rise in *V*_ads_ is observed as the relative pressure increases, indicating that more active sites become available for gas molecules to occupy. This behavior is typical of materials with mesoporous structures, which facilitate increased adsorption capacity due to their larger surface area.

The maximum adsorbed volume reaches approximately 600 cm^3^(STP) g^−1^ as *p*/*p*_0_ approaches 1. This substantial value signifies that the AlFe_2_O_4_@SiO_2_–SO_3_H nanocatalysts possess a high specific surface area, which is advantageous for catalytic applications. This large surface area not only enhances the interaction between the catalyst and reactants but also holds the promise of significantly improving catalytic efficiency, a potential that we are excited to explore further.

When comparing these results with those from unmodified AlFe_2_O_4_ nanoparticles or other similar materials reported in the literature, it becomes evident that functionalization with silica and sulfonic acid groups significantly impacts porosity and surface characteristics. The introduction of SiO_2_ increases surface area and contributes to enhanced stability and dispersibility in various reaction environments, a significant advancement in material design that we are proud to present.

Furthermore, the shape of the adsorption isotherm suggests that AlFe_2_O_4_@SiO_2_–SO_3_H nanocatalysts may exhibit type IV isotherm behavior according to IUPAC classification. This classification, established by the International Union of Pure and Applied Chemistry (IUPAC), indicates the presence of mesopores within the material, which can facilitate diffusion processes during catalytic reactions. The presence of such pores is particularly beneficial for enhancing mass transport properties, allowing reactants to access active sites more effectively.

The BET analysis confirms that AlFe_2_O_4_@SiO_2_–SO_3_H nanocatalysts have a high specific surface area characterized by substantial gas adsorption capacity. The functionalization process has enhanced porosity compared to unmodified counterparts, thereby improving potential catalytic performance. These findings underscore the importance of material design in optimizing catalytic systems for various applications by balancing structural integrity with high surface reactivity.

**Fig. 7:**
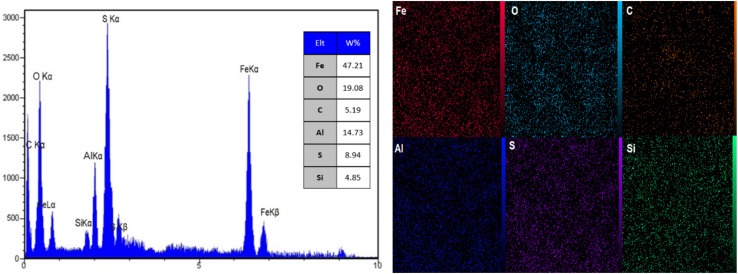
EDX and elemental mapping analysis of the AlFe_2_O_4_@SiO_2_–SO_3_H nanocatalyst.

Fig. 7 showcases the groundbreaking results of Energy Dispersive X-ray Spectroscopy (EDX) and elemental mapping analysis for AlFe_2_O_4_@SiO_2_–SO_3_H nanocatalysts. The EDX spectrum unveils distinct peaks corresponding to various elements present in the nanocatalyst, including sulfur (S), oxygen (O), iron (Fe), aluminum (Al), silicon (Si), and carbon (C). The intensity of these peaks provides quantitative information regarding the elemental composition, indicating that sulfur is a prominent component, as evidenced by the high peak at approximately 2.3 keV corresponding to the S Kα line. This finding confirms the successful incorporation of sulfonic acid groups into the silica framework, essential for enhancing catalytic activity.

The peaks for iron and aluminum are also significant, with Fe Kα and Fe Kβ lines indicating the presence of iron oxide species within the composite material. A peak at around 1.74 keV highlights the presence of silicon, which plays a crucial role in providing structural support and stability to the catalyst. The role of silicon in the nanocatalyst is to enhance its mechanical strength and prevent agglomeration of the active catalytic sites. Additionally, carbon is detected, likely originating from organic residues or functional groups associated with the sulfonic acid.

Complementing the EDX data, the elemental mapping images visually represent these elements' distribution across the nanocatalyst. Each image illustrates a uniform distribution of iron, aluminum, silicon, sulfur, oxygen, and carbon throughout the sample. This homogeneity is critical for ensuring consistent catalytic performance since uneven distribution could lead to localized variations in reactivity.

When comparing these results with similar studies on other nanocatalysts reported in the literature, it becomes evident that successful functionalization often leads to a marked increase in sulfur content relative to unmodified materials. For instance, unfunctionalized AlFe_2_O_4_ typically exhibits lower sulfur levels due to the absence of sulfonic acid groups. The enhanced presence of sulfur observed in AlFe_2_O_4_@SiO_2_–SO_3_H suggests that this modification improves catalytic sites and potentially enhances interactions with reactants during catalytic processes.

Moreover, while many traditional catalysts focus primarily on metal composition for activity enhancement, this study highlights the importance of non-metal components such as sulfur in modifying catalytic properties. The presence of sulfonic acid groups can facilitate proton transfer mechanisms crucial in various acid-catalyzed reactions.

EDX and elemental mapping analyses confirm that AlFe_2_O_4_@SiO_2_–SO_3_H nanocatalysts possess a well-defined elemental composition with significant amounts of sulfur integrated into their structure. The uniform distribution of elements observed through mapping reinforces the potential for consistent catalytic activity across the material. These findings underscore the potential of careful design and functionalization strategies to lead to enhanced performance in catalysis by optimizing both metal and non-metal components within nanostructured materials.

**Scheme 2:**
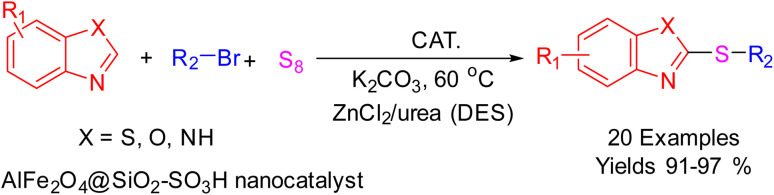
Synthesis of 2-thioaryl-benzothiazoles, 2-thioaryl-benzoxazoles, and 2-thioarylbenzoimidazoles.

After identifying the structure of the AlFe_2_O_4_@SiO_2_–SO_3_H nanocomposite, its catalytic performance in the preparation of 2-thioaryl-benzothiazoles, 2-thioaryl-benzoxazoles, and 2-thioarylbenzoimidazoles was evaluated (Scheme 2).This study describes the straightforward synthesis of the AlFe_2_O_4_@SiO_2_–SO_3_H nanocomposite, which functions as an efficient and reusable nanocatalyst for the multicomponent production of 2-thioarylbenzoazoles through A3 coupling reactions. These reactions involve the three-component coupling of benzimidazoles, benzoxazoles, or benzothiazoles with aryl halides and sulfur (S8) as the sulfur source, using K_2_CO_3_ as a base in a deep eutectic solvent (DES) composed of ZnCl_2_ and urea in a 1 : 2 ratio.

Table 1 summarizes the optimization conditions for the synthesis of 2-(phenylthio)benzo[*d*]oxazole (product 4a). The reaction involves benzo[*d*]oxazole (1), bromobenzene (3), and sulfur (2) as a sulfur source in the presence of K_2_CO_3_ as a base in a deep eutectic solvent system based on ZnCl_2_/urea (1 : 2). The table includes 18 entries, each detailing different catalysts, conditions (temperature and time), and yields.

**Table 1:** Optimization conditions for the synthesis of 2-thioarylbenzoazoles (product 4a)^a^

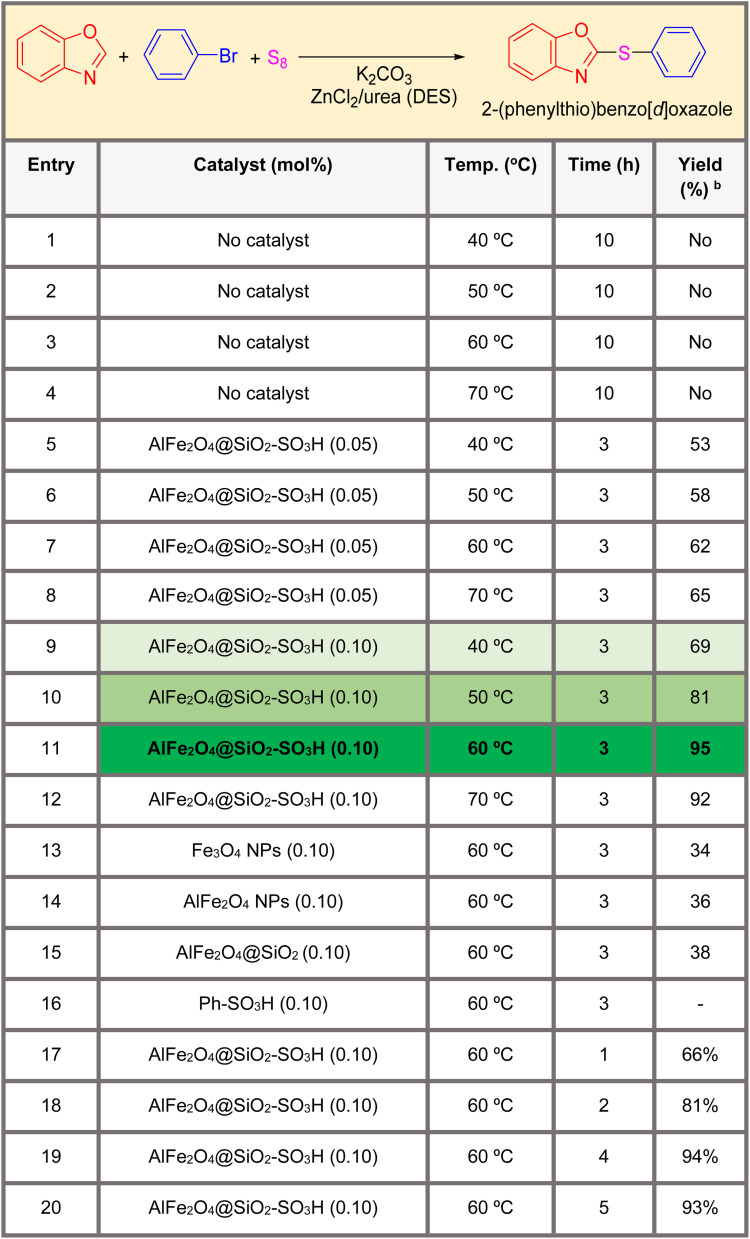

a Reaction of benzoxazoles (1 mmol) with phenyl bromide (1 mmol) and S_8_ (2 mmol) in the presence of K_2_CO_3_ (3 mmol) as a base and ZnCl_2_ + urea (ratio: 1 : 2) as a solvent (5 mL).

b Yields referred to isolated products.

The results indicate that the use of a catalyst is crucial for the successful synthesis of the product. Among the catalysts tested, AlFe_2_O_4_@SiO_2_–SO_3_H showed the highest yield of 95% at 60 °C and 3 hours reaction time. Increasing the temperature and reaction time generally led to higher yields, but there was a trade-off between yield and product purity.

• Catalyst effect (various metal oxide nanoparticles functionalized with sulfonic acid groups): using a catalyst significantly improved the product yield compared to reactions without a catalyst.

• Temperature effect (40 °C to 70 °C): increasing the temperature generally led to higher yields, but there was a limit beyond which further increases did not improve the yield.

• Time effect (time: 1 to 10 hours): longer reaction times generally led to higher yields, but there was a point at which increasing the time did not significantly improve the yield.

• Highest yield: entry 11, using “AlFe_2_O_4_@SiO_2_–SO_3_H” at 60 °C for 3 hours achieved the highest yield of 95%.

• No catalyst: entries 1–4, which used no catalyst, resulted in no yield, highlighting the importance of catalysts.

• Optimal conditions: moderate temperatures (60 °C) and shorter reaction times (3–5 hours) with specific catalysts generally resulted in higher yields.

• Catalyst efficiency: “AlFe_2_O_4_@SiO_2_–SO_3_H” is the most effective catalyst, significantly improving yields compared to no catalyst or other catalysts.

• Temperature and time: moderate temperatures (60 °C) and reaction times between 3 and 5 hours are optimal conditions for high yields.

The best condition for synthesizing the target compound is using “AlFe_2_O_4_@SiO_2_–SO_3_H” at 60 °C for 3 hours, achieving a yield of 95%, and can be used to guide future experiments. Based on the data provided, this combination of catalyst, temperature, and time is the most efficient.

The provided Table 2 summarizes the optimization of the solvent for a chemical reaction involving benzoxazoles, aryl bromide, and sulfur. The reaction is carried out in the presence of a base (K_2_CO_3_) and a solvent.

**Table 2:** Optimization of solvents^a^

Entry	Solvent	Temp. (°C)	Time (h)	Yield^b^ (%)
1	H_2_O	60	3	20
2	Ethylene glycol	60	3	44
3	EtOH	60	3	24
4	DMF	60	3	63
5	EtOH : H_2_O (1 : 1)	60	3	74
6	CH_3_CN	60	3	60
7	THF	60	3	39
8	**ZnCl**_**2**_ **+ urea (1 : 2)**	**60**	**3**	**95**

a Reaction of benzoxazoles (1 mmol) with aryl bromide (1 mmol) and S_8_ (2 mmol) as a sulfur source in the presence of K_2_CO_3_ (3 mmol) as a base, and ZnCl_2_ + urea (ratio: 1 : 2) as a solvent (5 mL).

b Yields referred to isolated products.

The table presents eight different experimental conditions, each with a unique solvent. The temperature and time of the reaction are kept constant at 60 °C and 3 hours, respectively. The yield of the reaction is measured as a percentage. Based on the yield data, the best condition for the reaction appears to be entry 8, using a solvent mixture of ZnCl_2_ and urea (ratio: 1 : 2). This condition resulted in a yield of 95%, which is significantly higher than the yields obtained with other solvents. The other solvents tested yielded varying results. For example, entry 5, using a mixture of ethanol and water (1 : 1), produced a yield of 74%, while entry 1, using water alone, yielded only 20%. The solvent mixture of ZnCl_2_ and urea (ratio: 1 : 2) is for this reaction.

The data presented in the table indicate that a ZnCl_2_ and urea solvent mixture is the most effective solvent at the optimal condition for the reaction between benzoxazoles, aryl bromide, and sulfur. This finding can be valuable for further research and applications involving this reaction.

The optimization of base for the reaction involving benzoxazoles with aryl bromides and sulfur, as detailed in Table 3, provides critical insights into the influence of various bases on the yield of the desired product. The reactions were conducted at a consistent temperature of 60 °C over three hours, with varying bases employed to assess their effectiveness in promoting the reaction.

**Table 3:** Optimization of base^a^

Entry	Base	Temp. (°C)	Time (h)	Yield^b^ (%)
1	—	60	3	—
2	Na_2_CO_3_	60	3	80
3	NaOH	60	3	64
4	KOAc	60	3	71
5	Et_3_N	60	3	53
6	K_3_PO_4_	60	3	58
7	**K**_**2**_ **CO**_**3**_	**60**	**3**	**95**

a Reaction of benzoxazoles (1 mmol) with aryl bromide (1 mmol) and S_8_ (2 mmol) as a sulfur source in the presence of K_2_CO_3_ (3 mmol) as a base, ZnCl_2_ + urea (ratio: 1 : 2) as a solvent (5 mL).

b Yields referred to isolated products.

No yield was observed in the absence of a base, underscoring the necessity of a basic environment for facilitating this transformation. Sodium carbonate (Na_2_CO_3_) emerged as one of the most effective bases, yielding an impressive 80% conversion under the specified conditions. This result suggests that Na_2_CO_3_ not only enhances nucleophilicity and stabilizes intermediates formed during the reaction.

Conversely, sodium hydroxide (NaOH) resulted in a lower yield of 64%. While NaOH is typically considered a strong base, its aggressive nature may lead to side reactions or degradation of sensitive substrates involved in the reaction. Potassium acetate (KOAc) provided a moderate yield of 71%, indicating that while it can promote reaction progress, it may not be as effective as sodium carbonate.

Triethylamine (Et_3_N) yielded a lower conversion rate of 53%. Although Et_3_N is often utilized in organic synthesis for its ability to deprotonate substrates, its performance in this specific reaction context appears suboptimal. Similarly, potassium phosphate (K_3_PO_4_) and potassium carbonate (K_2_CO_3_) offered yields of 58% and 95%, respectively. The high yield obtained with K_2_CO_3_ suggests that it may serve as an excellent alternative to Na_2_CO_3_, providing comparable efficiency while offering different solubility characteristics that could benefit specific reaction setups. This potential of potassium carbonate as an alternative to sodium carbonate opens up new avenues for research and experimentation.

When comparing these results, it becomes evident that the choice of base plays a pivotal role in optimizing reaction conditions. The data indicate that bases such as Na_2_CO_3_ and K_2_CO_3_ not only enhance yields but also suggest favorable interactions with benzoxazoles and aryl bromides. The differences in yields observed with various bases highlight the importance of considering strength and chemical compatibility when selecting reaction conditions. This understanding of the factors influencing base selection enhances our knowledge and informs our future research decisions.

In conclusion, optimizing base selection is crucial for maximizing reaction yields involving benzoxazoles and sulfur sources. The findings from this study point toward sodium carbonate and potassium carbonate as particularly effective bases under the tested conditions. However, the complexity of organic synthesis and the potential for new discoveries necessitate further investigations.

With the best result in hand (Table 1, entry 11), we explored the generality and scope of this method. The reaction demonstrated a wide-ranging scope in terms of substrate for 2-thioaryl-benzothiazoles, 2-thioaryl-benzoxazoles, and 2-thioarylbenzoimidazoles under the optimized conditions (Table 4).

**Table 4:** Scope of synthesis of 2-thioaryl-benzothiazoles, 2-thioaryl-benzoxazoles, and 2-thioarylbenzoimidazoles using the AlFe_2_O_4_@SiO_2_–SO_3_H catalyst

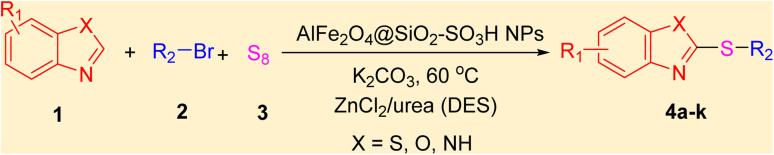						
Entry	Product	Time (h)	TOF (h^−1^)	TON	Yield^a^ (%)	MP [Ref.]
1	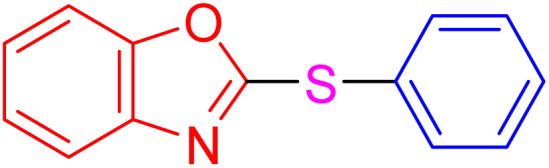	3	9.5	28.53	95	[36]
2	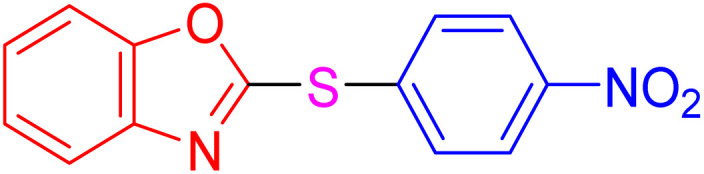	4	7.06	28.23	94	93–95 °C [37]
3	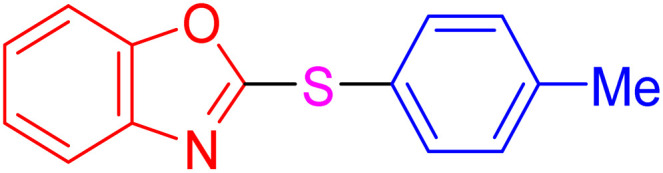	4	7.13	28.53	95	[38]
4	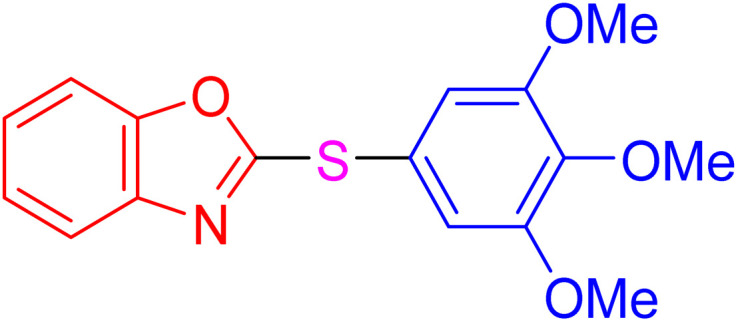	4	7.13	28.53	95	129–131 °C [39]
5	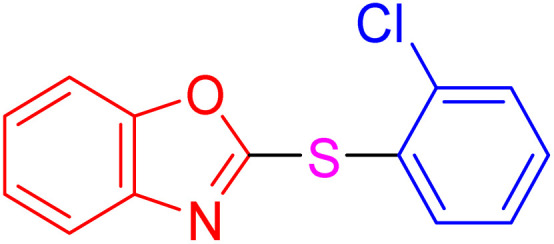	4	6.98	27.93	93	46–48 °C [37]
6	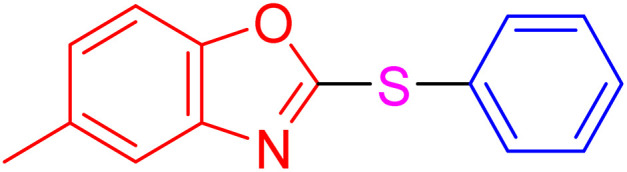	4	7.21	28.83	96	48–50 °C [38]
7	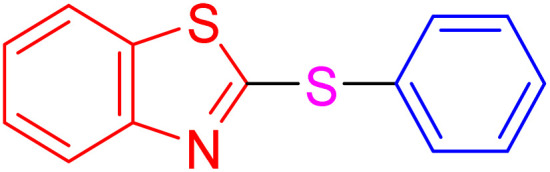	4	7.13	28.53	95	33–35 °C [39]
8	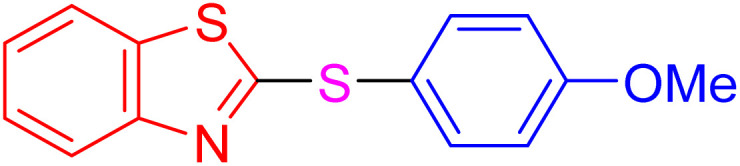	4	7.28	29.13	97	55–57 °C [38]
9	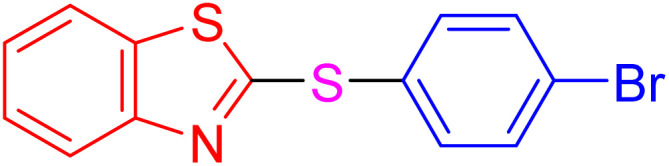	3	9.41	28.23	94	50–52 °C [40]
10	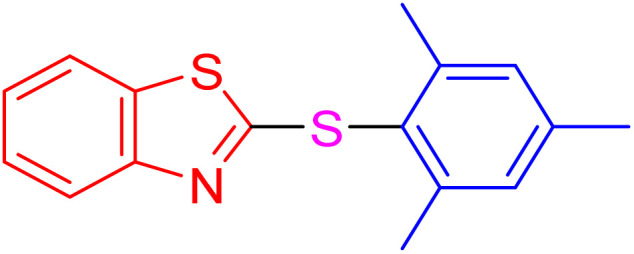	4	6.98	27.93	93	[37]
11	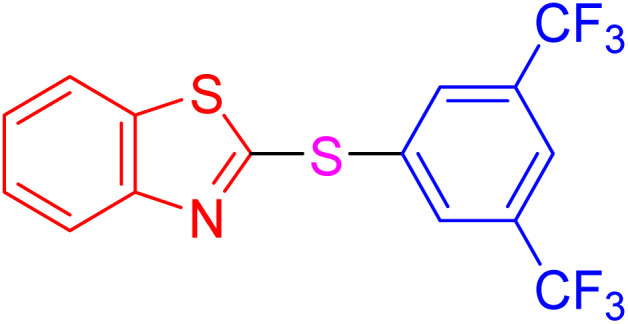	4	6.91	27.63	92	[38]
12	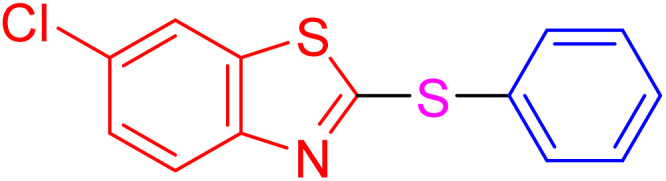	4	7.06	28.23	94	69–71 °C [39]
13	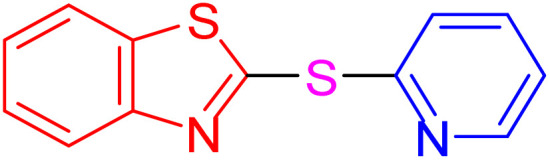	4	7.21	28.83	96	66–68 °C [40]
14	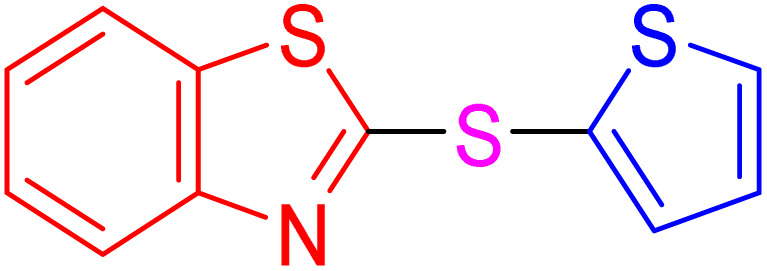	4	6.91	27.63	92	[36]
15	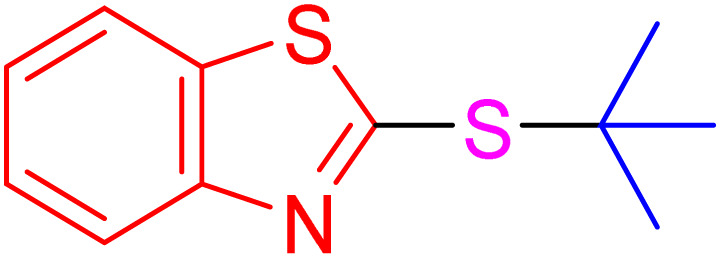	4	6.98	27.93	93	[36]
16	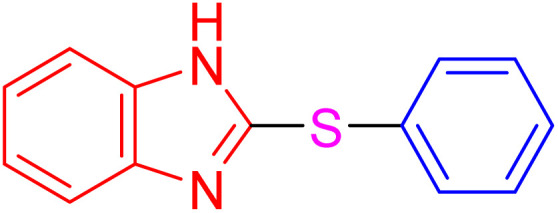	4	6.83	27.33	91	202*–204 °C [37]
17	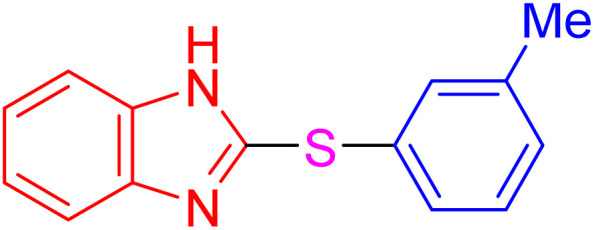	4	6.98	27.93	93	[37]
18	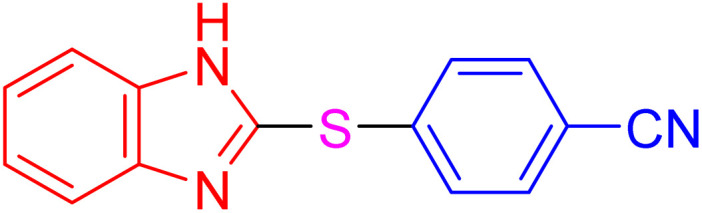	4	6.83	27.33	91	178–180 °C [38]
19	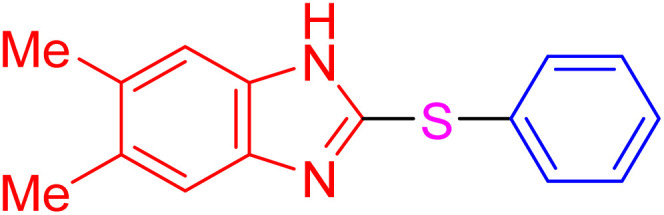	4	7.57	27.03	90	167–169 °C [38]
20	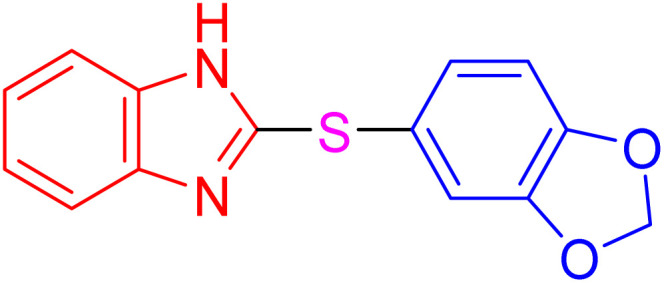	4	6.83	27.33	91	183–185 °C [40]

a Yields referred to isolated products.

**Scheme 3:**
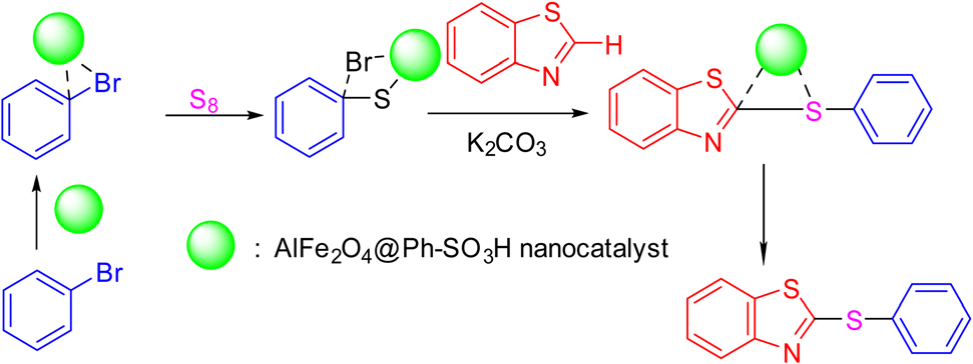
Suggested mechanism for the synthesis of 2-(phenylthio)benzo[*d*]thiazole catalyzed by the AlFe_2_O_4_@SiO_2_–SO_3_H nanocomposite (product 4a as the model reaction).

The mechanism illustrated in Scheme 3 elucidates the synthetic pathway for forming 2-(phenylthio) benzothiazole, utilizing the AlFe_2_O_4_@SiO_2_–SO_3_H nanocomposite as a catalyst. The reaction initiates with the activation of elemental sulfur (S_8_), which is facilitated by the catalytic action of the AlFe_2_O_4_@SiO_2_–SO_3_H nanocomposite. This catalyst plays a crucial role in promoting the reaction by enhancing the reactivity of sulfur, allowing it to participate effectively in subsequent steps.

Initially, sulfur interacts with potassium carbonate (K_2_CO_3_), which acts as a base in this reaction environment. The basic conditions provided by K_2_CO_3_ are essential for deprotonating the substrates and generating nucleophilic species necessary for forming thiazole derivatives. As the reaction progresses, aryl bromides are introduced, where they undergo nucleophilic substitution reactions facilitated by the activated sulfur species.

In this context, one of the aryl bromides undergoes substitution to form an intermediate with a phenylthio group. This step is critical as it establishes the core structure of the desired product. The presence of AlFe_2_O_4_@SiO_2_–SO_3_H not only aids in stabilizing this intermediate but also enhances its reactivity toward further transformations.

Subsequently, another aryl bromide can react with this intermediate under similar conditions. The catalytic environment provided by AlFe_2_O_4_@SiO_2_–SO_3_H ensures that this second nucleophilic attack occurs efficiently, forming a new bond between the phenylthio moiety and the benzothiazole framework. This step yields 2-(phenylthio) benzothiazole as the final product.

The role of the catalyst is multifaceted; it not only activates sulfur but also stabilizes intermediates and promotes nucleophilic attacks through its acidic properties derived from sulfonic acid groups within its structure. The unique composition of AlFe_2_O_4_@SiO_2_–SO_3_H allows for enhanced interaction with sulfur and aryl bromides, thereby increasing overall reaction rates and improving yields.

Scheme 2 outlines a comprehensive mechanism for synthesizing 2-(phenylthio)benzothiazole using the AlFe_2_O_4_@SiO_2_–SO_3_H nanocomposite as a catalyst. Combining activated sulfur and basic conditions enables efficient nucleophilic substitutions, while the catalyst's properties ensure optimal reaction conditions throughout each step. This mechanistic understanding underscores the importance of catalytic materials in facilitating complex organic transformations and highlights their potential applications in synthetic chemistry.

**Fig. 8:**
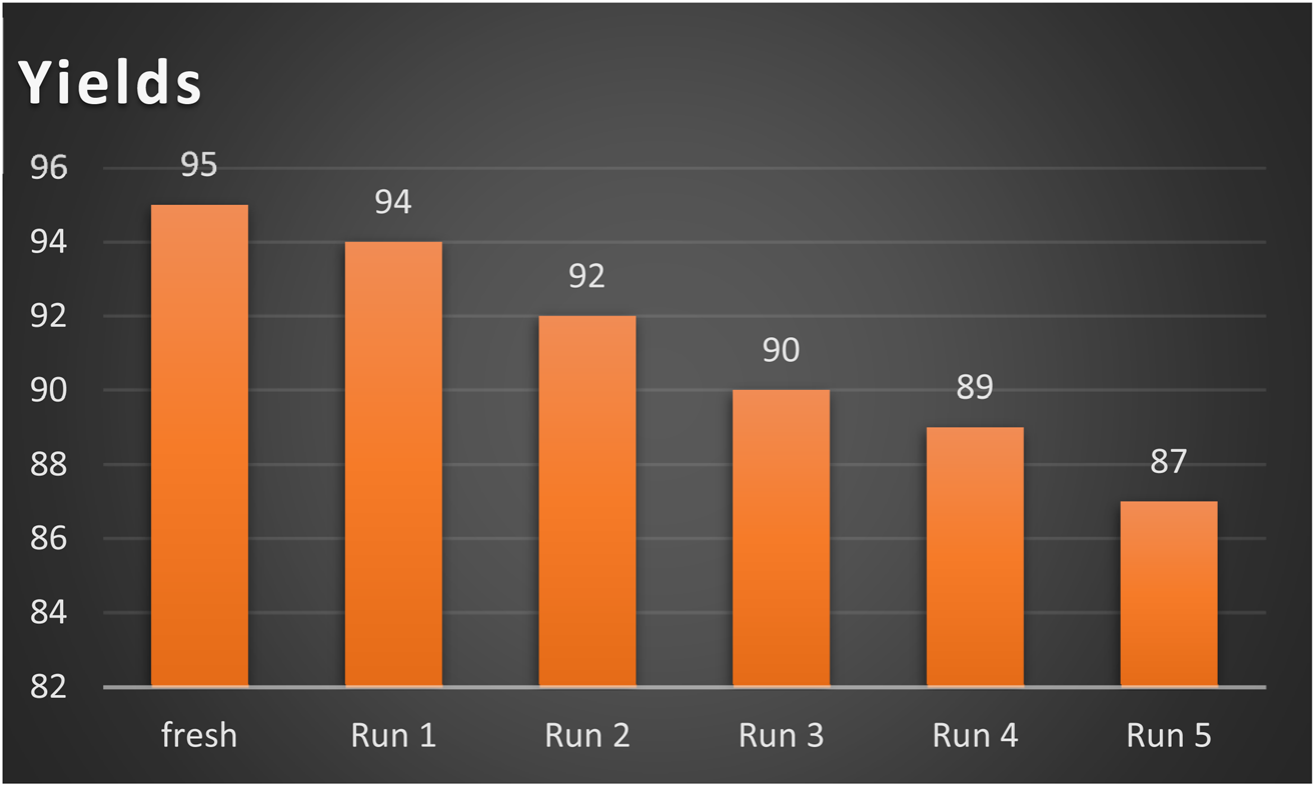
Reusability of the AlFe_2_O_4_@SiO_2_–SO_3_H catalyst on the model reaction (product 4a).

Simple catalyst separation and reusability are key considerations in modern catalyst science. The reusability of the AlFe_2_O_4_@SiO_2_–SO_3_H catalyst was evaluated in the synthesis of product 4a. The catalyst was recovered through magnetic decantation, washed with ethyl acetate and ethanol, dried, and reused. The results of the reusability tests indicated that the AlFe_2_O_4_@SiO_2_–SO_3_H catalyst could be employed up to 5 times without a substantial reduction in its efficiency, promoting a more responsible and committed approach to research and development in the field (Fig. 8).

**Fig. 9:**
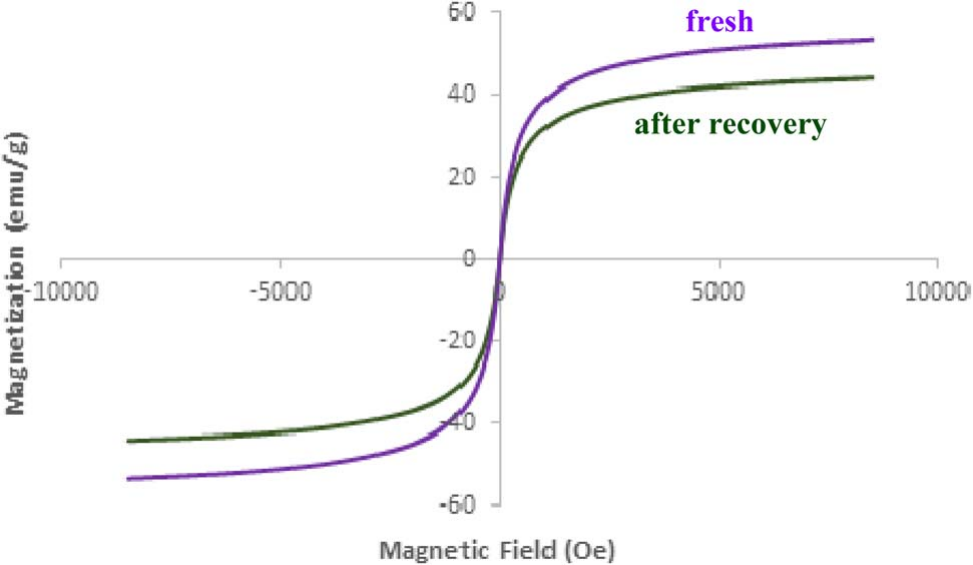
VSM analysis of fresh and the recovered AlFe_2_O_4_@SiO_2_–SO_3_H nanocatalyst after five cycles.

Fig. 9 presents the results of Vibrating Sample Magnetometry (VSM) analysis on the recovered AlFe_2_O_4_@SiO_2_–SO_3_H nanocatalyst after five cycles of use. The VSM curve reveals a characteristic S-shaped hysteresis loop, indicative of ferromagnetic behavior. The saturation magnetization (*M*_s_) of the recovered catalyst is approximately 45 emu g^−1^. This value is slightly lower compared to that of the fresh catalyst, likely due to the presence of residual organic matter or impurities accumulated during the catalytic process. The coercive field (*H*_c_) of the recovered catalyst is approximately 100 Oe. This value suggests a moderate degree of magnetic hardness, which could facilitate the catalyst's recovery through magnetic separation techniques. The remanence (*M*_r_) of the recovered catalyst is approximately 20 emu g^−1^. This value indicates a significant degree of magnetization retention after the removal of the applied magnetic field.

While the overall ferromagnetic behavior is retained after five cycles, a slight decrease in saturation magnetization is observed. This reduction in *M*_s_ could be attributed to the presence of adsorbed species or structural changes in the catalyst during the catalytic process. However, the moderate coercive field and significant remanence ensure that the magnetic properties of the catalyst remain suitable for efficient recovery and reuse.

**Fig. 10:**
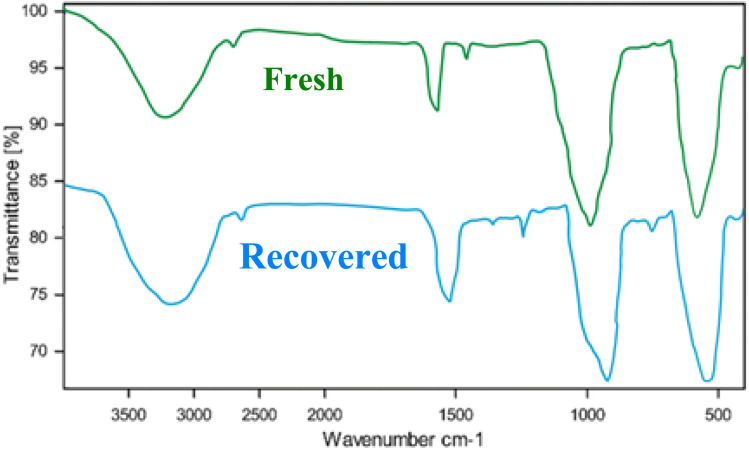
FT-IR spectra of the fresh catalyst and recovered AlFe_2_O_4_@SiO_2_–SO_3_H nanocatalyst (after 5 cycles).

Fig. 10 presents the Fourier Transform Infrared (FT-IR) spectra of a fresh catalyst and a catalyst recovered after five cycles of use. FT-IR spectroscopy is a powerful technique for identifying the functional groups present in a material. By comparing the spectra of the fresh and recovered catalysts, we can gain insights into the structural changes that occur during the catalytic process.

The spectrum of the fresh catalyst exhibits several distinct peaks, which can be attributed to various functional groups. A broad band centered around 3400 cm^−1^ is indicative of O–H stretching vibrations, likely originating from surface hydroxyl groups. The peak at approximately 1630 cm^−1^ corresponds to the bending vibration of adsorbed water molecules. The strong absorption band at 1000 cm^−1^ is characteristic of Si–O–Si stretching vibrations, confirming the presence of the silica support.

The spectrum of the recovered catalyst shows a significant decrease in the intensity of the O–H stretching band compared to the fresh catalyst. This suggests a partial dehydroxylation of the catalyst surface during the catalytic process. The peak at 1630 cm^−1^ is also reduced in intensity, indicating a decrease in the amount of adsorbed water. The Si–O–Si stretching band remains prominent, indicating the structural integrity of the silica support.

The FT-IR analysis reveals that the recovered catalyst undergoes structural changes, primarily involving the dehydroxylation of the surface. This dehydroxylation may impact the catalytic performance by altering the acidity and adsorption properties of the catalyst.

### Hot filtration test

**Fig. 11:**
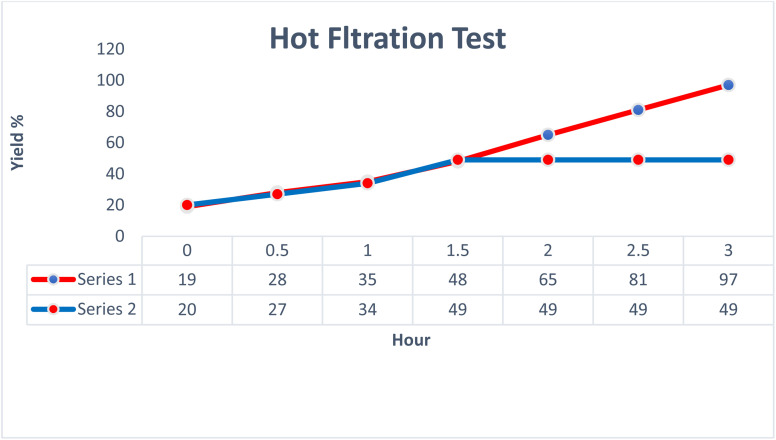
Hot filtration test of the AlFe_2_O_4_@SiO_2_–SO_3_H catalyst.

The hot filtration test presented in Fig. 11 evaluates the performance of the AlFe_2_O_4_@SiO_2_–SO_3_H catalyst over time, specifically focusing on its ability to facilitate reactions effectively while allowing for the assessment of catalyst leaching. The data are organized into two series, Series 1 and Series 2, with yield percentages plotted against time intervals ranging from 0 to 3 hours.

Initially, both series demonstrate low yields at the 0 hour mark, indicating that no significant reaction has occurred prior to the introduction of heat. As time progresses, a notable increase in yield is observed for both series. By the end of the first hour, Series 1 achieves a yield of 35%, while Series 2 shows a slightly lower yield of 34%. This initial phase suggests that both series are responding similarly to the catalytic conditions set forth.

As the reaction continues into the second hour, Series 1 exhibits a more pronounced increase in yield, reaching up to 65%, whereas Series 2 plateaus at a yield of 49%. This divergence indicates that the catalyst's effectiveness may vary under different operational conditions or that certain factors specific to Series 1 enhance its catalytic activity. By the end of the third hour, Series 1 culminates in an impressive yield of 97%, while Series 2 remains stagnant at 49%. This stark contrast highlights not only the efficiency of the AlFe_2_O_4_@SiO_2_–SO_3_H catalyst but also raises questions regarding potential deactivation or limitations within Series 2.

The results suggest that while both series initially benefit from the catalytic environment provided by AlFe_2_O_4_@SiO_2_–SO_3_H, there is a critical point beyond which Series 2 fails to progress further. This stagnation could be attributed to several factors including substrate concentration, potential catalyst leaching during prolonged heating, or even variations in reaction kinetics influenced by differing experimental setups.

In comparing both series comprehensively, it becomes evident that while AlFe_2_O_4_@SiO_2_–SO_3_H serves as an effective catalyst overall, its performance can be significantly impacted by operational parameters and experimental design. The substantial yield achieved by Series 1 suggests that optimal conditions were maintained throughout the experiment, whereas Series 2's inability to improve beyond a certain threshold point towards possible inefficiencies or limitations inherent in its setup.

This analysis underscores the importance of conducting thorough hot filtration tests when evaluating catalytic systems. The observed discrepancies between Series 1 and Series 2 not only highlight the efficacy of AlFe_2_O_4_@SiO_2_–SO_3_H as a catalyst but also prompt further investigation into optimizing reaction conditions and understanding factors leading to catalyst deactivation or reduced performance over time. Such insights are invaluable for advancing synthetic methodologies and enhancing catalytic processes in organic chemistry.

Next, to validate the gram scale applicability of the developed protocol, we carried out the reaction of benzo[*d*]oxazole (0.01 mol, 1.19 g) with bromobenzene (0.01 mol, 1.57 g) and S_8_ (0.02 mol, 5.12 g) as a sulfur source under the set reaction conditions and successfully obtained the desired product (product 4a) (97% yield, 2.20 g).

Table 5 provides a comparison of the efficiency of different methods for synthesizing 2-(phenylthio)benzo[*d*]thiazole (product 4a). The efficiency is evaluated based on the time required for the reaction and the yield of the product. The table shows that the method developed in this study, using AlFe_2_O_4_@SiO_2_–SO_3_H as a catalyst, is the most efficient. It requires only 3 hours to complete and achieves a yield of 95%. Other methods reported in the literature typically require longer reaction times and lower yields.

**Table 5:** Comparison of the efficiency of this method with reported methods for the synthesis of 2-thioarylbenzoazoles (product 4a) as the model reaction

Entry	Catalyst	Condition	Time (h)	Yield (%)	Ref.
1	AgO_2_CCF_3_/Cu(OAc)_2_	DMF, 120 °C	12	76	39
2	CuFe_2_O_4_ NPs	ChCl-urea, KOAc, 100 °C	8	97	36
3	Fe_3_O_4_ NPs	K_2_CO_3_, DMF, 120 °C	12	68	37
4	CuI/Bipy	Na_2_CO_3_, DMF 140 °C	24	90	38
5	(IPr)CuI	DMF, K_2_CO_3_, 140 °C	3	81	40
**6**	**AlFe**_**2**_ **O**_**4**_ **@SiO**_**2**_ **–SO**_**3**_ **H**	**ZnCl**_**2**_ **+ urea, 60 °C**	**3**	**95**	**This method**

## Experimental

All chemicals, including reagents and solvents, were carefully selected from reputable suppliers such as Sigma and Merck for their specific roles in the synthesis process. The samples' infrared spectra (IR) were recorded in KBr disks using a NICOLET impact 410 spectrometer. ^1^HNMR and ^13^CNMR spectra were recorded with a Bruker DRX-400 spectrometer at 400 and 100 MHz, respectively.

### Synthesis of nanocatalysts

#### Synthesis of AlFe_2_O_4_ nanoparticles

AlFe_2_O_4_ was fabricated *via* a co-precipitation chemical process. FeCl_2_·4H_2_O and Al(NO_3_)_3_·9H_2_O were initially dissolved in 100 mL of water, and maintained under a nitrogen atmosphere at 80 °C with a molar ratio of 2 : 1. Following this, 10 mL of 0.2 M NaOH solution was incrementally added over 10 minutes to the agitated mixture, achieving a final pH of 12. After 30 minutes of continuous stirring, the AlFe_2_O_4_ MNPs were magnetically separated, washed multiple times with deionized water, and dried at 75 °C overnight.

#### Synthesis of AlFe_2_O_4_@SiO_2_

The interlayers of SiO_2_ were prepared through a modified Stober method,^41^ a significant innovation in our research that enhances the properties of the AlFe_2_O_4_@SiO_2_ nanocatalyst.

#### Synthesis of AlFe_2_O_4_@SiO_2_–SO_3_H

Functionalization of prepared nanoparticles with –SO_3_H, as an organic acid, was performed by using a suction flask equipped with a constant-pressure dropping funnel and a gas inlet tube for conducting HCl gas over an adsorbing solution (*i.e.*, water), charged with the AlFe_2_O_4_@SiO_2_ (0.04 g) in dry CH_2_Cl_2_ (10 mL). Then, the flask was placed in the ice bath, and chlorosulfonic acid (0.10 mL) was added drop by drop over 30 min at room temperature. HCl gas immediately evolved from the reaction vessel. Stirring was continued until HCl evolution was fetched up. After the addition was completed, the mixture was stirred for 30 min. A powder of AlFe_2_O_4_@SiO_2_–SO_3_H-supported sulfonic acid was obtained. Then, the nanoparticles were precipitated by adding 10 mL methanol to the mixture. After that, using an external magnet, the nanoparticles were separated and alternatively washed with ethanol (10 mL) and distilled water three times and then dried at 70 °C. The prepared nanoparticles were placed in a vacuum desiccator over anhydrous silica gel and then dried at 120 °C for 6 h.

### Preparation of the DES based on zinc chloride/urea

The DES was prepared by mixing zinc chloride (1 mmol) with urea (2 mmol) and stirring the mixture for 1 h at 80 °C in air. This process resulted in the formation of a deep eutectic solvent, which was used without any further purification.

### General procedure for the preparation of 2-thioarylbenzoazoles

The AlFe_2_O_4_@SiO_2_–SO_3_H nanocatalyst (10 mg) was added to a mixture of benzothiazole, benzimidazole or benzoxazole (1 mmol), aryl bromides (1 mmol), and S8 as the sulfur source (2 mmol) in the presence of K_2_CO_3_ (3 mmol) as a base, and ZnCl_2_ + urea (ratio: 1 : 2) as a solvent (5 mL) in a flask, and the reaction was stirred at 60 °C under N_2_ for 3 h. The reaction progress was monitored by TLC using a hexane/ethyl acetate mixture for elution. Following the reaction's completion, the catalyst was removed with a magnet, the reaction mixture was poured into ice-cold water, and the obtained precipitate was filtered and washed with ice-cold water and dried. The products were purified by washing with ethyl acetate.

## Conclusion

This manuscript provides a comprehensive exploration of the innovative AlFe_2_O_4_@SiO_2_–SO_3_H nanocatalyst, specifically designed for the A3 coupling reaction in deep eutectic solvents (DESs) to synthesize 2-thioarylbenzoazoles. This study underscores the unique integration of AlFe_2_O_4_ nanoparticles with a sulfonated phenyl functional group, which leverages the high catalytic activity and stability of AlFe_2_O_4_ with the excellent acidic and ionic properties of chlorosulfonic acid, thereby enhancing the overall efficacy and selectivity of the coupling reaction. The utilization of DES as a reaction medium plays a crucial role. DES offers an environmentally friendly alternative to traditional organic solvents by reducing toxicity and enhancing biodegradability. The DES not only supports the dissolution of reactants and the dispersion of the catalyst but also enhances the reaction kinetics under milder conditions, contributing to energy savings and a smaller carbon footprint. The results demonstrate that the AlFe_2_O_4_@SiO_2_–SO_3_H nanocatalyst efficiently promotes the A3 coupling reaction, yielding high-quality 2-thioarylbenzoazoles with excellent selectivity and yield under optimized conditions. The catalyst also exhibits outstanding reusability with consistent activity over multiple cycles, thereby reducing waste and furthering the goals of sustainable chemical manufacturing.

Overall, this research highlights the potential of integrating advanced nanomaterials with green solvents to develop more sustainable catalytic strategies in organic synthesis. The findings from this study not only contribute to advancing the field of catalysis but also pave the way for future research aimed at expanding the applicability of this novel catalytic system to other types of organic transformations, thereby enhancing the scope of green chemistry practices in the industry. This innovative approach represents a significant step forward in our quest to develop more sustainable and environmentally friendly methods for chemical synthesis, particularly in the production of pharmacologically relevant compounds.

## Conflicts of interest

The authors declare no conflict of interest.

## Supplementary Material

NA-007-D5NA00247H-s001

## Data Availability

The datasets generated and analyzed during the current study are available from the corresponding authors on reasonable request.
