# Supplementary material for: An AlFe2O4@SiO2–SO3H innovative nanocatalyst: a sustainable approach for the A3 coupling reaction in a DES for 2-thioarylbenzoazoles
Source: Nanoscale Adv. 2025 May 8;7(13):4039–55. doi: 10.1039/d5na00247h (PMC12101128; doi:10.1039/d5na00247h)
Supplement: NA-007-D5NA00247H-s001 [file NA-007-D5NA00247H-s001.pdf]

Supplementary Information (SI) for Nanoscale Advances.
This journal is © The Royal Society of Chemistry 2025

$AlFe_2O_4$@Ph-SO_3H Innovative Nanocatalyst: A Sustainable Approach for the A3 Coupling Reaction in DES for 2-Thioarylbenzoazoles

Ahmad Sajjadi,[*1] Suranjana V. Mayani,[2] Suhas Ballal,[3] Shaker Al-Hasnaawei,[4(a,b)]

Abhayveer Singh,[5] Kattela Chennakesavulu,[6] Kamal Kant Joshi[7(a, b)]

1*

Young Researchers and Elite Club, Tehran Branch, Islamic Azad University, Tehran, Iran.
sajjadiahmmad@gmail.com

2

Marwadi University Research Center, Department of Chemistry, Faculty of Science

Marwadi University, Rajkot, Gujarat

suranjana.mayani@marwadieducation.edu.in

3

Department of Chemistry and Biochemistry, School of Sciences, JAIN (Deemed to be University), Bangalore, Karnataka, India.

b.suhas@jainuniversity.ac.in

4

[a] College of pharmacy, the Islamic University, Najaf, Iraq

[b] Department of medical analysis, Medical laboratory technique college, the Islamic University of Al Diwaniyah, Al Diwaniyah, Iraq.

shakeralhasnawi@iunajaf.edu.iq

5

Centre for Research Impact & Outcome, Chitkara University Institute of Engineering and Technology, Chitkara University, Rajpura, 140401, Punjab, India.

abhayveer_singh@outlook.com

6

Department of Chemistry, Sathyabama Institute of Science and Technology, Chennai, Tamil Nadu, India.

chennakesavulureddy.chemistry@sathyabama.ac.in

7

[a] Department of Allied Science, Graphic Era Hill University, Dehradun, India.

[b] Adjunct Professor, Graphic Era Deemed to be University, Dehradun, Uttarakhand, India.

kkjoshi@gehu.ac.in

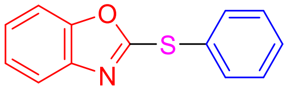

2-(phenylthio)benzo[*d*]oxazole: Colorless oil, ^{1}H NMR (400 MHz, $CDCl_3$) δ 7.76 (d, J = 7.8 Hz, 2H), 7.66 (d, J = 8.0 Hz, 2H), 7.52 (d, J = 8.6 Hz, 2H), 7.47 – 7.42 (m, 3H).; ^{13}C NMR (101 MHz, $CDCl_3$) δ 154.9, 152.0, 141.3, 134.8, 126.8, 126.5, 125.7, 124.3, 123.6, 122.7, 121.0, 109.3.

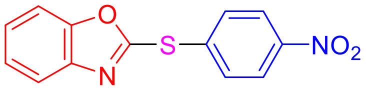

2-(phenylthio)benzo[*d*]oxazole: mp: 93-95 °C, ^{1}H NMR (400 MHz, $CDCl_3$) δ 7.91 (d, J = 7.7 Hz, 2H), 7.64 (dd, J = 9.1, 3.5 Hz, 4H), 7.38 (d, J = 8.6 Hz, 2H).; ^{13}C NMR (101 MHz, $CDCl_3$) δ 154.0, 147.6, 143.2, 136.9, 131.1, 124.7, 123.2, 122.0, 121.3, 120.8, 119.8, 119.1, 101.5.

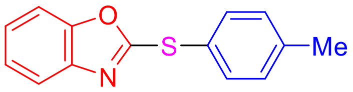

2-(p-tolylthio)benzo[*d*]oxazole: Colorless oil, ^{1}H NMR (400 MHz, $CDCl_3$) δ 7.83 (d, J = 7.7 Hz, 2H), 7.65-7.63 (m, 2H), 7.46 (d, J = 8.6 Hz, 2H), 7.34 (d, J = 9.0 Hz, 2H), 2.39 (s, 3H); ^{13}C NMR (101 MHz, $CDCl_3$) δ 155.0, 153.1, 142.8, 137.6, 127.5, 126.0, 124.3, 123.7, 122.6, 121.0, 119.8, 108.5, 20.1.

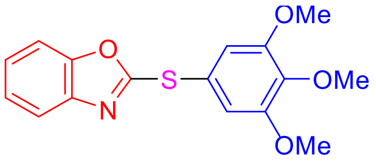

2-((3,4,5-trimethoxyphenyl)thio)benzo[*d*]oxazole: mp: 129-131 ºC, ^{1}H NMR (400 MHz, $CDCl_3$) δ 7.71 (d, J = 8.0 Hz, 2H), 7.43 (d, J = 8.6 Hz, 2H), 7.12 (s, 2H), 3.85 (s, 6H), 3.71 (s, 3H); ^{13}C NMR (101 MHz, $CDCl_3$) δ 154.3, 151.9, 142.7, 134.1, 130.7, 129.8, 128.4, 127.4, 119.7, 112.6, 61.9, 55.4.

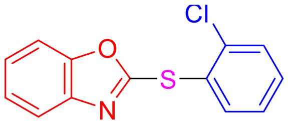

2-((2-chlorophenyl)thio)benzo[*d*]oxazole: mp: 46-48 ºC, ^{1}H NMR (400 MHz, $CDCl_3$) δ 7.81 (d, J = 7.8 Hz, 1H), 7.75 (d, J = 7.7 Hz, 2H), 7.68 (d, J = 8.0 Hz, 2H), 7.61 (d, J = 7.6 Hz, 1H), 7.33 – 7.30 (m, 1H), 7.11 (t, J = 7.7 Hz, 1H); ^{13}C NMR (101 MHz, $CDCl_3$) δ 145.1, 152.6, 143.2, 134.7, 132.8, 131.5, 129.0, 128.6, 126.4, 119.8, 112.5.

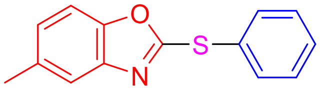

5-methyl-2-(phenylthio)benzo[*d*]oxazole: mp: 48-50 ºC, ^{1}H NMR (400 MHz, $CDCl_3$) δ 7.88 (d, J = 7.4 Hz, 1H), 7.71 (s, 1H), 7.69 (d, J = 7.6 Hz, 1H), 7.54 (d, J = 8.6 Hz, 2H), 7.30 (d, J = 7.7 Hz, 2H), 7.16 – 7.09 (m, 1H), 2.61 (s, 3H).; ^{13}C NMR (101 MHz, $CDCl_3$) δ 153.2, 138.9, 131.5, 126.7, 125.1, 123.6, 120.1, 100.7, 21.0.

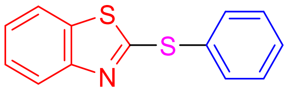

2-(phenylthio)benzo[*d*]thiazole: mp: 33-35 ºC, ^{1}H NMR (400 MHz, $CDCl_3$) δ 8.03 (d, J = 8.0 Hz, 2H), 7.91 (d, J = 7.7 Hz, 2H), 7.37 (t, J = 8.6 Hz, 2H), 7.20 (d, J = 7.8 Hz, 2H), 7.08-7.04 (m, 1H); ^{13}C NMR (126 MHz, $CDCl_3$) δ 163.2, 156.7, 138.0, 133.9, 127.6, 126.5, 124.3, 123.7, 122.1, 121.0, 120.5.

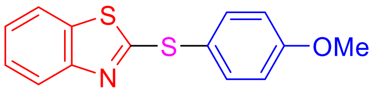

2-((4-methoxyphenyl)thio)benzo[*d*]thiazole: mp: 55-57 ºC, $_{1}$H NMR (400 MHz, $CDCl_3$) δ 7.34 (d, J = 7.4 Hz, 1H), 8.09 (d, J = 7.5 Hz, 1H), 7.54-7.48 (m, 2H), 7.37–7.33 (m, 2H), 7.04 (d, J = 7.0 Hz, 2H), , 3.83

(s, 3H); ^{13}C NMR (101 MHz, $CDCl_3$) δ 163.8, 156.0, 152.1, 138.7, 128.9, 127.4, 126.1, 125.7, 124.3, 113.1, 54.9.

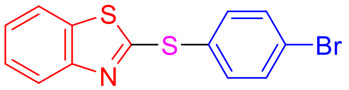

2-((4-bromophenyl)thio)benzo[*d*]thiazole: mp: 50-52 °C, ^{1}H NMR (400 MHz, $CDCl_3$) δ 8.06 (d, J = 7.7 Hz, 1H), 7.81 (d, J = 7.6 Hz, 1H), 7.53 (d, J = 8.0 Hz, 2H), 7.38-7.30 (m, 4H); ^{13}C NMR (101 MHz, $CDCl_3$) δ 167.3, 156.1, 134.7, 129.8, 128.4, 127.1, 124.9, 121.5, 120.3..

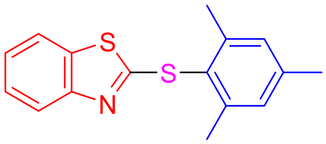

2-(mesitylthio)benzo[*d*]thiazole: Colorless oil, ^{1}H NMR (400 MHz, $CDCl_3$) δ 8.11 (d, J = 8.0 Hz, 1H), 8.01 (d, J = 8.4 Hz, 1H), 7.63-7.55 (m, 2H), 7.01 (s, 2H), 2.42 (s, 6H), 2.18 (s 3H); ^{13}C NMR (101 MHz, $CDCl_3$) δ 163.1, 152.8, 138.7, 135.9, 131.0, 124.7, 123.1, 122.6, 121.0, 120.3, 22.1 19.8..

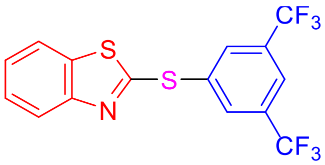

2-((3,5-bis(trifluoromethyl)phenyl)thio)benzo[*d*]thiazole: Colorless oil, ^{1}H NMR (400 MHz, $CDCl_3$) δ 8.21 (d, J = 7.4 Hz, 1H), 8.07 (dd, J = 8.9, 2.3 Hz, 1H), 7.73 (s, 1H), 7.46-7.58 (m, 2H), 7.43 (s, 2H); ^{13}C NMR (101 MHz, $CDCl_3$) δ 166.5, 154.8, 137.4, 129.8, 128.1, 127.3, 126.0, 125.3, 124.7, 123.1, 120.7.

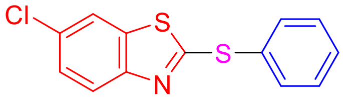

6-chloro-2-(phenylthio)benzo[*d*]thiazole: mp: 69-71 °C, ^{1}H NMR (400 MHz, $CDCl_3$) δ 8.10 (s, 1H), 7.83 (d, J = 7.8 Hz, 1H), 7.64 (d, J = 8.6 Hz, 1H), 7.33 (d, J = 8.0 Hz, 2H), 7.25-7.19 (m, 3H); ^{13}C NMR (101 MHz, $CDCl_3$) δ 163.2, 153.1, 138.6, 126.5, 125.3, 124.6, 123.0, 121.8.

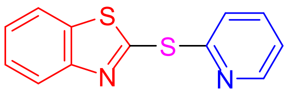

2-(pyridin-2-ylthio)benzo[*d*]thiazole: mp: 66-68 ºC, ^{1}H NMR (400 MHz, CDCl3) δ 8.41 (d, J = 8.0 Hz, 1H), 8.23 (d, J = 7.5 Hz, 1H), 8.03 (d, J = 8.1 Hz, 1H), 7.46 (t, J = 8.1 Hz, 1H), 7.21–7.18 (m, 2H), 7.04-7.00 (m, 2H); 13C NMR (101 MHz, CDCl3) δ 161.0, 154.3, 153.9, 149.8, 134.9, 133.6, 125.4, 123.6, 121.4.

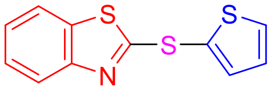

2-(thiophen-2-ylthio)benzo[*d*]thiazole: Colorless oil, ^{1}H NMR (400 MHz, $CDCl_3$) δ 7.83 (d, J = 7.6 Hz, 1H), 7.78 (d, J = 8.0 Hz, 1H), 7.62-7.57 (m, 3H), 7.51 (d, J = 8.4 Hz, 1H), 7.16 (d, J = 7.6 Hz, 1H); ^{13}C NMR (101 MHz, $CDCl_3$) δ 164.1, 155.8, 135.4, 134.2, 125.7, 125.0, 124.8, 124.2, 122.8, 121.1.

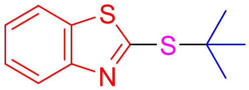

2-(tert-butylthio)benzo[*d*]thiazole: Colorless oil, ^{1}H NMR (400 MHz, $CDCl_3$) δ 7.89 (d, J = 8.4 Hz, 1H), 7.76 (d, J = 7.9 Hz, 1H), 7.43-7.37 (m, 2H); ^{13}C NMR (101 MHz, $CDCl_3$) δ 164.1, 155.8, 132.9, 126.3, 125.1, 124.7, 122.0, 45.6, 30.7.

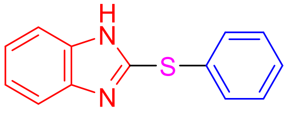

2-(phenylthio)-1*H*-benzo[*d*]imidazole : mp: 202-204 ºC, ^{1}H NMR (400 MHz, CDCl3) δ 9.76 (s, 1H), 7.98 (d, J = 8.4 Hz, 2H), 7.43 (d, J = 8.0 Hz, 2H), 7.36 (t, J = 7.6 Hz, 2H), 7.27-7.20 (m, 1H), 7.04-6.97 (m, 2H); ^{13}C NMR (101 MHz, CDCl3) δ 141.9, 139.5, 136.7, 129.8, 128.1, 126.7, 116.5.

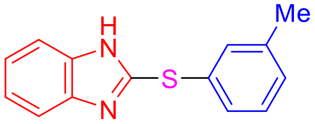

2-(m-tolylthio)-1*H*-benzo[*d*]imidazole: Colorless oil, ^{1}H NMR (400 MHz, $CDCl_3$) δ 9.80 (s, 1H), 7.83 (d, J = 8.6 Hz, 2H), 7.66 (dd, J = 8.8, 3.1 Hz, 1H), 7.48-7.43 (m, 3H), 7.32 (s, 1H), 7.11 (dd, J = 9.2, 1.4 Hz, 1H), 2.46 (s, 3H); ^{13}C NMR (101 MHz, $CDCl_3$) δ 139.0, 131.6, 123.1, 122.6, 121.9, 120.5, 120.3, 119.8, 114.3, 114.0, 21.5.

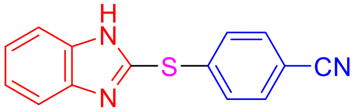

4-((1*H*-benzo[*d*]imidazol-2-yl)thio)benzonitrile: mp: 178-180 °C, ^{1}H NMR (400 MHz, CDCl3) δ 9.82 (s, 1H), 7.75 (d, J = 7.4 Hz, 2H), 7.62 (d, J = 7.7 Hz, 2H), 7.41 (d, J = 8.6 Hz, 2H), 7.35-7.29 (m, 2H); ^{13}C NMR (101 MHz, CDCl3) δ 140.7, 138.5, 137.2, 133.0, 131.4, 121.3, 119.5, 117.0.

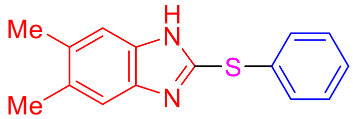

5,6-dimethyl-2-(phenylthio)-1*H*-benzo[*d*]imidazole: mp: 167-169 °C, ^{1}H NMR (400 MHz, CDCl3) δ 9.79 (s, 1H), 7.85-7.81 (m, 3H), 7.63 (dd, J = 8.7, 3.6 Hz, 2H), 7.50 (s, 2H), 2.34 (s, 6H); ^{13}C NMR (101 MHz, CDCl3) δ 138.7, 130.6, 130.3, 129.8, 129.2, 128.0, 127.4, 120.4, 120.0, 116.9.

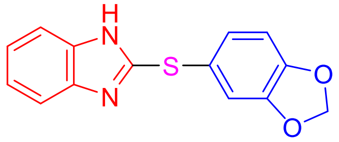

2-(benzo[d][1,3]dioxol-5-ylthio)-1H-benzo[d]imidazole: mp: 183-185 °C, ^{1}H NMR (400 MHz, CDCl3) δ 9.74 (s,1H), 7.73 (d, J = 8.4 Hz, 2H), 7.66-7.61 (m, 2H), 7.37-7.32 (m, 1H), 7.16 (s, 1H), 7.09 (d, J = 8.0 Hz, 1H), 6.07 (s, 2H); ^{13}C NMR (101 MHz, CDCl3) δ 150.1, 138.7, 131.7, 129.8, 124.8, 123.1, 122.0, 121.5, 120.7, 116.7, 101.3.

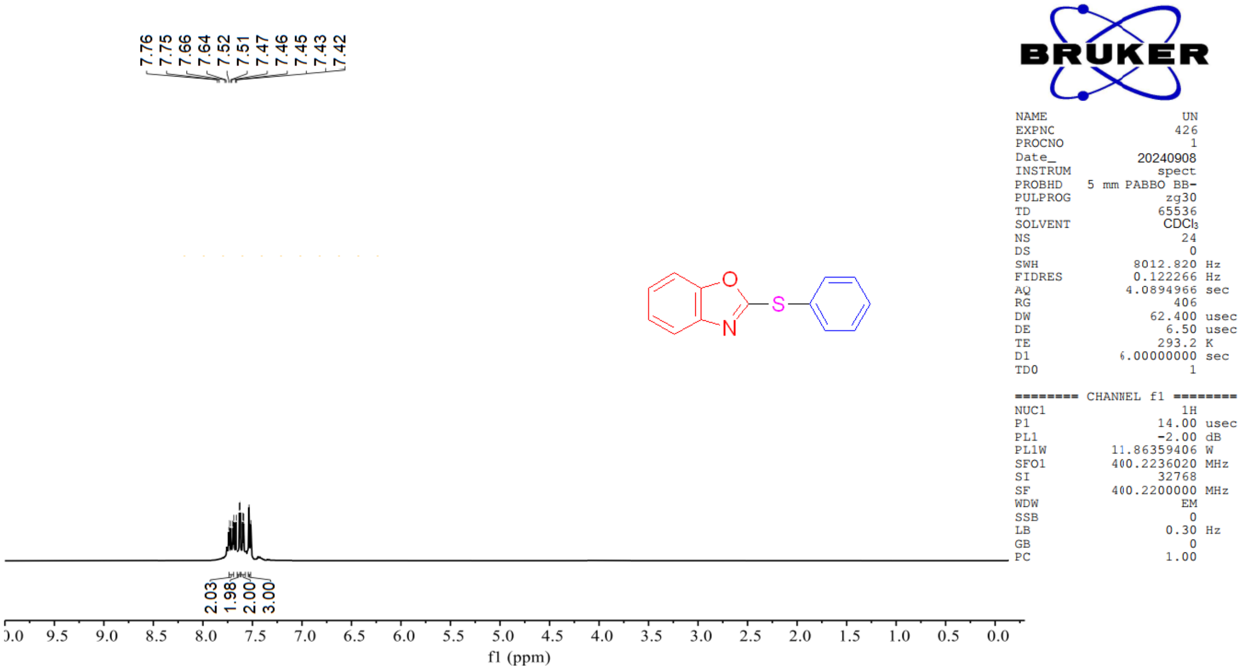

7.76
7.75
7.66
7.64
7.52
7.51
7.47
7.46
7.45
7.43
7.42
O
N
S
2.03
1.98
2.00
3.00
f1 (ppm)
BRUKER
NAME UN
EXPNO 426
PROCNO 1
Date_ 20240908
INSTRUM spect
PROBHD 5 mm PABBO BB-
PULPROG zg30
TD 65536
SOLVENT CDCl3
NS 24
DS 0
SWH 8012.820 Hz
FIDRES 0.122266 Hz
AQ 4.0894966 sec
RG 406
DW 62.400 usec
DE 6.50 usec
TE 293.2 K
D1 6.00000000 sec
TD0 1
======== CHANNEL f1 ========
NUC1 1H
P1 14.00 usec
PL1 -2.00 dB
PL1W 11.86359406 W
SFO1 400.2236020 MHz
SI 32768
SF 400.2200000 MHz
WDW EM
SSB 0
LB 0.30 Hz
GB 0
PC 1.00

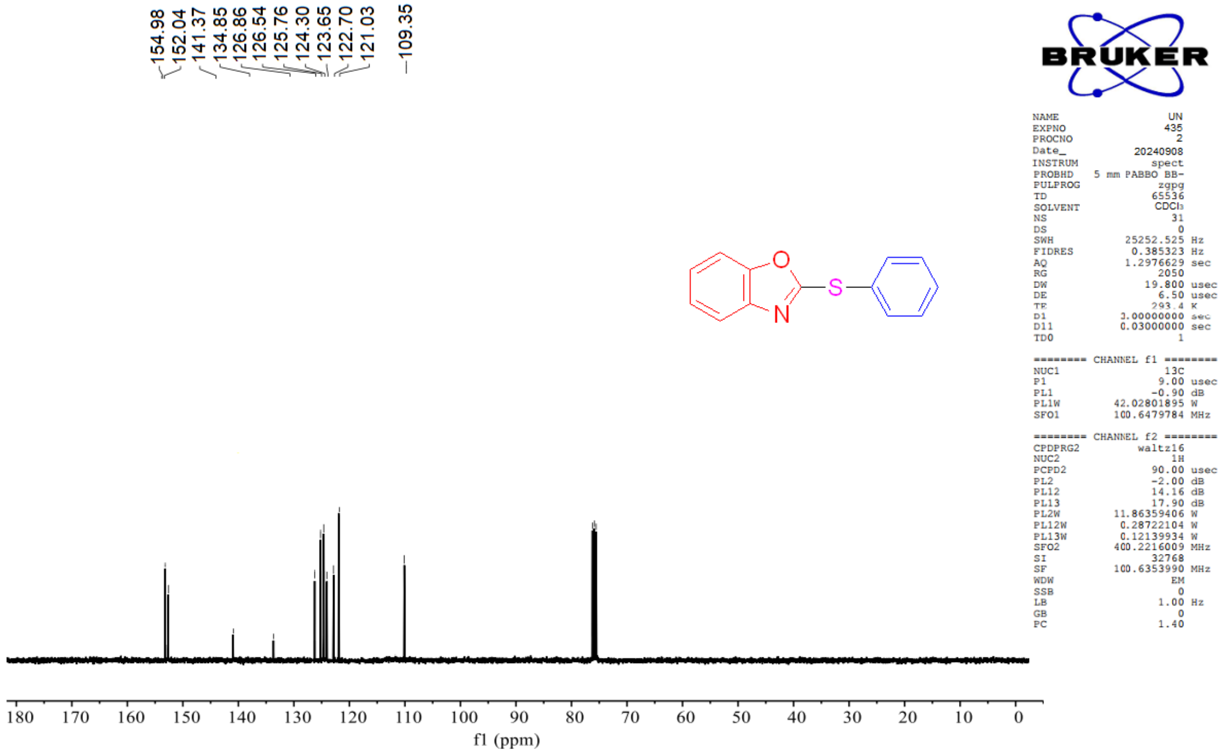

154.98
152.04
141.37
134.85
126.86
126.54
125.76
124.30
123.65
122.70
121.03
109.35
O
N
S
f1 (ppm)
BRUKER
NAME UN
EXPNO 435
PROCNO 2
Date_ 20240908
INSTRUM spect
PROBHD 5 mm PABBO BB-
PULPROG zgpg
TD 65536
SOLVENT CDCl3
NS 31
DS 0
SWH 25252.525 Hz
FIDRES 0.385323 Hz
AQ 1.2976629 sec
RG 2050
DW 19.800 usec
DE 6.50 usec
TE 293.4 K
D1 3.00000000 sec
D11 0.03000000 sec
TD0 1
======== CHANNEL f1 ========
NUC1 13C
P1 9.00 usec
PL1 -0.90 dB
PL1W 42.02801895 W
SFO1 100.6479784 MHz
======== CHANNEL f2 ========
CPDPRG2 waltz16
NUC2 1H
PCPD2 90.00 usec
PL2 -2.00 dB
PL12 14.16 dB
PL13 17.90 dB
PL2W 11.86359406 W
PL12W 0.28722104 W
PL13W 0.12139934 W
SFO2 400.2216009 MHz
SI 32768
SF 100.6353990 MHz
WDW EM
SSB 0
LB 1.00 Hz
GB 0
PC 1.40

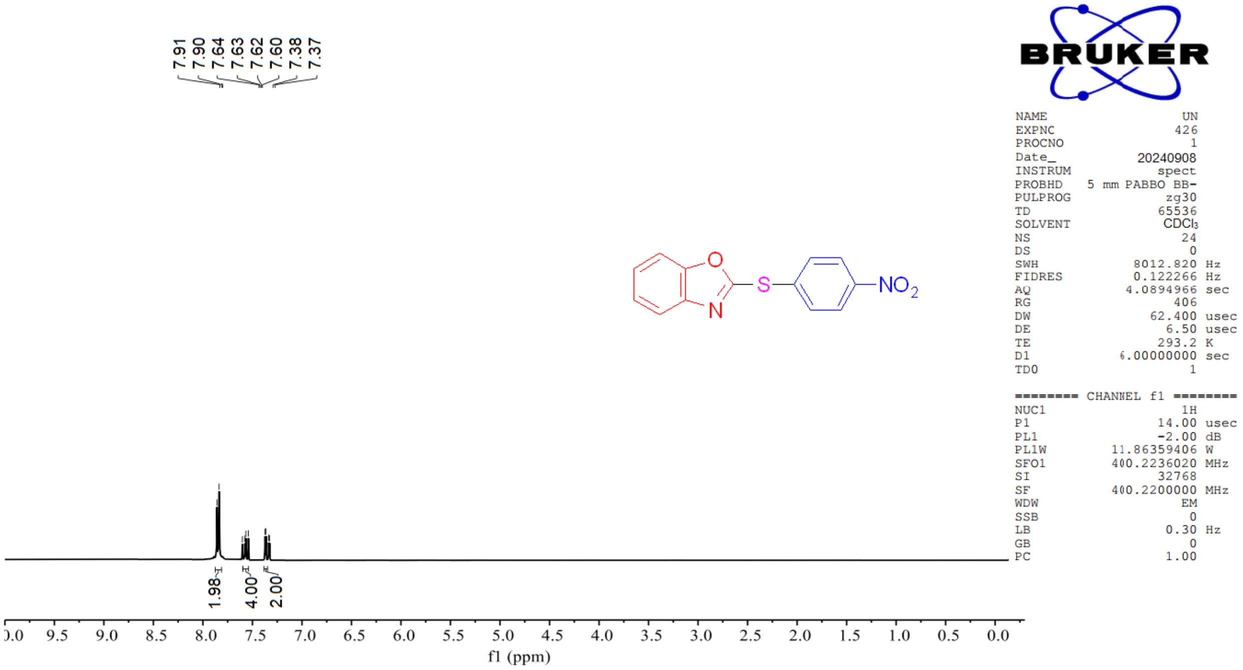

7.91
7.90
7.64
7.63
7.62
7.60
7.38
7.37
BRUKER
NAME UN
EXPNC 426
PROCNO 1
Date_ 20240908
INSTRUM spect
PROBHD 5 mm PABBO BB-
PULPROG zg30
TD 65536
SOLVENT CDCl3
NS 24
DS 0
SWH 8012.820 Hz
FIDRES 0.122266 Hz
AQ 4.0894966 sec
RG 406
DW 62.400 usec
DE 6.50 usec
TE 293.2 K
D1 6.00000000 sec
TD0 1
======== CHANNEL f1 ========
NUC1 1H
P1 14.00 usec
PL1 -2.00 dB
PL1W 11.86359406 W
SFO1 400.2236020 MHz
SI 32768
SF 400.2200000 MHz
WDW EM
SSB 0
LB 0.30 Hz
GB 0
PC 1.00
O
S
N
NO2
1.98
4.00
2.00
9.5 9.0 8.5 8.0 7.5 7.0 6.5 6.0 5.5 5.0 4.5 4.0 3.5 3.0 2.5 2.0 1.5 1.0 0.5 0.0
f1 (ppm)

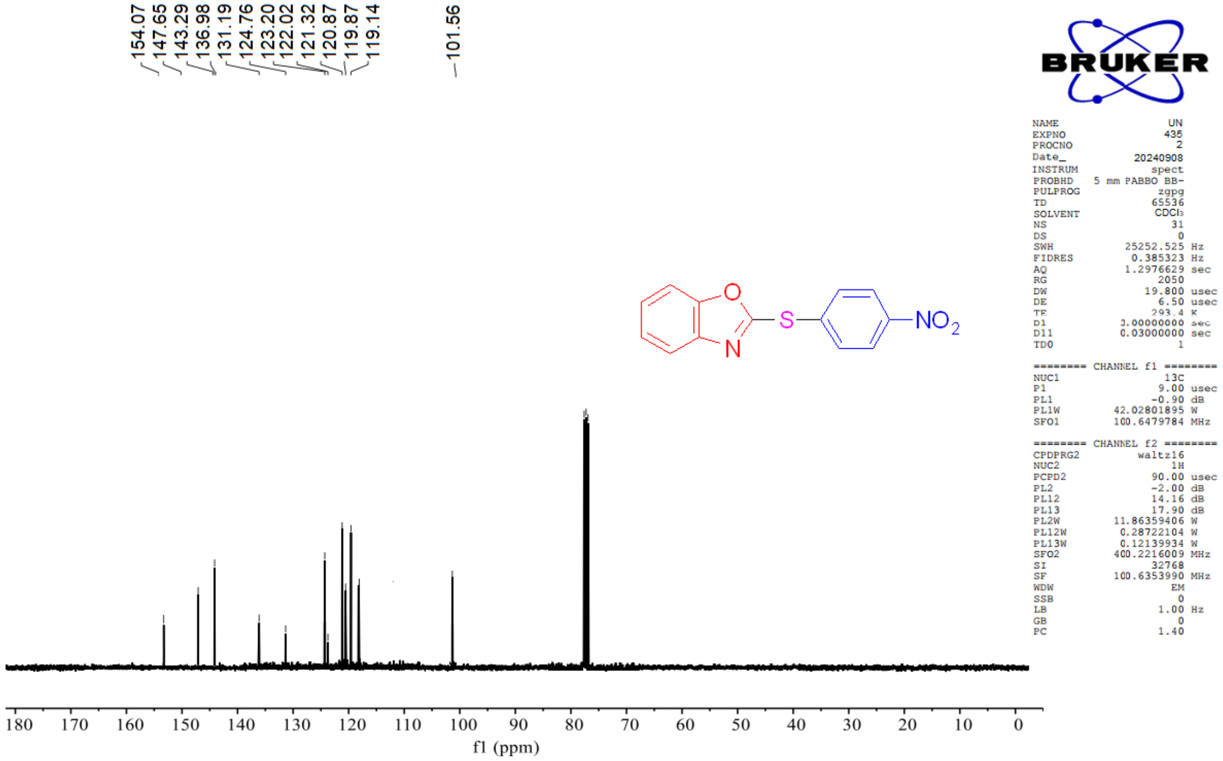

154.07
147.65
143.29
136.98
131.19
124.76
123.20
122.02
121.32
120.87
119.87
119.14
101.56
BRUKER
NAME UN
EXPNO 435
PROCNO 2
Date_ 20240908
INSTRUM spect
PROBHD 5 mm PABBO BB-
PULPROG zgpg
TD 65536
SOLVENT CDCl3
NS 31
DS 0
SWH 25252.525 Hz
FIDRES 0.385323 Hz
AQ 1.2976629 sec
RG 2050
DW 19.800 usec
DE 6.50 usec
TE 293.4 K
D1 3.00000000 sec
D11 0.03000000 sec
TD0 1
======== CHANNEL f1 ========
NUC1 13C
P1 9.00 usec
PL1 -0.90 dB
PL1W 42.02801895 W
SFO1 100.6479784 MHz
======== CHANNEL f2 ========
CPDPRG2 waltz16
NUC2 1H
PCPD2 90.00 usec
PL2 -2.00 dB
PL12 14.16 dB
PL13 17.90 dB
PL2W 11.86359406 W
PL12W 0.28722104 W
PL13W 0.12139934 W
SFO2 400.2216009 MHz
SI 32768
SF 100.6353990 MHz
WDW EM
SSB 0
LB 1.00 Hz
GB 0
PC 1.40
O
S
N
NO2
180 170 160 150 140 130 120 110 100 90 80 70 60 50 40 30 20 10 0
f1 (ppm)

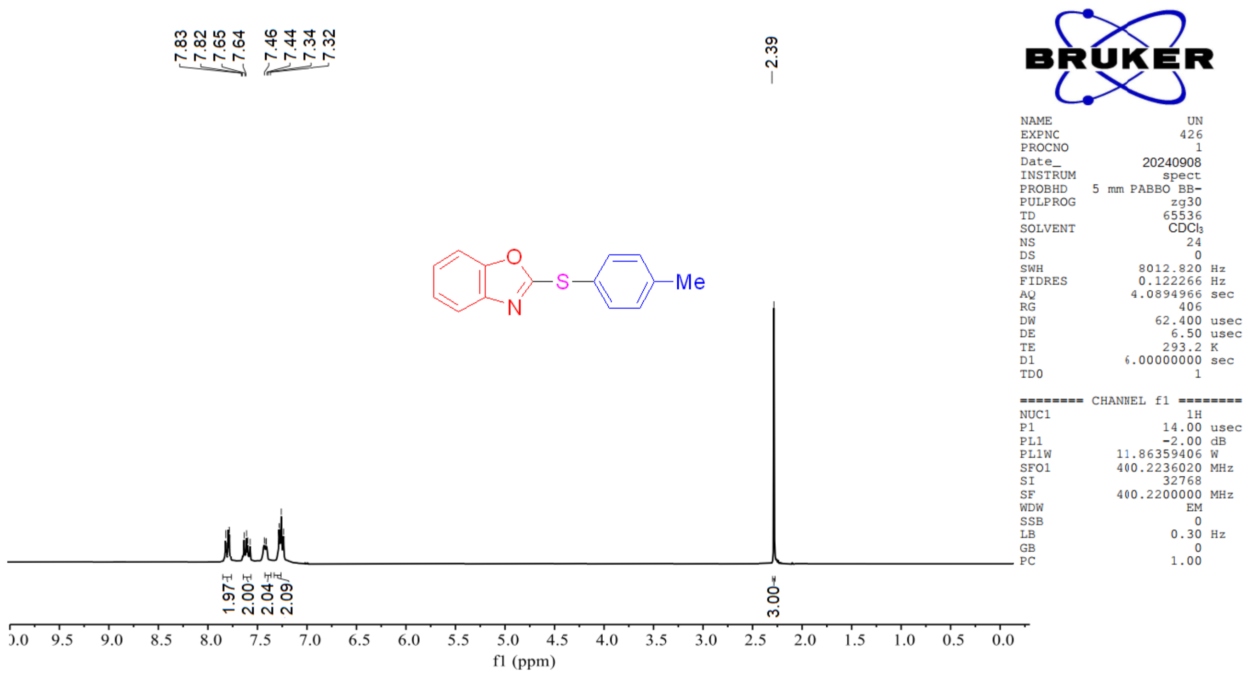

7.83
7.82
7.65
7.64
7.46
7.44
7.34
7.32
2.39
BRUKER
O
N
S
Me
NAME UN
EXPNO 426
PROCNO 1
Date_ 20240908
INSTRUM spect
PROBHD 5 mm PABBO BB-
PULPROG zg30
TD 65536
SOLVENT CDCl3
NS 24
DS 0
SWH 8012.820 Hz
FIDRES 0.122266 Hz
AQ 4.0894966 sec
RG 406
DW 62.400 usec
DE 6.50 usec
TE 293.2 K
D1 6.00000000 sec
TD0 1
======== CHANNEL f1 ========
NUC1 1H
P1 14.00 usec
PL1 -2.00 dB
PL1W 11.86359406 W
SFO1 400.2236020 MHz
SI 32768
SF 400.2200000 MHz
WDW EM
SSB 0
LB 0.30 Hz
GB 0
PC 1.00
1.97
2.00
2.04
2.09
3.00
9.5 9.0 8.5 8.0 7.5 7.0 6.5 6.0 5.5 5.0 4.5 4.0 3.5 3.0 2.5 2.0 1.5 1.0 0.5 0.0
f1 (ppm)

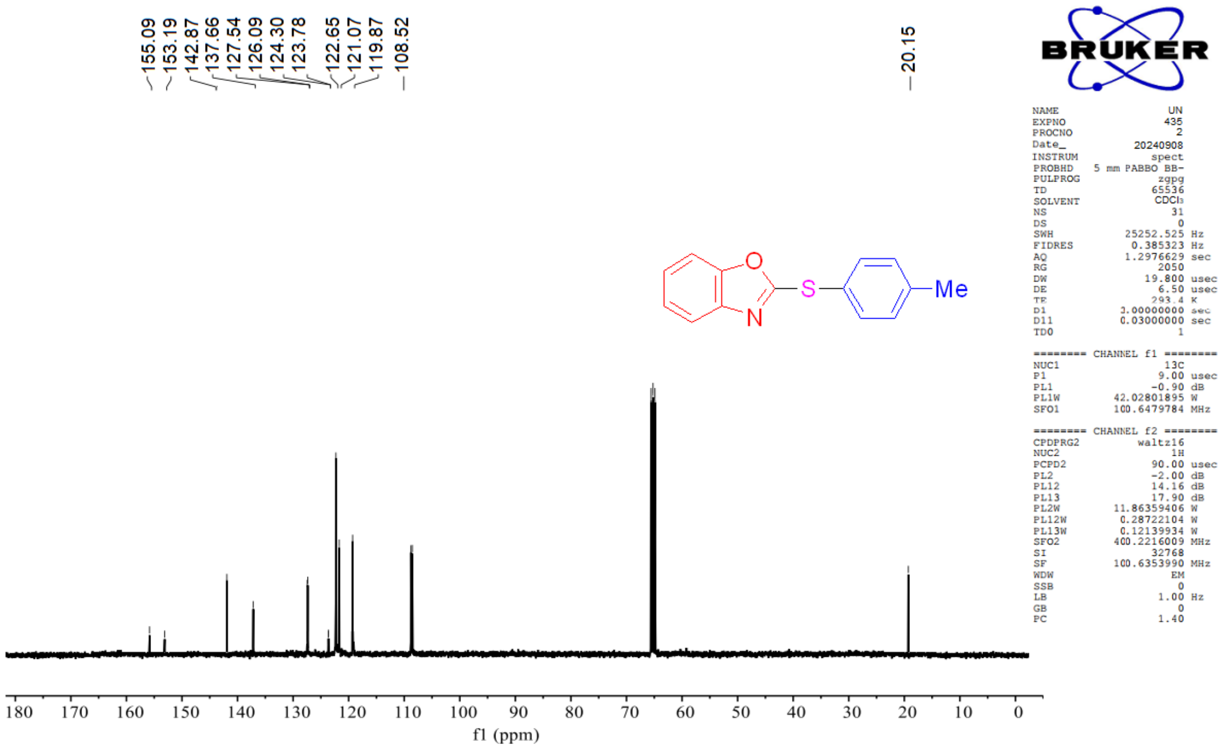

155.09
153.19
142.87
137.66
127.54
126.09
124.30
123.78
122.65
121.07
119.87
108.52
20.15
BRUKER
O
N
S
Me
NAME UN
EXPNO 435
PROCNO 2
Date_ 20240908
INSTRUM spect
PROBHD 5 mm PABBO BB-
PULPROG zgpg
TD 65536
SOLVENT CDCl3
NS 31
DS 0
SWH 25252.525 Hz
FIDRES 0.385323 Hz
AQ 1.2976629 sec
RG 2050
DW 19.800 usec
DE 6.50 usec
TE 293.4 K
D1 3.00000000 sec
D11 0.03000000 sec
TD0 1
======== CHANNEL f1 ========
NUC1 13C
P1 9.00 usec
PL1 -0.90 dB
PL1W 42.02801895 W
SFO1 100.6479784 MHz
======== CHANNEL f2 ========
CPDPRG2 waltz16
NUC2 1H
PCPD2 90.00 usec
PL2 -2.00 dB
PL12 14.16 dB
PL13 17.90 dB
PL2W 11.86359406 W
PL12W 0.28722104 W
PL13W 0.12139934 W
SFO2 400.2216009 MHz
SI 32768
SF 100.6353990 MHz
WDW EM
SSB 0
LB 1.00 Hz
GB 0
PC 1.40
180 170 160 150 140 130 120 110 100 90 80 70 60 50 40 30 20 10 0
f1 (ppm)

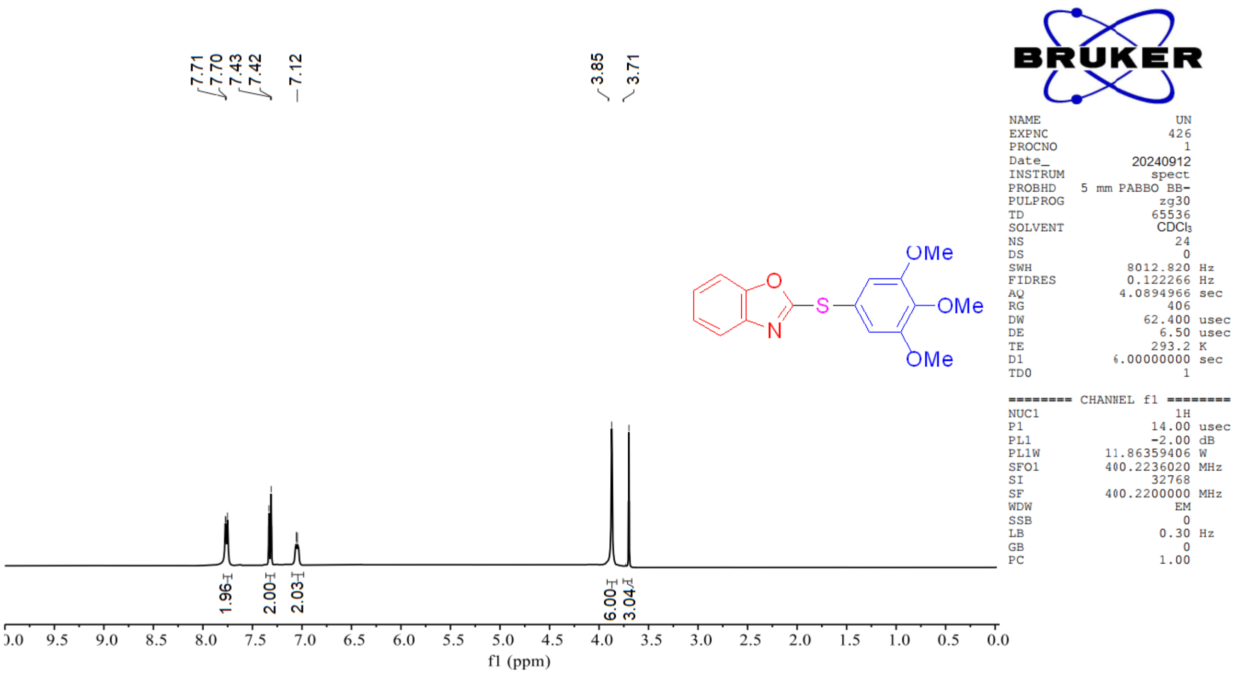

7.71
7.70
7.43
7.42
7.12
3.85
3.71
BRUKER
NAME UN
EXPNO 426
PROCNO 1
Date_ 20240912
INSTRUM spect
PROBHD 5 mm PABBO BB-
PULPROG zg30
TD 65536
SOLVENT CDCl3
NS 24
DS 0
SWH 8012.820 Hz
FIDRES 0.122266 Hz
AQ 4.0894966 sec
RG 406
DW 62.400 usec
DE 6.50 usec
TE 293.2 K
D1 6.00000000 sec
TD0 1
======== CHANNEL f1 ========
NUC1 1H
P1 14.00 usec
PL1 -2.00 dB
PL1W 11.86359406 W
SFO1 400.2236020 MHz
SI 32768
SF 400.2200000 MHz
WDW EM
SSB 0
LB 0.30 Hz
GB 0
PC 1.00
OMe
OMe
OMe
O
N
S
1.96
2.00
2.03
6.00
3.04
f1 (ppm)

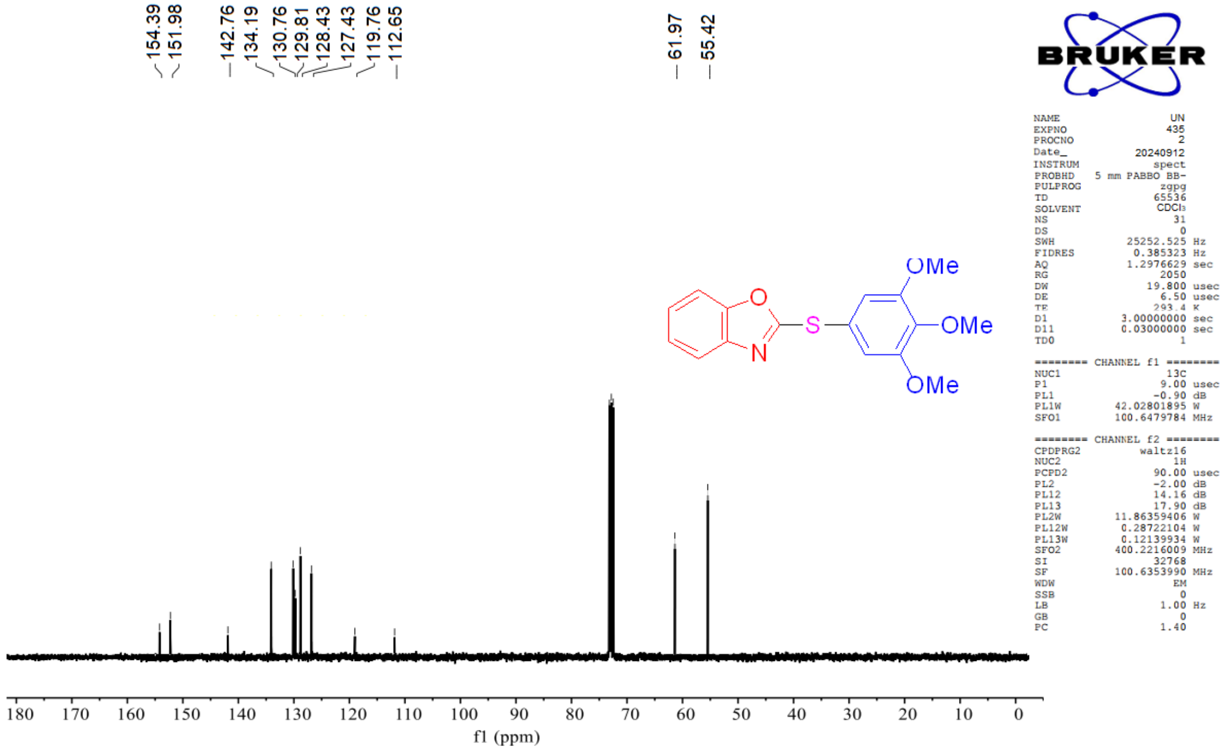

154.39
151.98
142.76
134.19
130.76
129.81
128.43
127.43
119.76
112.65
61.97
55.42
BRUKER
NAME UN
EXPNO 435
PROCNO 2
Date_ 20240912
INSTRUM spect
PROBHD 5 mm PABBO BB-
PULPROG zgpg
TD 65536
SOLVENT CDCl3
NS 31
DS 0
SWH 25252.525 Hz
FIDRES 0.385323 Hz
AQ 1.2976629 sec
RG 2050
DW 19.800 usec
DE 6.50 usec
TE 293.4 K
D1 3.00000000 sec
D11 0.03000000 sec
TD0 1
======== CHANNEL f1 ========
NUC1 13C
P1 9.00 usec
PL1 -0.90 dB
PL1W 42.02801895 W
SFO1 100.6479784 MHz
======== CHANNEL f2 ========
CPDPRG2 waltz16
NUC2 1H
PCPD2 90.00 usec
PL2 -2.00 dB
PL12 14.16 dB
PL13 17.90 dB
PL2W 11.86359406 W
PL12W 0.28722104 W
PL13W 0.12139934 W
SFO2 400.2216009 MHz
SI 32768
SF 100.6353990 MHz
WDW EM
SSB 0
LB 1.00 Hz
GB 0
PC 1.40
OMe
OMe
OMe
O
N
S
f1 (ppm)

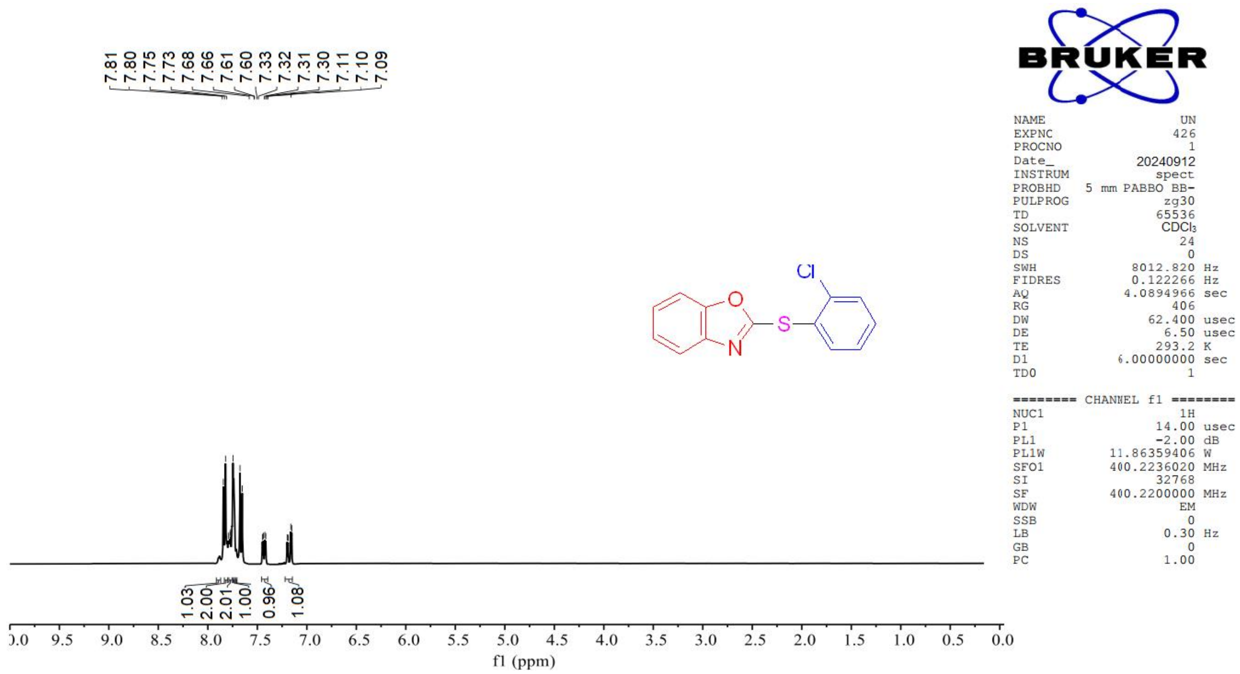
7.81
7.80
7.75
7.73
7.68
7.66
7.61
7.60
7.33
7.32
7.31
7.30
7.11
7.10
7.09
Cl
O
N
S
1.03
2.00
2.01
1.00
0.96
1.08
f1 (ppm)
BRUKER
NAME UN
EXPNO 426
PROCNO 1
Date_ 20240912
INSTRUM spect
PROBHD 5 mm PABBO BB-
PULPROG zg30
TD 65536
SOLVENT CDCl3
NS 24
DS 0
SWH 8012.820 Hz
FIDRES 0.122266 Hz
AQ 4.0894966 sec
RG 406
DW 62.400 usec
DE 6.50 usec
TE 293.2 K
D1 6.00000000 sec
TD0 1
======== CHANNEL f1 ========
NUC1 1H
P1 14.00 usec
PL1 -2.00 dB
PL1W 11.86359406 W
SFO1 400.2236020 MHz
SI 32768
SF 400.2200000 MHz
WDW EM
SSB 0
LB 0.30 Hz
GB 0
PC 1.00

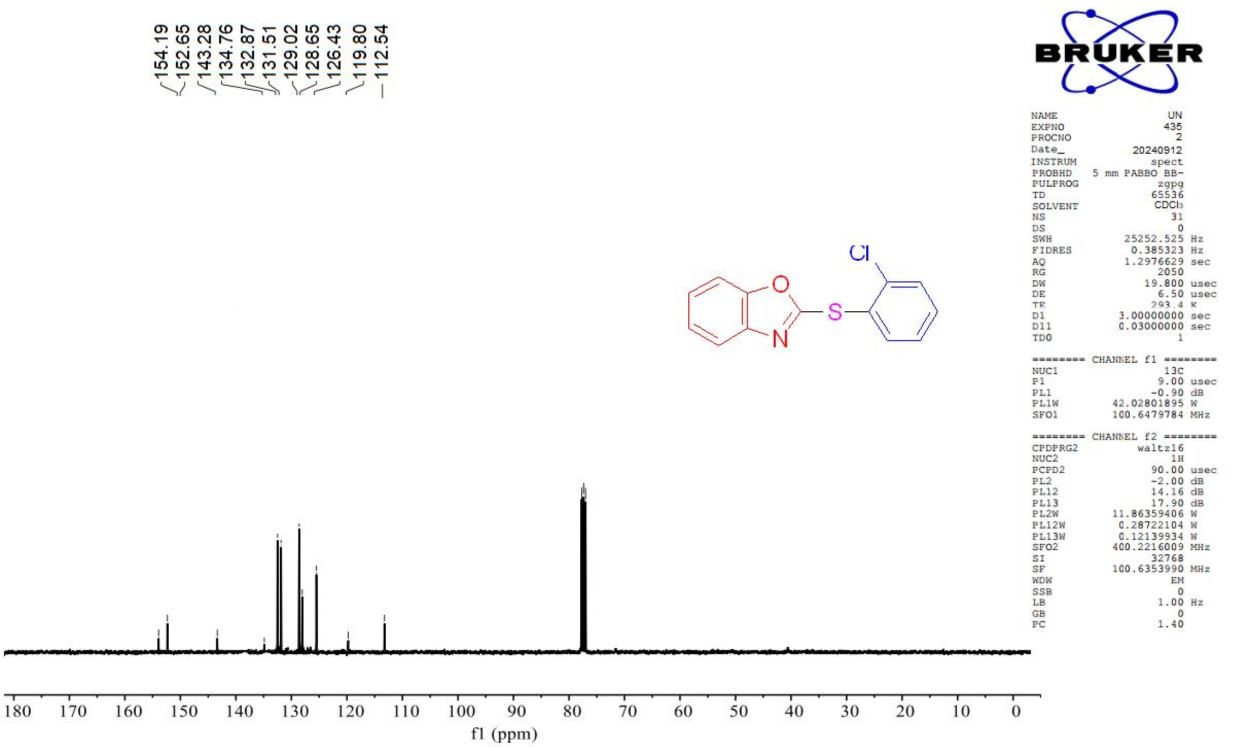
154.19
152.65
143.28
134.76
132.87
131.51
129.02
128.65
126.43
119.80
112.54
Cl
O
N
S
f1 (ppm)
BRUKER
NAME UN
EXPNO 435
PROCNO 2
Date_ 20240912
INSTRUM spect
PROBHD 5 mm PABBO BB-
PULPROG zgpg
TD 65536
SOLVENT CDCl3
NS 31
DS 0
SWH 25252.525 Hz
FIDRES 0.385323 Hz
AQ 1.2976629 sec
RG 2050
DW 19.800 usec
DE 6.50 usec
TE 293.4 K
D1 3.00000000 sec
D11 0.03000000 sec
TD0 1
======== CHANNEL f1 ========
NUC1 13C
P1 9.00 usec
PL1 -0.90 dB
PL1W 42.02801895 W
SFO1 100.6479784 MHz
======== CHANNEL f2 ========
CPDPRG2 waltz16
NUC2 1H
PCPD2 90.00 usec
PL2 -2.00 dB
PL12 14.16 dB
PL13 17.90 dB
PL2W 11.86359406 W
PL12W 0.28722104 W
PL13W 0.12139934 W
SFO2 400.2216009 MHz
SI 32768
SF 100.6353990 MHz
WDW EM
SSB 0
LB 1.00 Hz
GB 0
PC 1.40

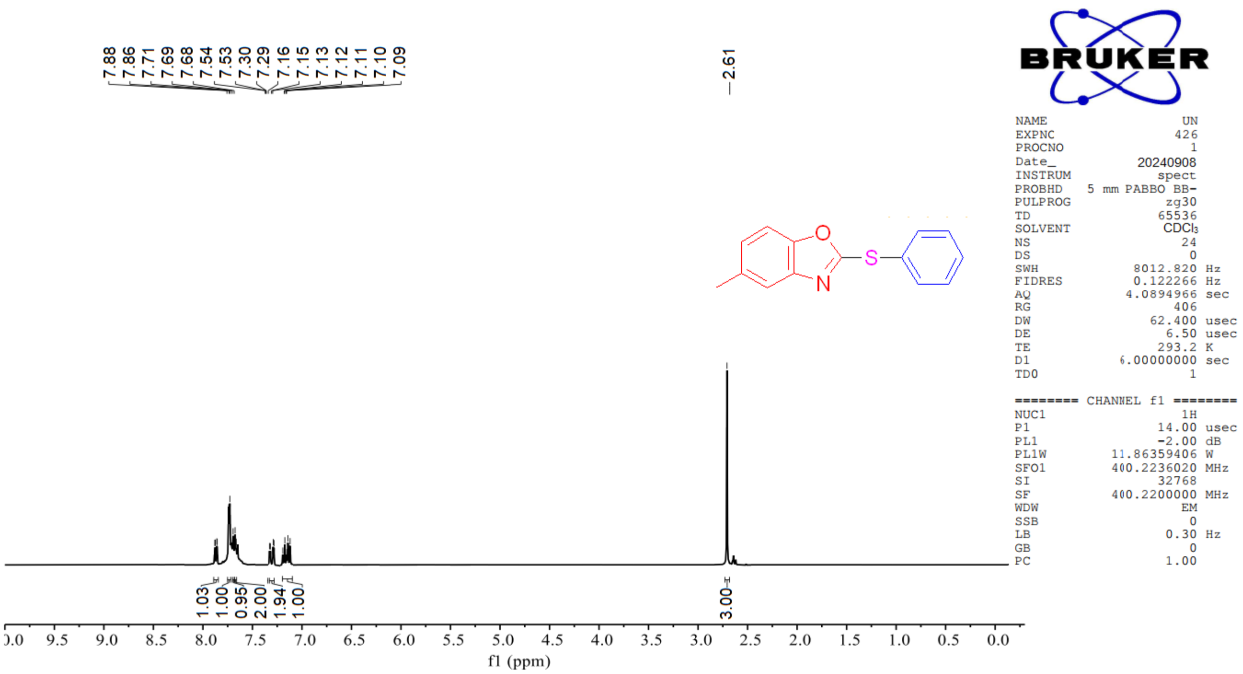
7.88
7.86
7.71
7.69
7.68
7.54
7.53
7.30
7.29
7.16
7.15
7.13
7.12
7.11
7.10
7.09
2.61
O
N
S
BRUKER
NAME UN
EXPNC 426
PROCNO 1
Date_ 20240908
INSTRUM spect
PROBHD 5 mm PABBO BB-
PULPROG zg30
TD 65536
SOLVENT CDCl3
NS 24
DS 0
SWH 8012.820 Hz
FIDRES 0.122266 Hz
AQ 4.0894966 sec
RG 406
DW 62.400 usec
DE 6.50 usec
TE 293.2 K
D1 6.00000000 sec
TD0 1
======== CHANNEL f1 ========
NUC1 1H
P1 14.00 usec
PL1 -2.00 dB
PL1W 11.86359406 W
SFO1 400.2236020 MHz
SI 32768
SF 400.2200000 MHz
WDW EM
SSB 0
LB 0.30 Hz
GB 0
PC 1.00
1.03
1.00
0.95
2.00
1.94
1.00
3.00
10.0 9.5 9.0 8.5 8.0 7.5 7.0 6.5 6.0 5.5 5.0 4.5 4.0 3.5 3.0 2.5 2.0 1.5 1.0 0.5 0.0
f1 (ppm)

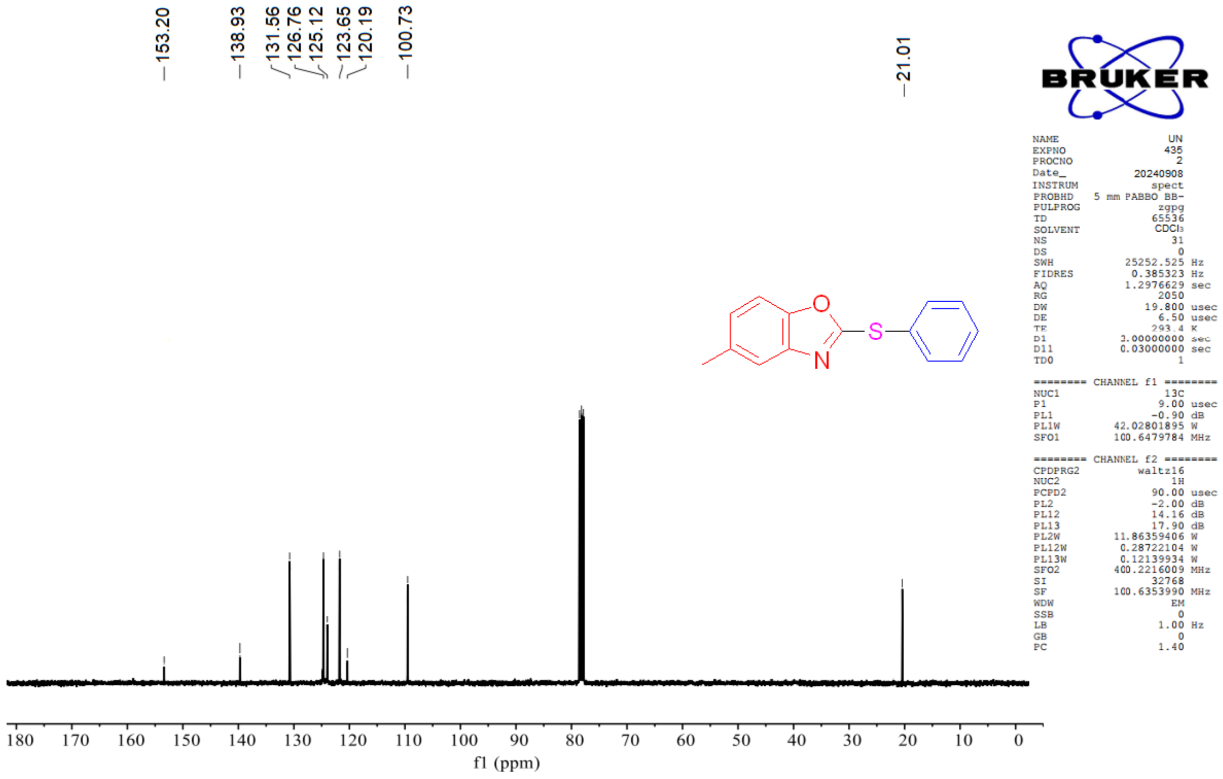
153.20
138.93
131.56
126.76
125.12
123.65
120.19
100.73
21.01
O
N
S
BRUKER
NAME UN
EXPNO 435
PROCNO 2
Date_ 20240908
INSTRUM spect
PROBHD 5 mm PABBO BB-
PULPROG zgpg
TD 65536
SOLVENT CDCl3
NS 31
DS 0
SWH 25252.525 Hz
FIDRES 0.385323 Hz
AQ 1.2976629 sec
RG 2050
DW 19.800 usec
DE 6.50 usec
TE 293.4 K
D1 3.00000000 sec
D11 0.03000000 sec
TD0 1
======== CHANNEL f1 ========
NUC1 13C
P1 9.00 usec
PL1 -0.90 dB
PL1W 42.02801895 W
SFO1 100.6479784 MHz
======== CHANNEL f2 ========
CPDPRG2 waltz16
NUC2 1H
PCPD2 90.00 usec
PL2 -2.00 dB
PL12 14.16 dB
PL13 17.90 dB
PL2W 11.86359406 W
PL12W 0.28722104 W
PL13W 0.12139934 W
SFO2 400.2216009 MHz
SI 32768
SF 100.6353990 MHz
WDW EM
SSB 0
LB 1.00 Hz
GB 0
PC 1.40
180 170 160 150 140 130 120 110 100 90 80 70 60 50 40 30 20 10 0
f1 (ppm)

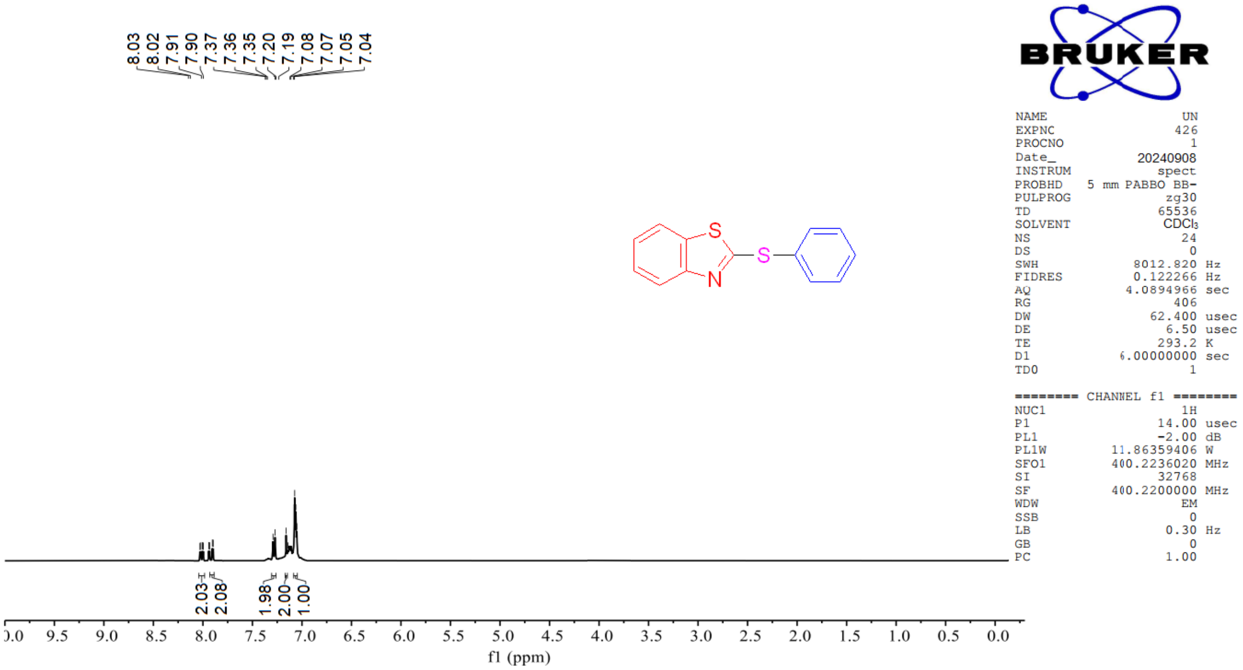
8.03
8.02
7.91
7.90
7.37
7.36
7.35
7.20
7.19
7.08
7.07
7.05
7.04
S
N
S
2.03
2.08
1.98
2.00
1.00
f1 (ppm)
BRUKER
NAME UN
EXPNO 426
PROCNO 1
Date_ 20240908
INSTRUM spect
PROBHD 5 mm PABBO BB-
PULPROG zg30
TD 65536
SOLVENT CDCl3
NS 24
DS 0
SWH 8012.820 Hz
FIDRES 0.122266 Hz
AQ 4.0894966 sec
RG 406
DW 62.400 usec
DE 6.50 usec
TE 293.2 K
D1 6.00000000 sec
TD0 1
======== CHANNEL f1 ========
NUC1 1H
P1 14.00 usec
PL1 -2.00 dB
PL1W 11.86359406 W
SFO1 400.2236020 MHz
SI 32768
SF 400.2200000 MHz
WDW EM
SSB 0
LB 0.30 Hz
GB 0
PC 1.00

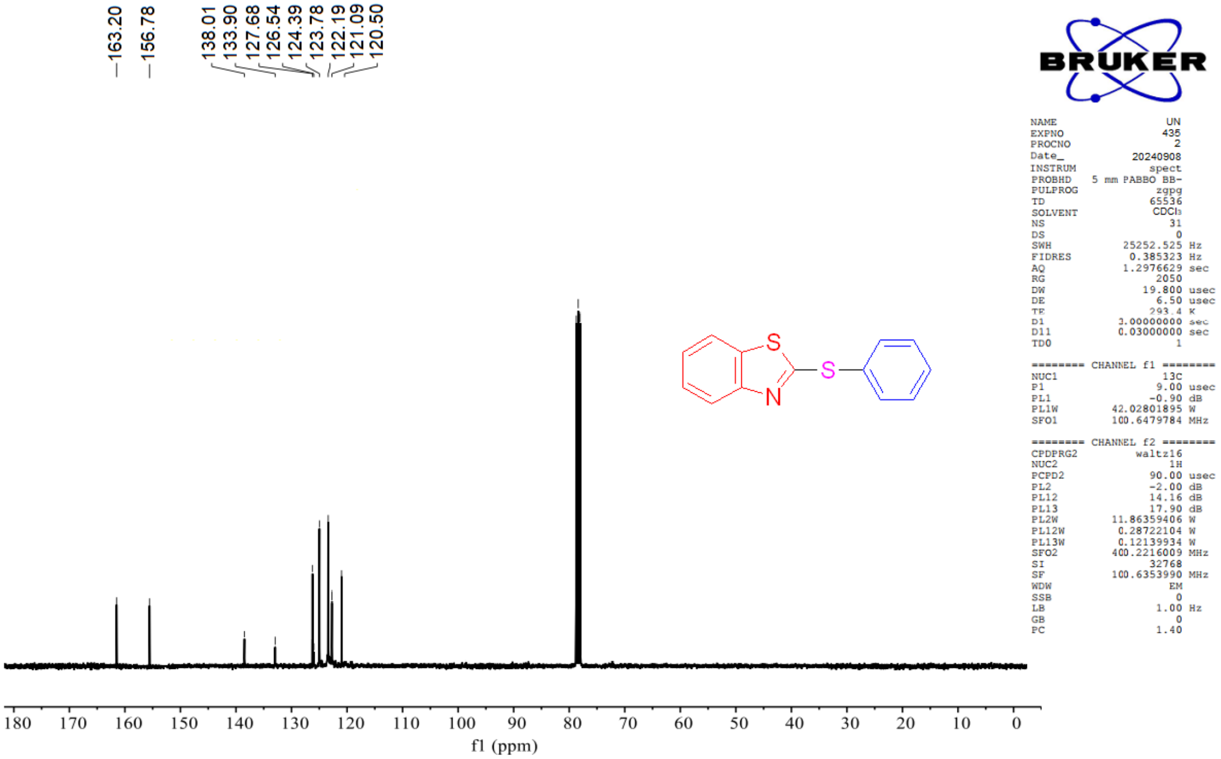
163.20
156.78
138.01
133.90
127.68
126.54
124.39
123.78
122.19
121.09
120.50
S
N
S
f1 (ppm)
BRUKER
NAME UN
EXPNO 435
PROCNO 2
Date_ 20240908
INSTRUM spect
PROBHD 5 mm PABBO BB-
PULPROG zgpg
TD 65536
SOLVENT CDCl3
NS 31
DS 0
SWH 25252.525 Hz
FIDRES 0.385323 Hz
AQ 1.2976629 sec
RG 2050
DW 19.800 usec
DE 6.50 usec
TE 293.4 K
D1 3.00000000 sec
D11 0.03000000 sec
TD0 1
======== CHANNEL f1 ========
NUC1 13C
P1 9.00 usec
PL1 -0.90 dB
PL1W 42.02801895 W
SFO1 100.6479784 MHz
======== CHANNEL f2 ========
CPDPRG2 waltz16
NUC2 1H
PCPD2 90.00 usec
PL2 -2.00 dB
PL12 14.16 dB
PL13 17.90 dB
PL2W 11.86359406 W
PL12W 0.28722104 W
PL13W 0.12139934 W
SFO2 400.2216009 MHz
SI 32768
SF 100.6353990 MHz
WDW EM
SSB 0
LB 1.00 Hz
GB 0
PC 1.40

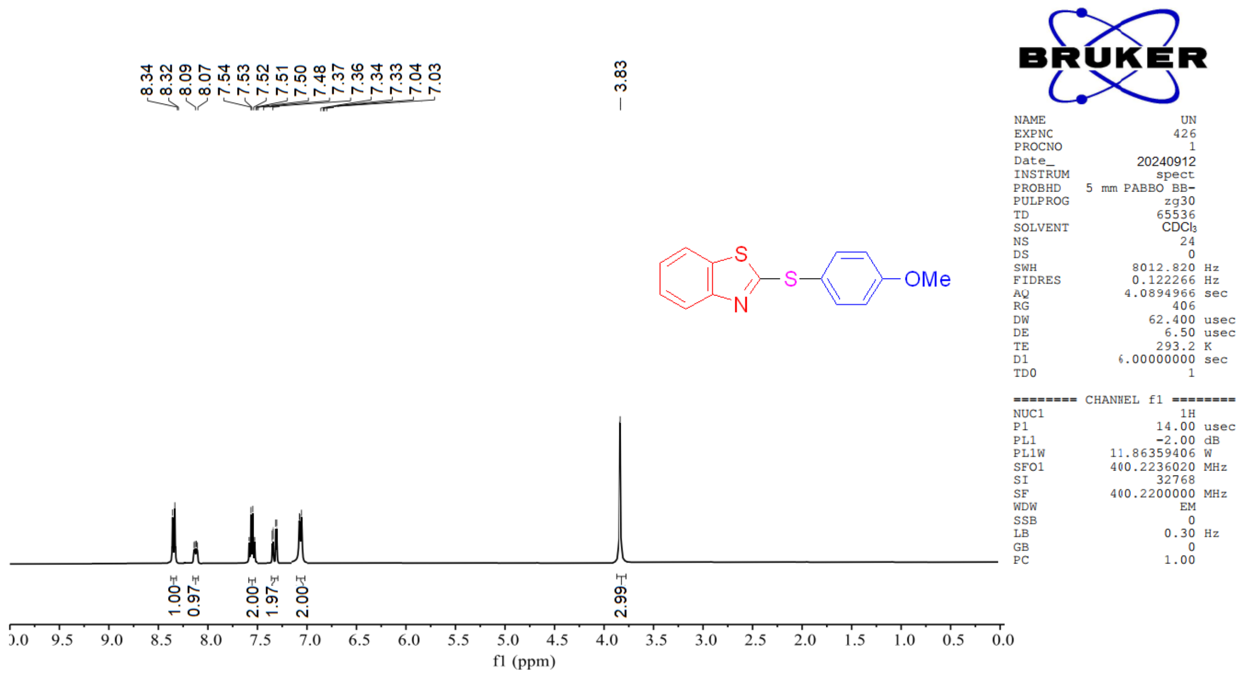
8.34
8.32
8.09
8.07
7.54
7.53
7.52
7.51
7.50
7.48
7.37
7.36
7.34
7.33
7.04
7.03
3.83
S
N
S
OMe
BRUKER
NAME UN
EXPNO 426
PROCNO 1
Date_ 20240912
INSTRUM spect
PROBHD 5 mm PABBO BB-
PULPROG zg30
TD 65536
SOLVENT CDCl3
NS 24
DS 0
SWH 8012.820 Hz
FIDRES 0.122266 Hz
AQ 4.0894966 sec
RG 406
DW 62.400 usec
DE 6.50 usec
TE 293.2 K
D1 6.00000000 sec
TD0 1
======== CHANNEL f1 ========
NUC1 1H
P1 14.00 usec
PL1 -2.00 dB
PL1W 11.86359406 W
SFO1 400.2236020 MHz
SI 32768
SF 400.2200000 MHz
WDW EM
SSB 0
LB 0.30 Hz
GB 0
PC 1.00
1.00
0.97
2.00
1.97
2.00
2.99
9.5 9.0 8.5 8.0 7.5 7.0 6.5 6.0 5.5 5.0 4.5 4.0 3.5 3.0 2.5 2.0 1.5 1.0 0.5 0.0
f1 (ppm)

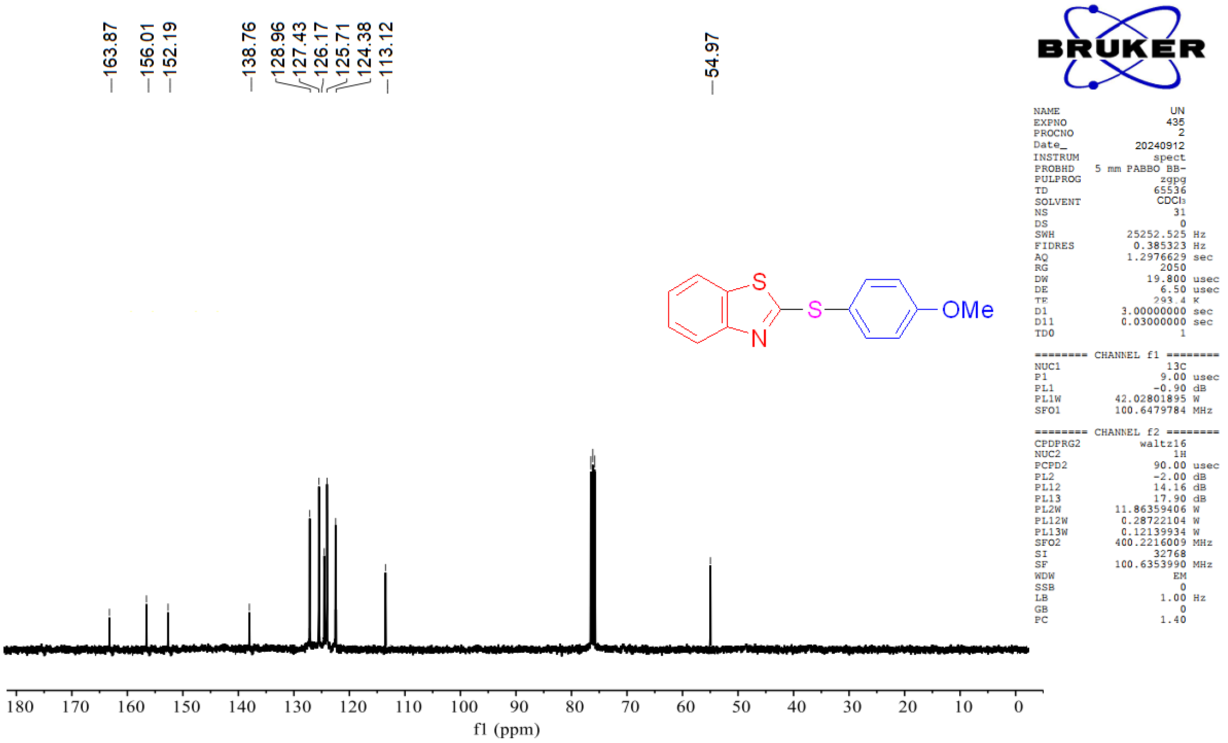
163.87
156.01
152.19
138.76
128.96
127.43
126.17
125.71
124.38
113.12
54.97
S
N
S
OMe
BRUKER
NAME UN
EXPNO 435
PROCNO 2
Date_ 20240912
INSTRUM spect
PROBHD 5 mm PABBO BB-
PULPROG zgpg
TD 65536
SOLVENT CDCl3
NS 31
DS 0
SWH 25252.525 Hz
FIDRES 0.385323 Hz
AQ 1.2976629 sec
RG 2050
DW 19.800 usec
DE 6.50 usec
D1 3.00000000 sec
D11 0.03000000 sec
TD0 1
======== CHANNEL f1 ========
NUC1 13C
P1 9.00 usec
PL1 -0.90 dB
PL1W 42.02801895 W
SFO1 100.6479784 MHz
======== CHANNEL f2 ========
CPDPRG2 waltz16
NUC2 1H
PCPD2 90.00 usec
PL2 -2.00 dB
PL12 14.16 dB
PL13 17.90 dB
PL2W 11.86359406 W
PL12W 0.28722104 W
PL13W 0.12139934 W
SFO2 400.2216009 MHz
SI 32768
SF 100.6353990 MHz
WDW EM
SSB 0
LB 1.00 Hz
GB 0
PC 1.40
180 170 160 150 140 130 120 110 100 90 80 70 60 50 40 30 20 10 0
f1 (ppm)

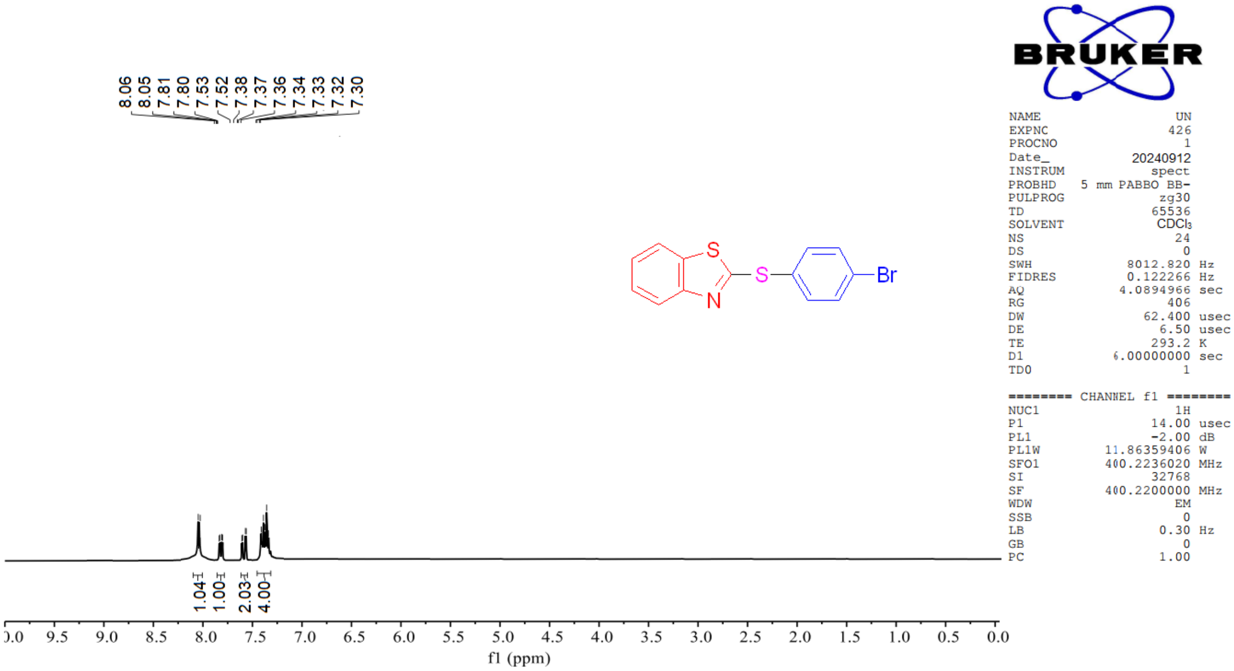
8.06
8.05
7.81
7.80
7.53
7.52
7.38
7.37
7.36
7.34
7.33
7.32
7.30
S
N
S
Br
1.04
1.00
2.03
4.00
f1 (ppm)
BRUKER
NAME UN
EXPNO 426
PROCNO 1
Date_ 20240912
INSTRUM spect
PROBHD 5 mm PABBO BB-
PULPROG zg30
TD 65536
SOLVENT CDCl3
NS 24
DS 0
SWH 8012.820 Hz
FIDRES 0.122266 Hz
AQ 4.0894966 sec
RG 406
DW 62.400 usec
DE 6.50 usec
TE 293.2 K
D1 6.00000000 sec
TD0 1
======== CHANNEL f1 ========
NUC1 1H
P1 14.00 usec
PL1 -2.00 dB
PL1W 11.86359406 W
SFO1 400.2236020 MHz
SI 32768
SF 400.2200000 MHz
WDW EM
SSB 0
LB 0.30 Hz
GB 0
PC 1.00

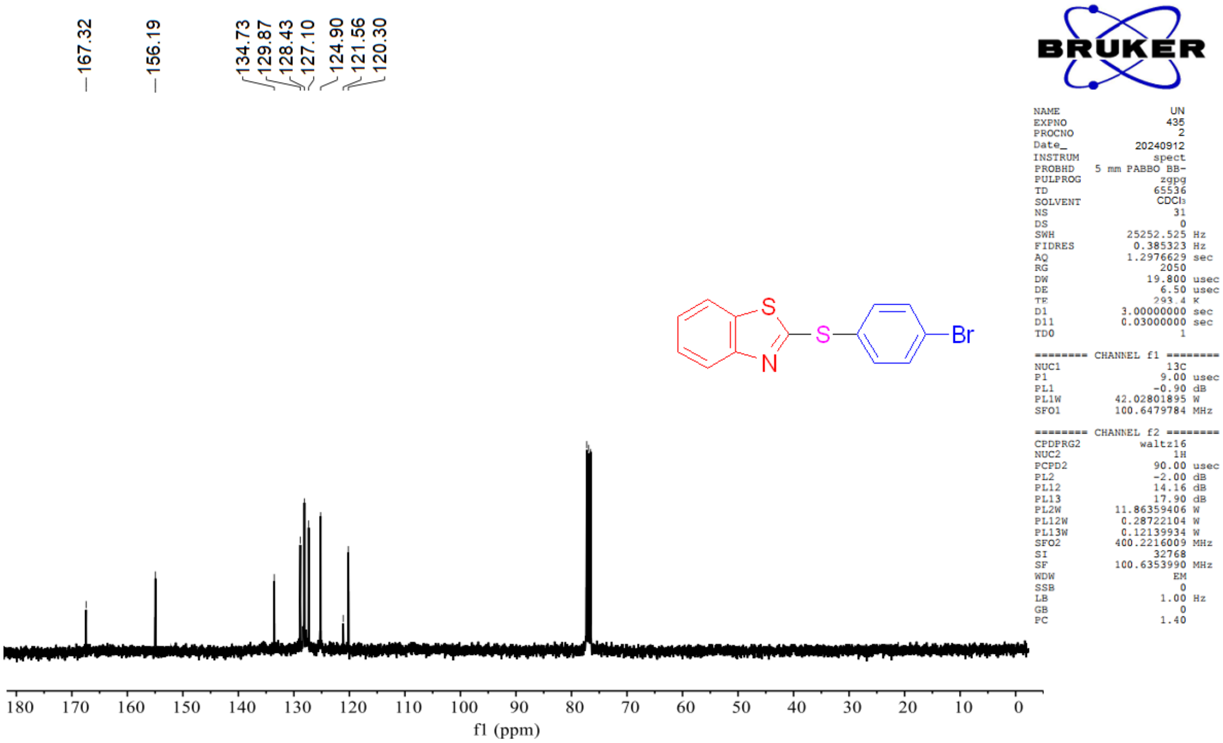
167.32
156.19
134.73
129.87
128.43
127.10
124.90
121.56
120.30
S
N
S
Br
f1 (ppm)
BRUKER
NAME UN
EXPNO 435
PROCNO 2
Date_ 20240912
INSTRUM spect
PROBHD 5 mm PABBO BB-
PULPROG zgpg
TD 65536
SOLVENT CDCl3
NS 31
DS 0
SWH 25252.525 Hz
FIDRES 0.385323 Hz
AQ 1.2976629 sec
RG 2050
DW 19.800 usec
DE 6.50 usec
TE 293.4 K
D1 3.00000000 sec
D11 0.03000000 sec
TD0 1
======== CHANNEL f1 ========
NUC1 13C
P1 9.00 usec
PL1 -0.90 dB
PL1W 42.02801895 W
SFO1 100.6479784 MHz
======== CHANNEL f2 ========
CPDPRG2 waltz16
NUC2 1H
PCPD2 90.00 usec
PL2 -2.00 dB
PL12 14.16 dB
PL13 17.90 dB
PL2W 11.86359406 W
PL12W 0.28722104 W
PL13W 0.12139934 W
SFO2 400.2216009 MHz
SI 32768
SF 100.6353990 MHz
WDW EM
SSB 0
LB 1.00 Hz
GB 0
PC 1.40

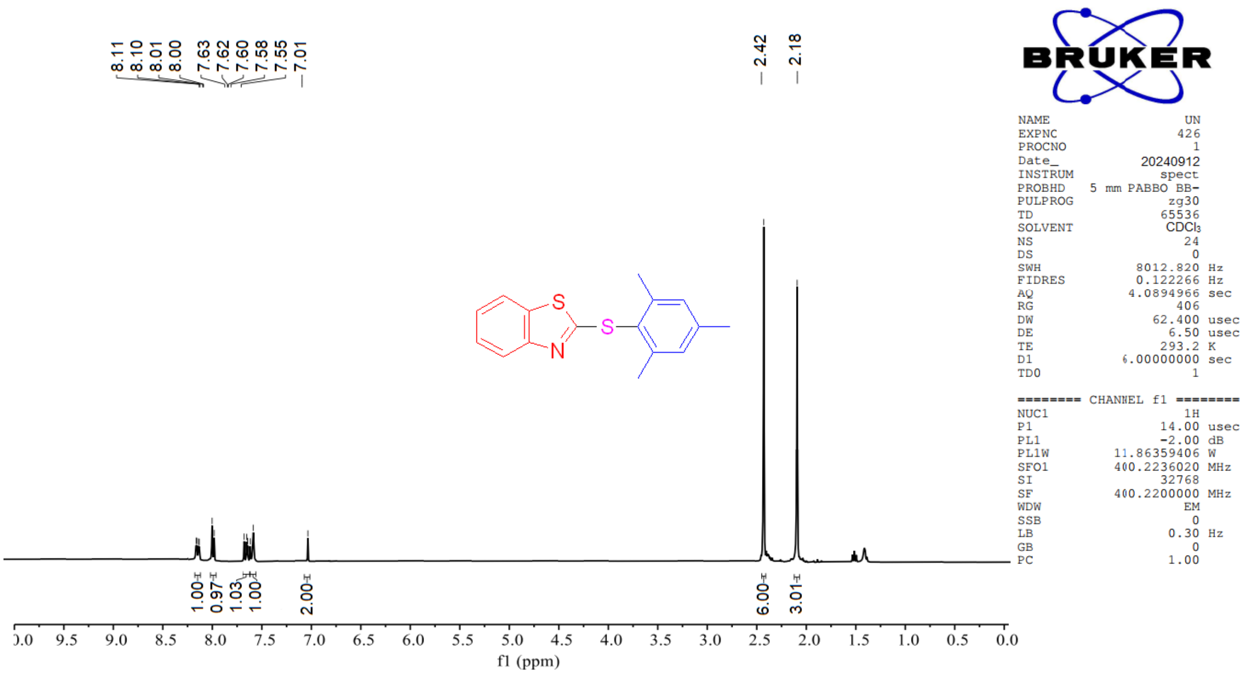
8.11
8.10
8.01
8.00
7.63
7.62
7.60
7.58
7.55
7.01
2.42
2.18
1.00
0.97
1.03
1.00
2.00
6.00
3.01
f1 (ppm)
BRUKER
NAME UN
EXPNO 426
PROCNO 1
Date_ 20240912
INSTRUM spect
PROBHD 5 mm PABBO BB-
PULPROG zg30
TD 65536
SOLVENT CDCl3
NS 24
DS 0
SWH 8012.820 Hz
FIDRES 0.122266 Hz
AQ 4.0894966 sec
RG 406
DW 62.400 usec
DE 6.50 usec
TE 293.2 K
D1 6.00000000 sec
TD0 1
======== CHANNEL f1 ========
NUC1 1H
P1 14.00 usec
PL1 -2.00 dB
PL1W 11.86359406 W
SFO1 400.2236020 MHz
SI 32768
SF 400.2200000 MHz
WDW EM
SSB 0
LB 0.30 Hz
GB 0
PC 1.00

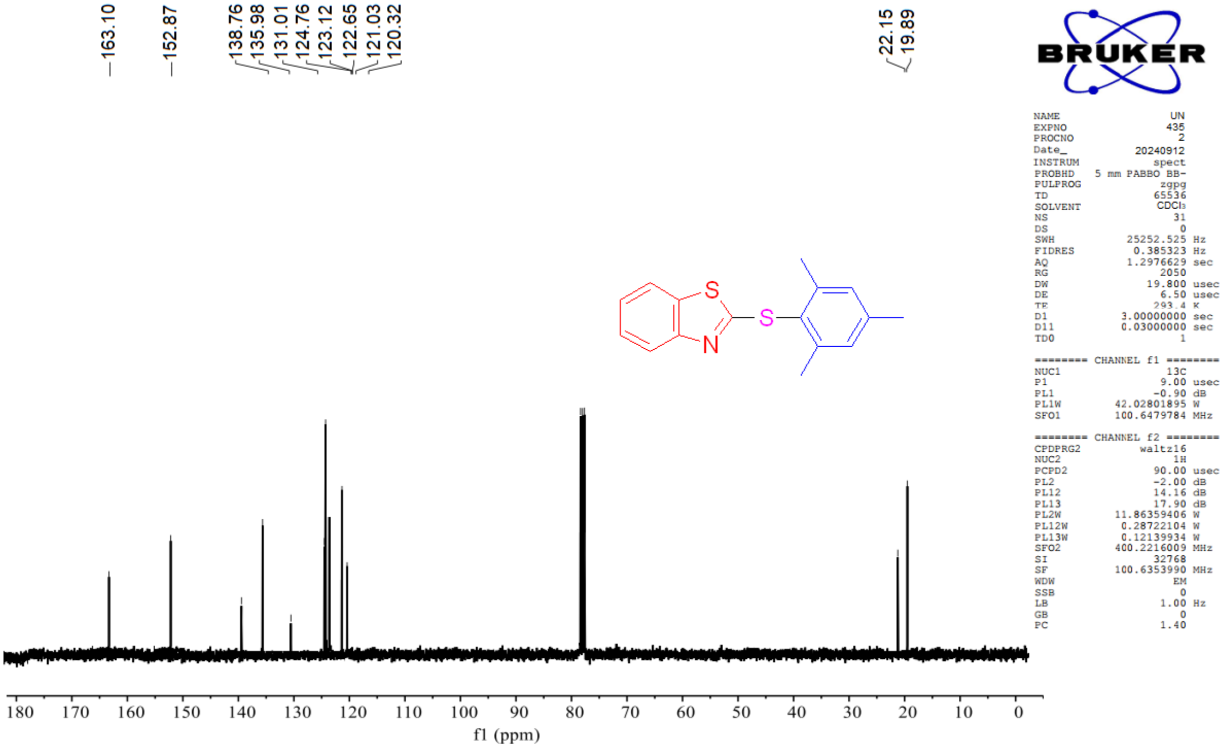
163.10
152.87
138.76
135.98
131.01
124.76
123.12
122.65
121.03
120.32
22.15
19.89
f1 (ppm)
BRUKER
NAME UN
EXPNO 435
PROCNO 2
Date_ 20240912
INSTRUM spect
PROBHD 5 mm PABBO BB-
PULPROG zgpg
TD 65536
SOLVENT CDCl3
NS 31
DS 0
SWH 25252.525 Hz
FIDRES 0.385323 Hz
AQ 1.2976629 sec
RG 2050
DW 19.800 usec
DE 6.50 usec
TE 293.4 K
D1 3.00000000 sec
D11 0.03000000 sec
TD0 1
======== CHANNEL f1 ========
NUC1 13C
P1 9.00 usec
PL1 -0.90 dB
PL1W 42.02801895 W
SFO1 100.6479784 MHz
======== CHANNEL f2 ========
CPDPRG2 waltz16
NUC2 1H
PCPD2 90.00 usec
PL2 -2.00 dB
PL12 14.16 dB
PL13 17.90 dB
PL2W 11.86359406 W
PL12W 0.28722104 W
PL13W 0.12139934 W
SFO2 400.2216009 MHz
SI 32768
SF 100.6353990 MHz
WDW EM
SSB 0
LB 1.00 Hz
GB 0
PC 1.40

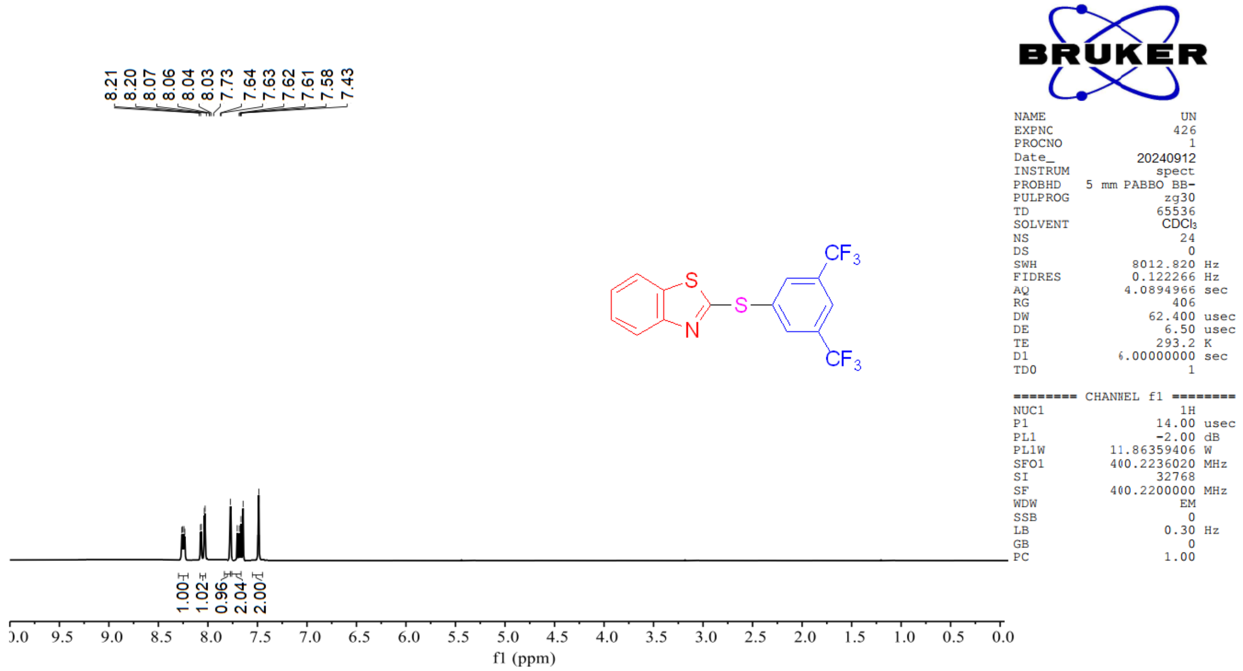
8.21
8.20
8.07
8.06
8.04
8.03
7.73
7.64
7.63
7.62
7.61
7.58
7.43
1.00
1.02
0.96
2.04
2.00
S
N
S
CF3
CF3
f1 (ppm)
BRUKER
NAME UN
EXPNC 426
PROCNO 1
Date_ 20240912
INSTRUM spect
PROBHD 5 mm PABBO BB-
PULPROG zg30
TD 65536
SOLVENT CDCl3
NS 24
DS 0
SWH 8012.820 Hz
FIDRES 0.122266 Hz
AQ 4.0894966 sec
RG 406
DW 62.400 usec
DE 6.50 usec
TE 293.2 K
D1 6.00000000 sec
TD0 1
======== CHANNEL f1 ========
NUC1 1H
P1 14.00 usec
PL1 -2.00 dB
PL1W 11.86359406 W
SFO1 400.2236020 MHz
SI 32768
SF 400.2200000 MHz
WDW EM
SSB 0
LB 0.30 Hz
GB 0
PC 1.00

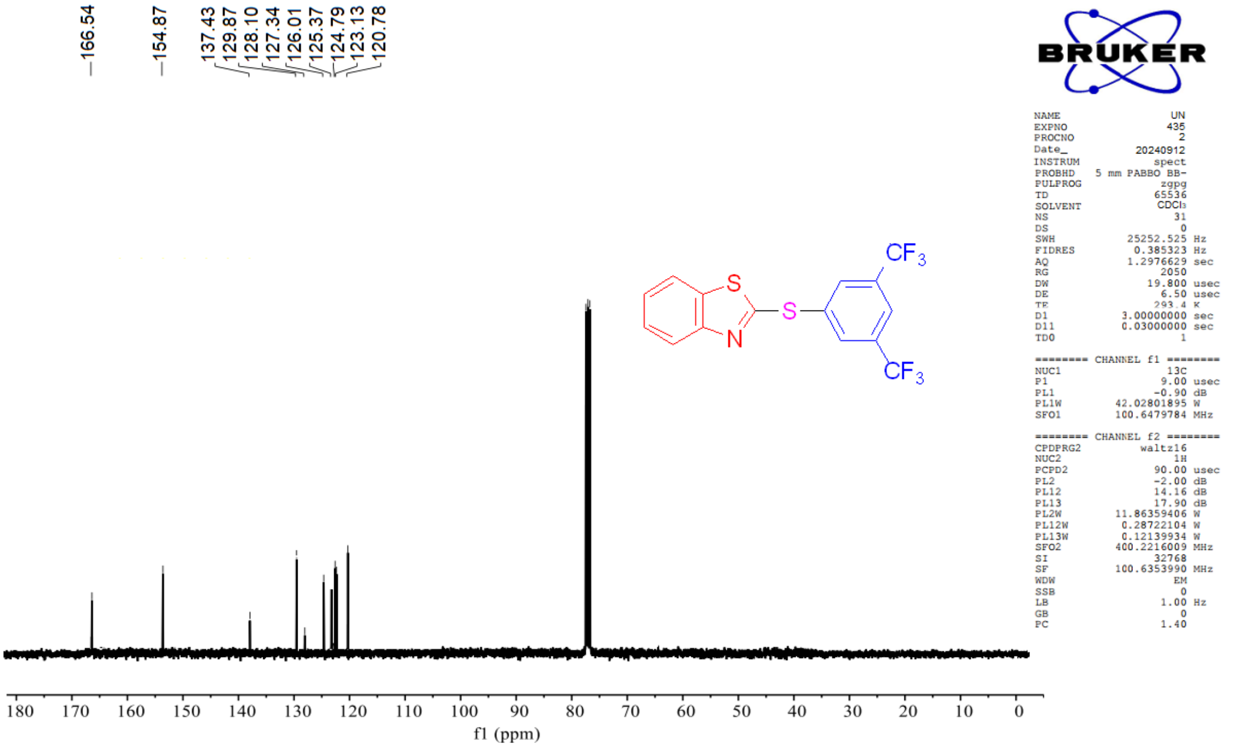
166.54
154.87
137.43
129.87
128.10
127.34
126.01
125.37
124.79
123.13
120.78
S
N
S
CF3
CF3
f1 (ppm)
BRUKER
NAME UN
EXPNO 435
PROCNO 2
Date_ 20240912
INSTRUM spect
PROBHD 5 mm PABBO BB-
PULPROG zgpg
TD 65536
SOLVENT CDCl3
NS 31
DS 0
SWH 25252.525 Hz
FIDRES 0.385323 Hz
AQ 1.2976629 sec
RG 2050
DW 19.800 usec
DE 6.50 usec
D1 3.00000000 sec
D11 0.03000000 sec
TD0 1
======== CHANNEL f1 ========
NUC1 13C
P1 9.00 usec
PL1 -0.90 dB
PL1W 42.02801895 W
SFO1 100.6479784 MHz
======== CHANNEL f2 ========
CPDPRG2 waltz16
NUC2 1H
PCPD2 90.00 usec
PL2 -2.00 dB
PL12 14.16 dB
PL13 17.90 dB
PL2W 11.86359406 W
PL12W 0.28722104 W
PL13W 0.12139934 W
SFO2 400.2216009 MHz
SI 32768
SF 100.6353990 MHz
WDW EM
SSB 0
LB 1.00 Hz
GB 0
PC 1.40

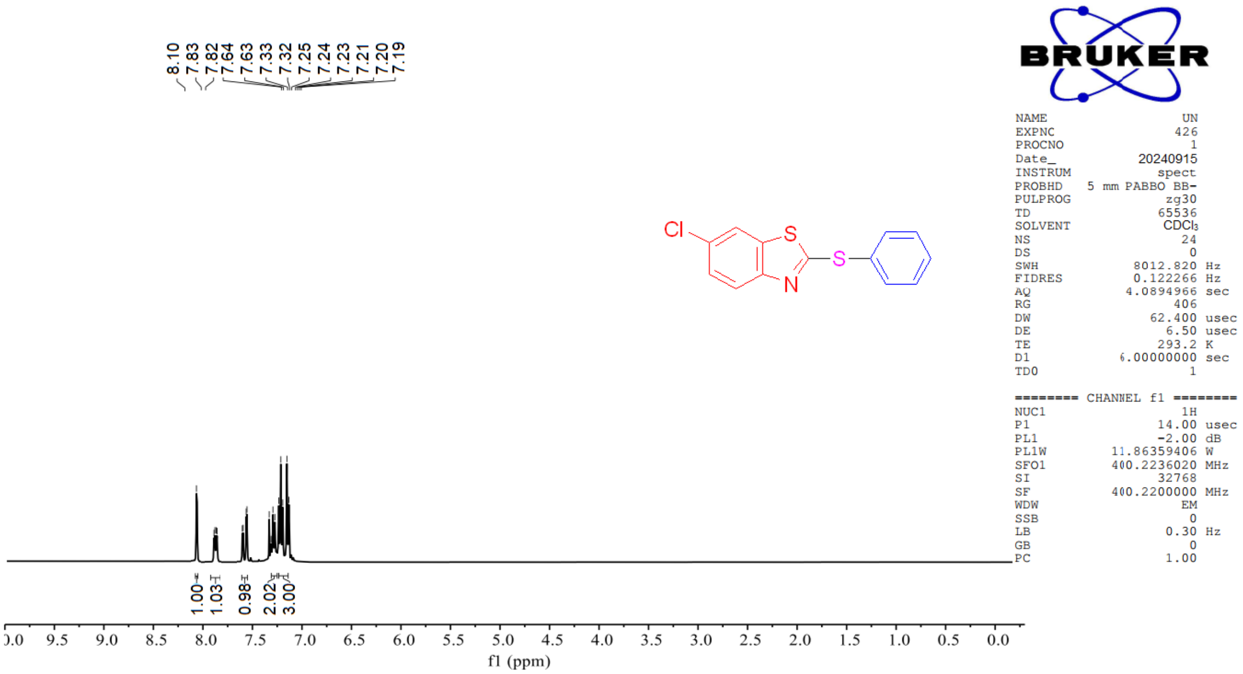
8.10
7.83
7.82
7.64
7.63
7.33
7.32
7.25
7.24
7.23
7.21
7.20
7.19
Cl
S
N
S
1.00
1.03
0.98
2.02
3.00
f1 (ppm)
NAME UN
EXPNC 426
PROCNO 1
Date_ 20240915
INSTRUM spect
PROBHD 5 mm PABBO BB-
PULPROG zg30
TD 65536
SOLVENT CDCl3
NS 24
DS 0
SWH 8012.820 Hz
FIDRES 0.122266 Hz
AQ 4.0894966 sec
RG 406
DW 62.400 usec
DE 6.50 usec
TE 293.2 K
D1 6.00000000 sec
TD0 1
======== CHANNEL f1 ========
NUC1 1H
P1 14.00 usec
PL1 -2.00 dB
PL1W 11.86359406 W
SFO1 400.2236020 MHz
SI 32768
SF 400.2200000 MHz
WDW EM
SSB 0
LB 0.30 Hz
GB 0
PC 1.00

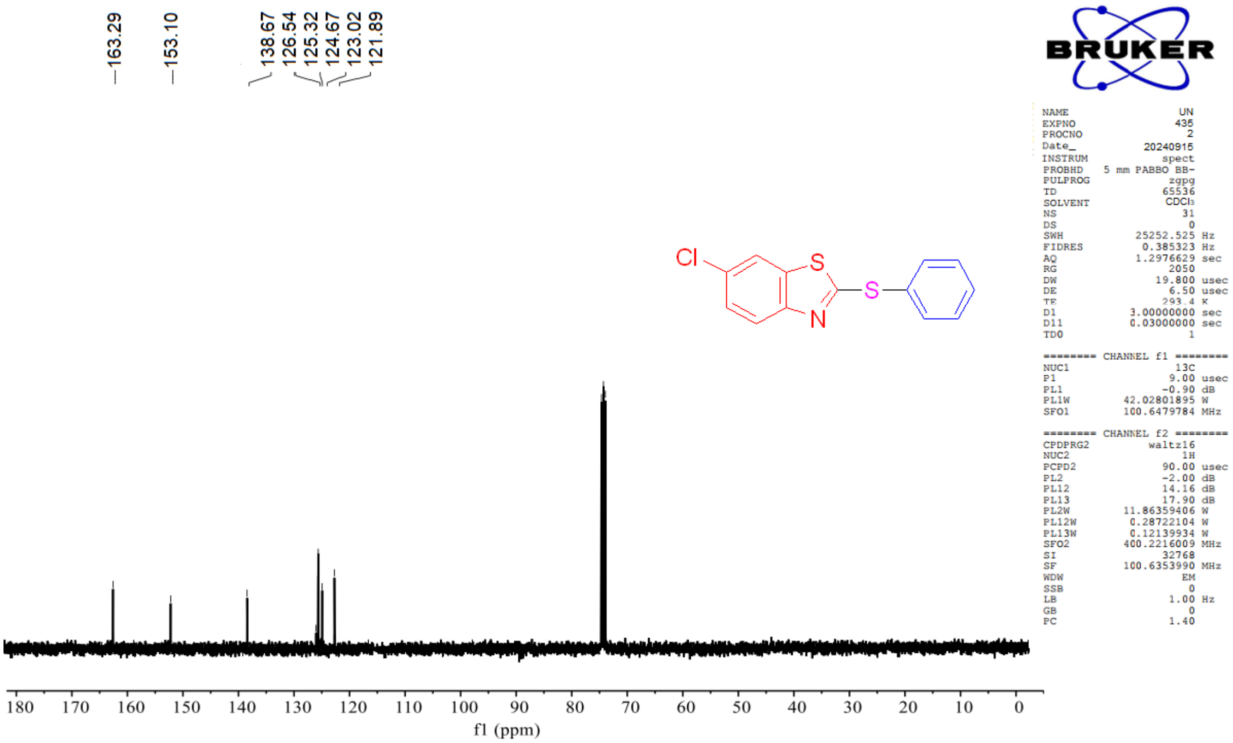
163.29
153.10
138.67
126.54
125.32
124.67
123.02
121.89
Cl
S
N
S
f1 (ppm)
NAME UN
EXPNO 435
PROCNO 2
Date_ 20240915
INSTRUM spect
PROBHD 5 mm PABBO BB-
PULPROG zgpg
TD 65536
SOLVENT CDCl3
NS 31
DS 0
SWH 25252.525 Hz
FIDRES 0.385323 Hz
AQ 1.2976629 sec
RG 2050
DW 19.800 usec
DE 6.50 usec
TE 293.4 K
D1 3.00000000 sec
D11 0.03000000 sec
TD0 1
======== CHANNEL f1 ========
NUC1 13C
P1 9.00 usec
PL1 -0.90 dB
PL1W 42.02801895 W
SFO1 100.6479784 MHz
======== CHANNEL f2 ========
CPDPRG2 waltz16
NUC2 1H
PCPD2 90.00 usec
PL2 -2.00 dB
PL12 14.16 dB
PL13 17.90 dB
PL2W 11.86359406 W
PL12W 0.28722104 W
PL13W 0.12139934 W
SFO2 400.2216009 MHz
SI 32768
SF 100.6353990 MHz
WDW EM
SSB 0
LB 1.00 Hz
GB 0
PC 1.40

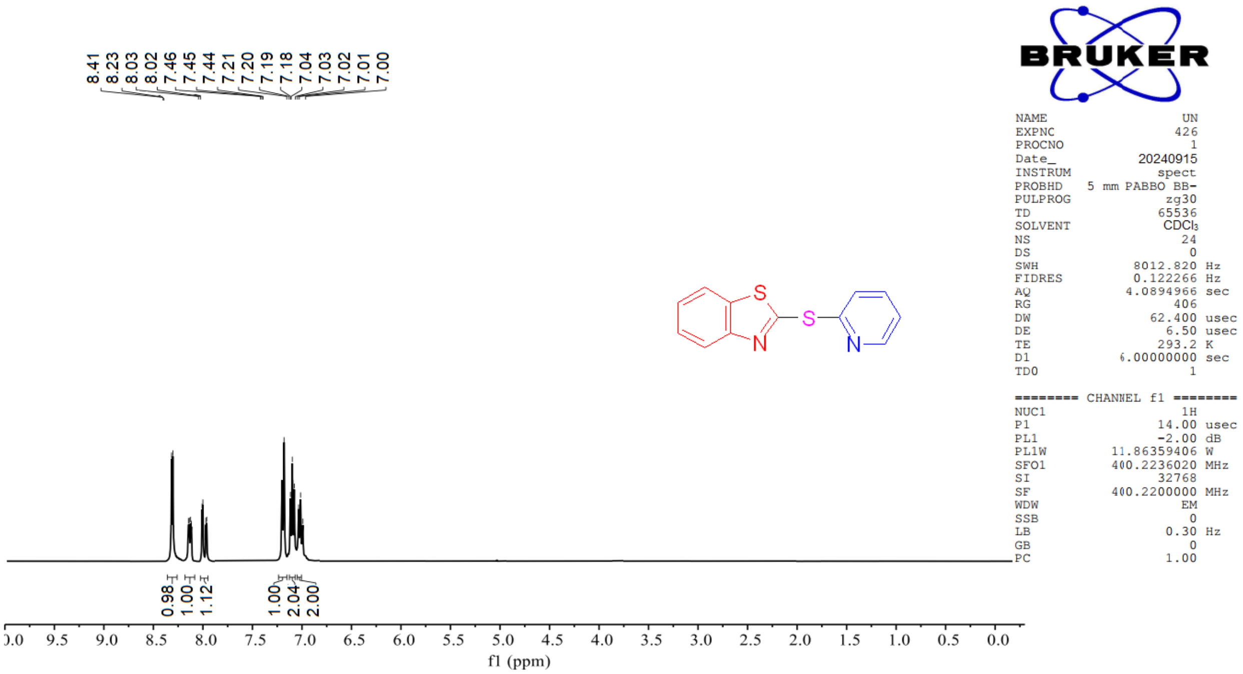
8.41
8.23
8.03
8.02
7.46
7.45
7.44
7.21
7.20
7.19
7.18
7.04
7.03
7.02
7.01
7.00
BRUKER
NAME UN
EXPNC 426
PROCNO 1
Date_ 20240915
INSTRUM spect
PROBHD 5 mm PABBO BB-
PULPROG zg30
TD 65536
SOLVENT CDCl3
NS 24
DS 0
SWH 8012.820 Hz
FIDRES 0.122266 Hz
AQ 4.0894966 sec
RG 406
DW 62.400 usec
DE 6.50 usec
TE 293.2 K
D1 6.00000000 sec
TD0 1
======== CHANNEL f1 ========
NUC1 1H
P1 14.00 usec
PL1 -2.00 dB
PL1W 11.86359406 W
SFO1 400.2236020 MHz
SI 32768
SF 400.2200000 MHz
WDW EM
SSB 0
LB 0.30 Hz
GB 0
PC 1.00
S
N
S
N
0.98
1.00
1.12
1.00
2.04
2.00
9.5 9.0 8.5 8.0 7.5 7.0 6.5 6.0 5.5 5.0 4.5 4.0 3.5 3.0 2.5 2.0 1.5 1.0 0.5 0.0
f1 (ppm)

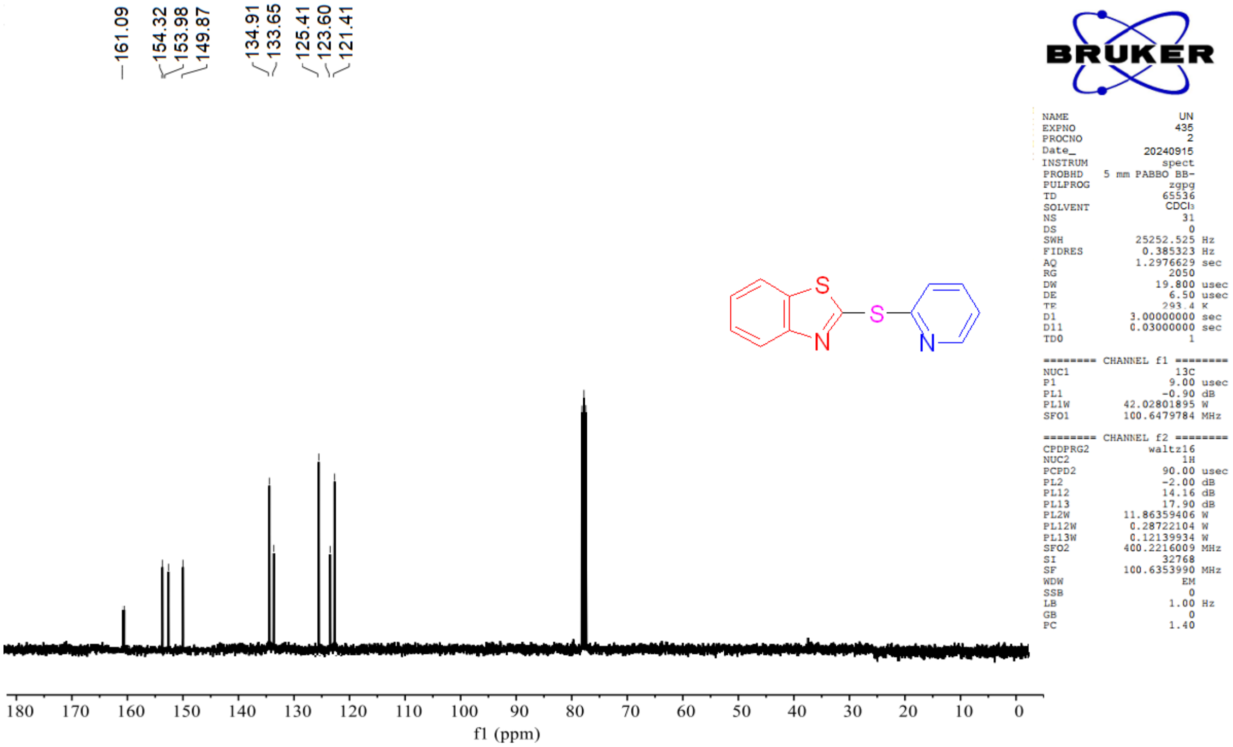
161.09
154.32
153.98
149.87
134.91
133.65
125.41
123.60
121.41
BRUKER
NAME UN
EXPNO 435
PROCNO 2
Date_ 20240915
INSTRUM spect
PROBHD 5 mm PABBO BB-
PULPROG zgpg
TD 65536
SOLVENT CDCl3
NS 31
DS 0
SWH 25252.525 Hz
FIDRES 0.385323 Hz
AQ 1.2976629 sec
RG 2050
DW 19.800 usec
DE 6.50 usec
TE 293.4 K
D1 3.00000000 sec
D11 0.03000000 sec
TD0 1
======== CHANNEL f1 ========
NUC1 13C
P1 9.00 usec
PL1 -0.90 dB
PL1W 42.02801895 W
SFO1 100.6479784 MHz
======== CHANNEL f2 ========
CPDPRG2 waltz16
NUC2 1H
PCPD2 90.00 usec
PL2 -2.00 dB
PL12 14.16 dB
PL13 17.90 dB
PL2W 11.86359406 W
PL12W 0.28722104 W
PL13W 0.12139934 W
SFO2 400.2216009 MHz
SI 32768
SF 100.6353990 MHz
WDW EM
SSB 0
LB 1.00 Hz
GB 0
PC 1.40
S
N
S
N
180 170 160 150 140 130 120 110 100 90 80 70 60 50 40 30 20 10 0
f1 (ppm)

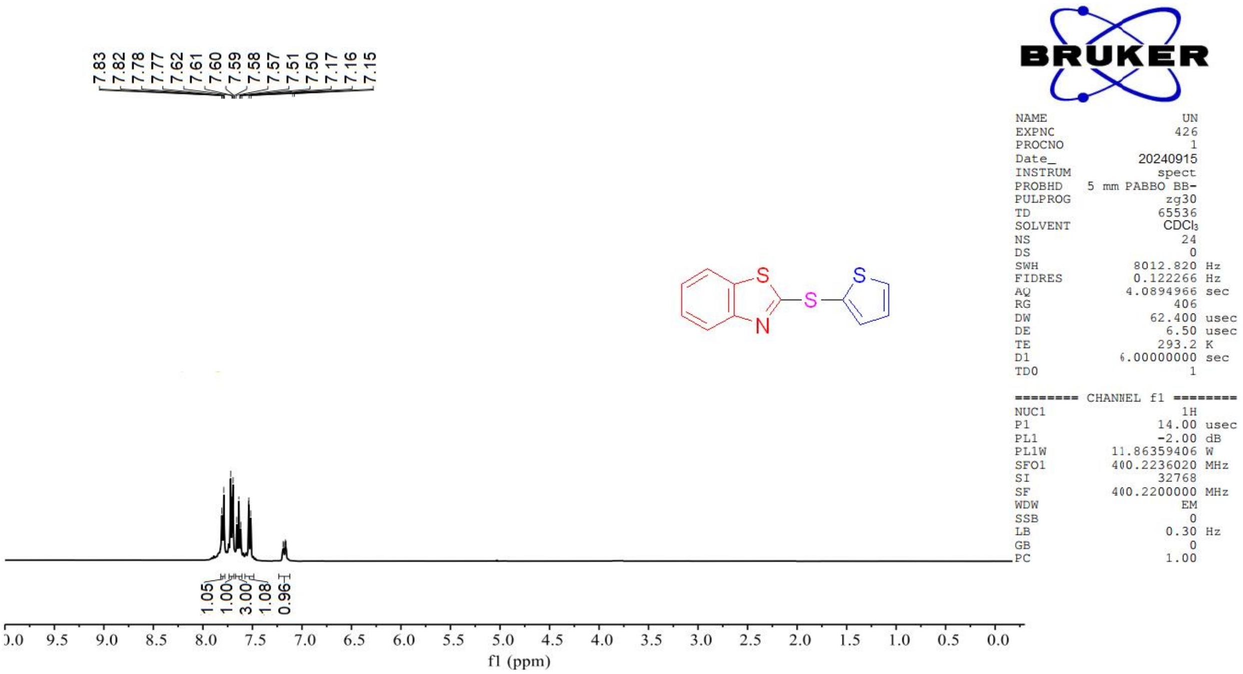
7.83
7.82
7.78
7.77
7.62
7.61
7.60
7.59
7.58
7.57
7.51
7.50
7.17
7.16
7.15
1.05
1.00
3.00
1.08
0.96
f1 (ppm)
S
N
S
S
BRUKER
NAME UN
EXPNO 426
PROCNO 1
Date_ 20240915
INSTRUM spect
PROBHD 5 mm PABBO BB-
PULPROG zg30
TD 65536
SOLVENT CDCl3
NS 24
DS 0
SWH 8012.820 Hz
FIDRES 0.122266 Hz
AQ 4.0894966 sec
RG 406
DW 62.400 usec
DE 6.50 usec
TE 293.2 K
D1 6.00000000 sec
TD0 1
======== CHANNEL f1 ========
NUC1 1H
P1 14.00 usec
PL1 -2.00 dB
PL1W 11.86359406 W
SFO1 400.2236020 MHz
SI 32768
SF 400.2200000 MHz
WDW EM
SSB 0
LB 0.30 Hz
GB 0
PC 1.00

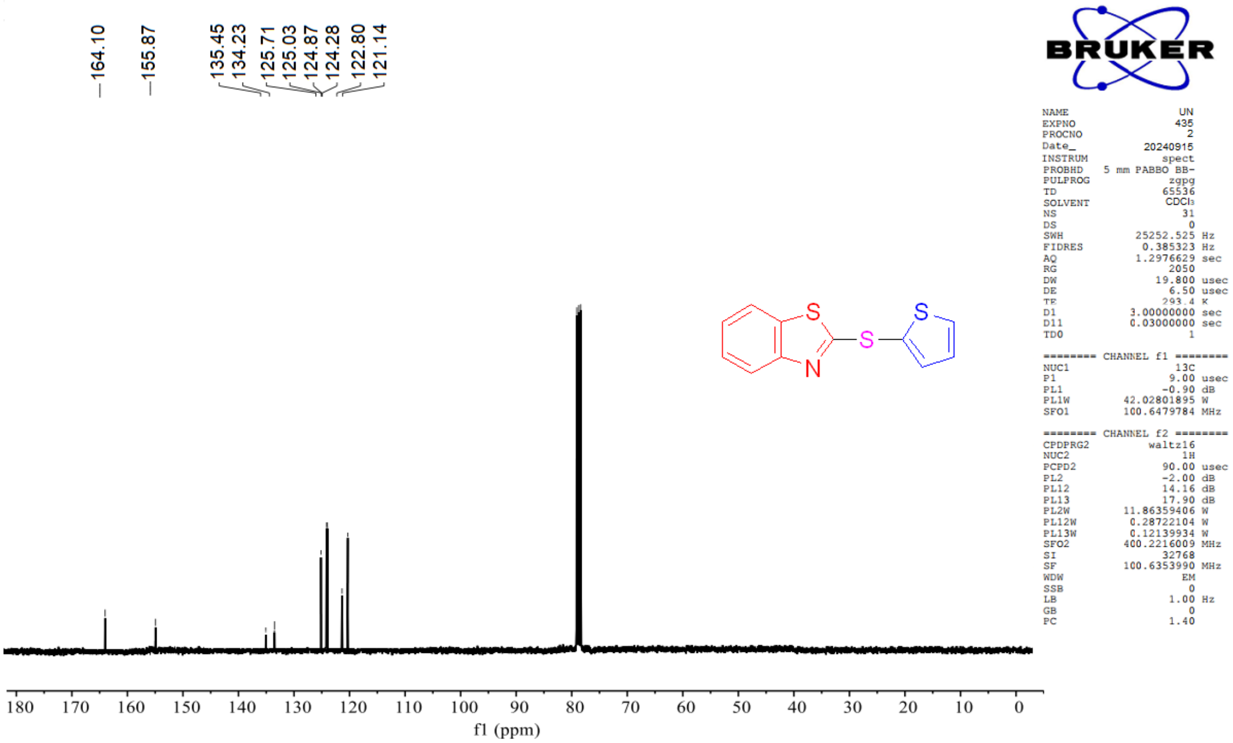
164.10
155.87
135.45
134.23
125.71
125.03
124.87
124.28
122.80
121.14
f1 (ppm)
S
N
S
S
BRUKER
NAME UN
EXPNO 435
PROCNO 2
Date_ 20240915
INSTRUM spect
PROBHD 5 mm PABBO BB-
PULPROG zgpg
TD 65536
SOLVENT CDCl3
NS 31
DS 0
SWH 25252.525 Hz
FIDRES 0.385323 Hz
AQ 1.2976629 sec
RG 2050
DW 19.800 usec
DE 6.50 usec
TE 293.4 K
D1 3.00000000 sec
D11 0.03000000 sec
TD0 1
======== CHANNEL f1 ========
NUC1 13C
P1 9.00 usec
PL1 -0.90 dB
PL1W 42.02801895 W
SFO1 100.6479784 MHz
======== CHANNEL f2 ========
CPDPRG2 waltz16
NUC2 1H
PCPD2 90.00 usec
PL2 -2.00 dB
PL12 14.16 dB
PL13 17.90 dB
PL2W 11.86359406 W
PL12W 0.28722104 W
PL13W 0.12139934 W
SFO2 400.2216009 MHz
SI 32768
SF 100.6353990 MHz
WDW EM
SSB 0
LB 1.00 Hz
GB 0
PC 1.40

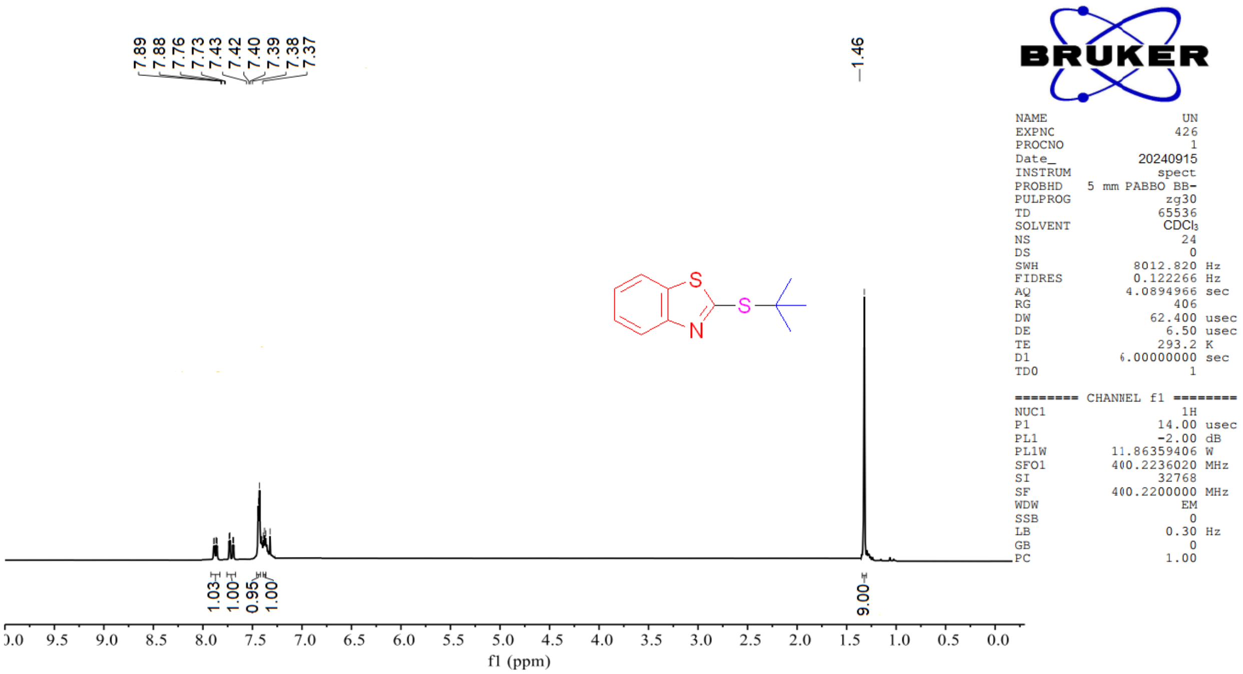
7.89
7.88
7.76
7.73
7.43
7.42
7.40
7.39
7.38
7.37
1.46
S
N
S
1.03
1.00
0.95
1.00
9.00
f1 (ppm)
BRUKER
NAME UN
EXPNO 426
PROCNO 1
Date_ 20240915
INSTRUM spect
PROBHD 5 mm PABBO BB-
PULPROG zg30
TD 65536
SOLVENT CDCl3
NS 24
DS 0
SWH 8012.820 Hz
FIDRES 0.122266 Hz
AQ 4.0894966 sec
RG 406
DW 62.400 usec
DE 6.50 usec
TE 293.2 K
D1 6.00000000 sec
TD0 1
======== CHANNEL f1 ========
NUC1 1H
P1 14.00 usec
PL1 -2.00 dB
PL1W 11.86359406 W
SFO1 400.2236020 MHz
SI 32768
SF 400.2200000 MHz
WDW EM
SSB 0
LB 0.30 Hz
GB 0
PC 1.00

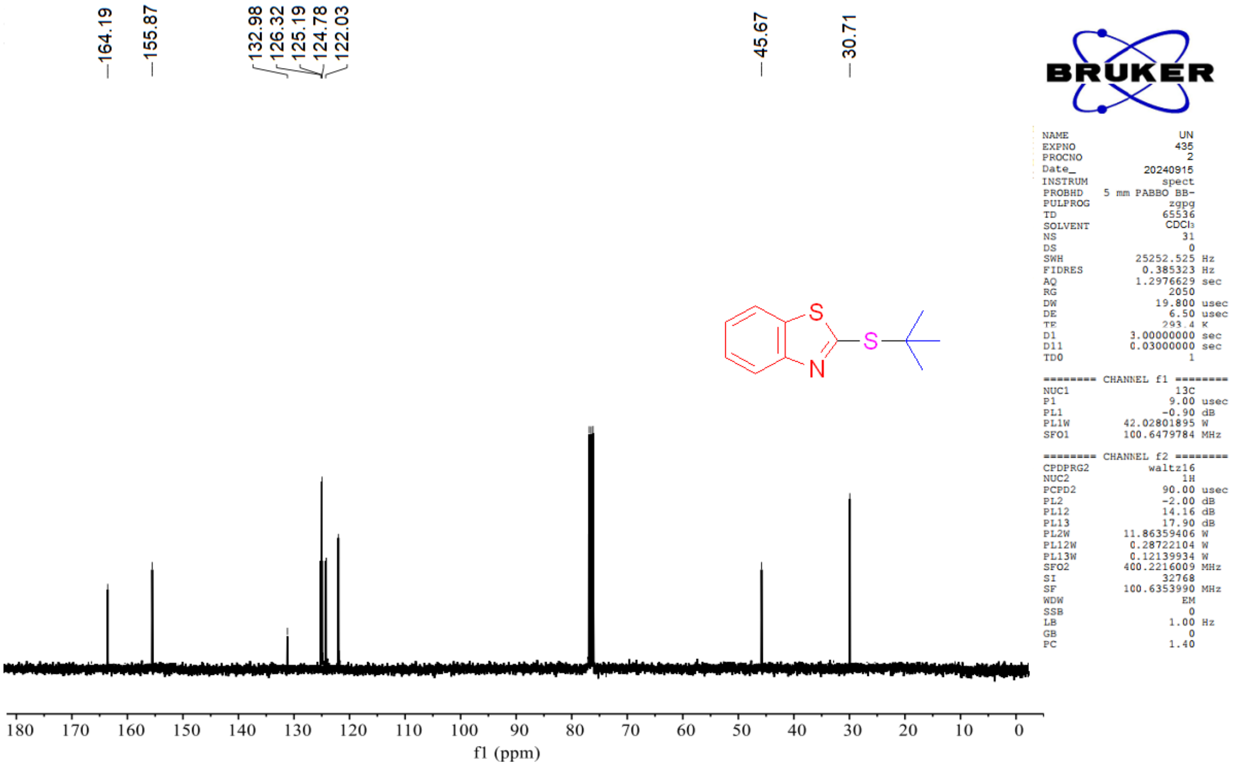
164.19
155.87
132.98
126.32
125.19
124.78
122.03
45.67
30.71
S
N
S
f1 (ppm)
BRUKER
NAME UN
EXPNO 435
PROCNO 2
Date_ 20240915
INSTRUM spect
PROBHD 5 mm PABBO BB-
PULPROG zgpg
TD 65536
SOLVENT CDCl3
NS 31
DS 0
SWH 25252.525 Hz
FIDRES 0.385323 Hz
AQ 1.2976629 sec
RG 2050
DW 19.800 usec
DE 6.50 usec
TE 293.4 K
D1 3.00000000 sec
D11 0.03000000 sec
TD0 1
======== CHANNEL f1 ========
NUC1 13C
P1 9.00 usec
PL1 -0.90 dB
PL1W 42.02801895 W
SFO1 100.6479784 MHz
======== CHANNEL f2 ========
CPDPRG2 waltz16
NUC2 1H
PCPD2 90.00 usec
PL2 -2.00 dB
PL12 14.16 dB
PL13 17.90 dB
PL2W 11.86359406 W
PL12W 0.28722104 W
PL13W 0.12139934 W
SFO2 400.2216009 MHz
SI 32768
SF 100.6353990 MHz
WDW EM
SSB 0
LB 1.00 Hz
GB 0
PC 1.40

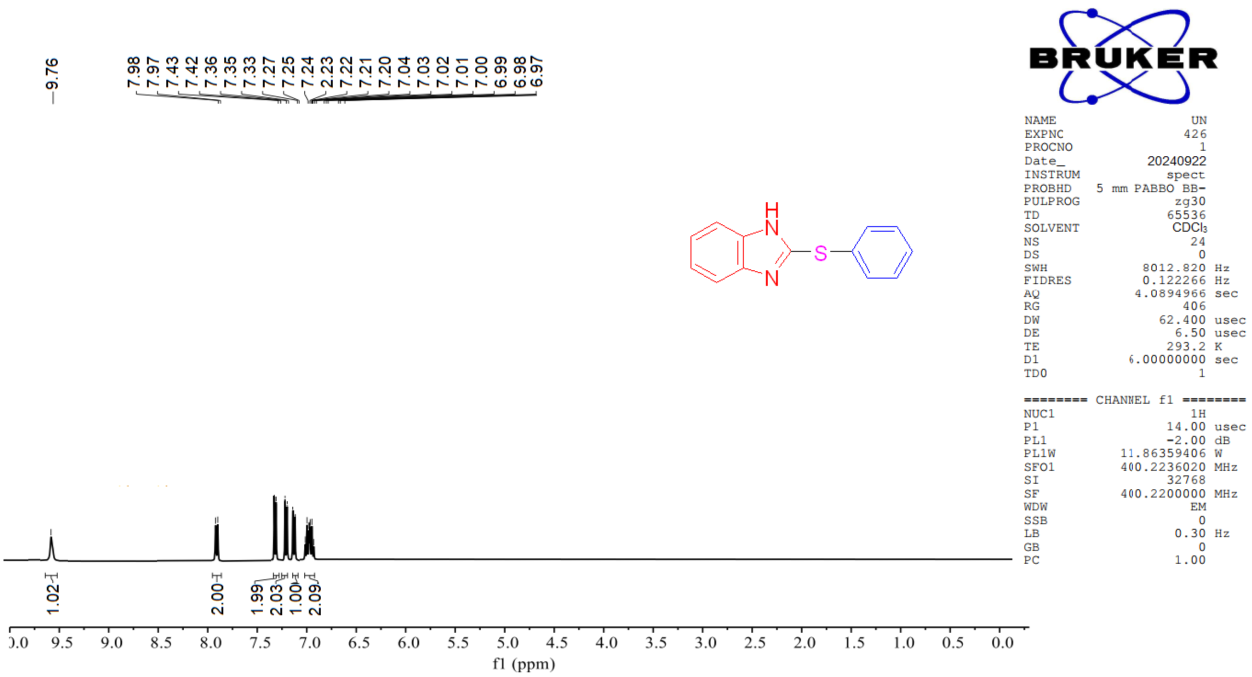

9.76
7.98
7.97
7.43
7.42
7.36
7.35
7.33
7.27
7.25
7.24
7.23
7.22
7.21
7.20
7.04
7.03
7.02
7.01
7.00
6.99
6.98
6.97
H
N
S
N
BRUKER
NAME UN
EXPNO 426
PROCNO 1
Date_ 20240922
INSTRUM spect
PROBHD 5 mm PABBO BB-
PULPROG zg30
TD 65536
SOLVENT CDCl3
NS 24
DS 0
SWH 8012.820 Hz
FIDRES 0.122266 Hz
AQ 4.0894966 sec
RG 406
DW 62.400 usec
DE 6.50 usec
TE 293.2 K
D1 6.00000000 sec
TD0 1
======== CHANNEL f1 ========
NUC1 1H
P1 14.00 usec
PL1 -2.00 dB
PL1W 11.86359406 W
SFO1 400.2236020 MHz
SI 32768
SF 400.2200000 MHz
WDW EM
SSB 0
LB 0.30 Hz
GB 0
PC 1.00
1.02
2.00
1.99
2.03
1.00
2.09
0.0 9.5 9.0 8.5 8.0 7.5 7.0 6.5 6.0 5.5 5.0 4.5 4.0 3.5 3.0 2.5 2.0 1.5 1.0 0.5 0.0
f1 (ppm)

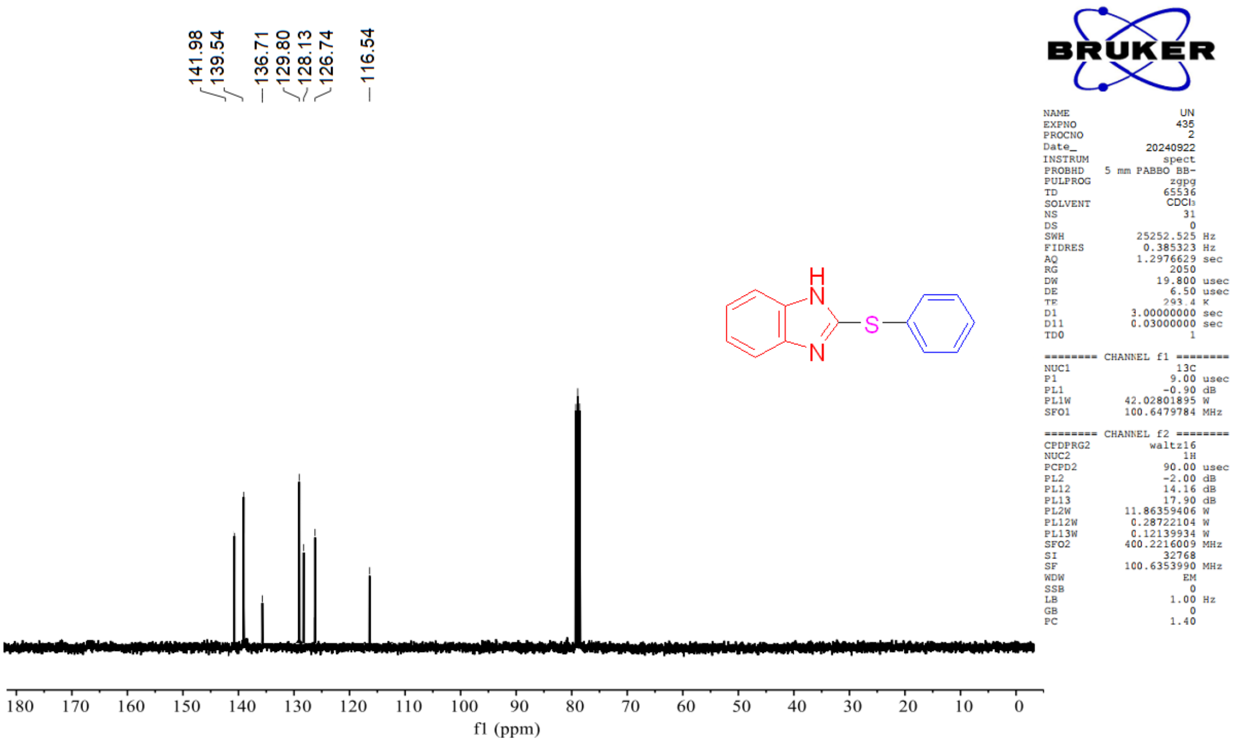

141.98
139.54
136.71
129.80
128.13
126.74
116.54
H
N
S
N
BRUKER
NAME UN
EXPNO 435
PROCNO 2
Date_ 20240922
INSTRUM spect
PROBHD 5 mm PABBO BB-
PULPROG zgpg
TD 65536
SOLVENT CDCl3
NS 31
DS 0
SWH 25252.525 Hz
FIDRES 0.385323 Hz
AQ 1.2976629 sec
RG 2050
DW 19.800 usec
DE 6.50 usec
TE 293.4 K
D1 3.00000000 sec
D11 0.03000000 sec
TD0 1
======== CHANNEL f1 ========
NUC1 13C
P1 9.00 usec
PL1 -0.90 dB
PL1W 42.02801895 W
SFO1 100.6479784 MHz
======== CHANNEL f2 ========
CPDPRG2 waltz16
NUC2 1H
PCPD2 90.00 usec
PL2 -2.00 dB
PL12 14.16 dB
PL13 17.90 dB
PL2W 11.86359406 W
PL12W 0.28722104 W
PL13W 0.12139934 W
SFO2 400.2216009 MHz
SI 32768
SF 100.6353990 MHz
WDW EM
SSB 0
LB 1.00 Hz
GB 0
PC 1.40
180 170 160 150 140 130 120 110 100 90 80 70 60 50 40 30 20 10 0
f1 (ppm)

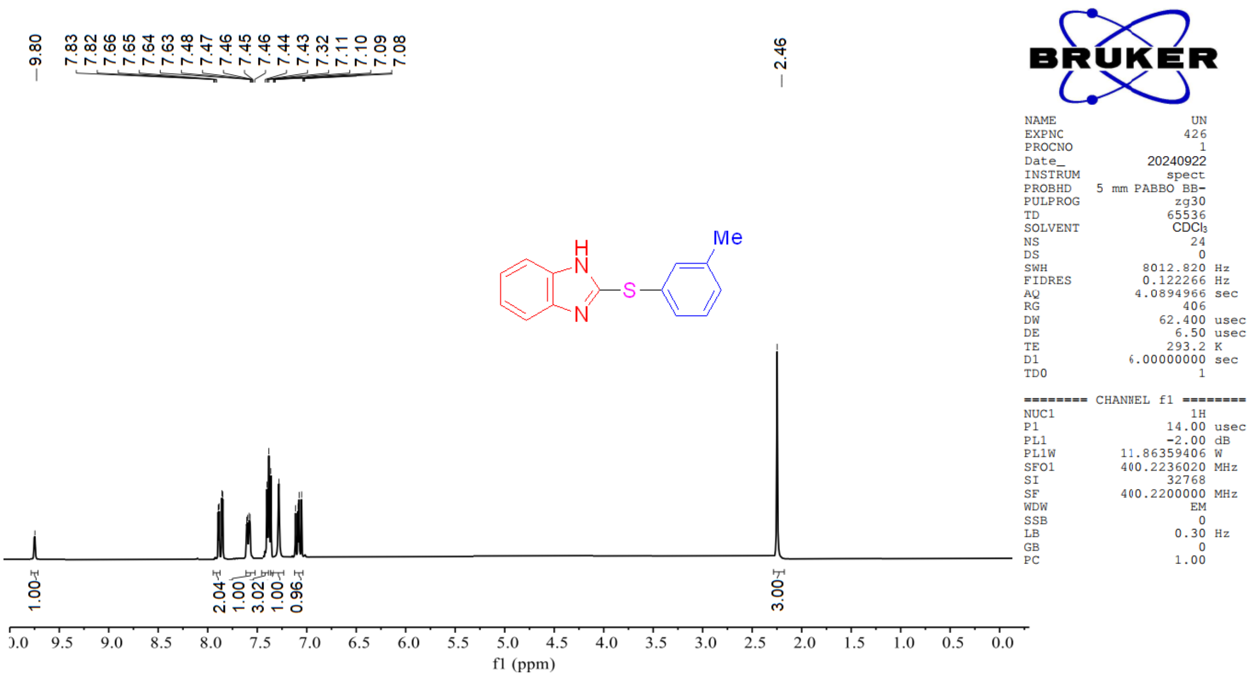

9.80
7.83
7.82
7.66
7.65
7.64
7.63
7.48
7.47
7.46
7.45
7.46
7.44
7.43
7.32
7.11
7.10
7.09
7.08
2.46
H
N
N
S
Me
1.00
2.04
1.00
3.02
1.00
0.96
3.00
f1 (ppm)
NAME UN
EXPNO 426
PROCNO 1
Date_ 20240922
INSTRUM spect
PROBHD 5 mm PABBO BB-
PULPROG zg30
TD 65536
SOLVENT CDCl3
NS 24
DS 0
SWH 8012.820 Hz
FIDRES 0.122266 Hz
AQ 4.0894966 sec
RG 406
DW 62.400 usec
DE 6.50 usec
TE 293.2 K
D1 6.00000000 sec
TD0 1
======== CHANNEL f1 ========
NUC1 1H
P1 14.00 usec
PL1 -2.00 dB
PL1W 11.86359406 W
SFO1 400.2236020 MHz
SI 32768
SF 400.2200000 MHz
WDW EM
SSB 0
LB 0.30 Hz
GB 0
PC 1.00

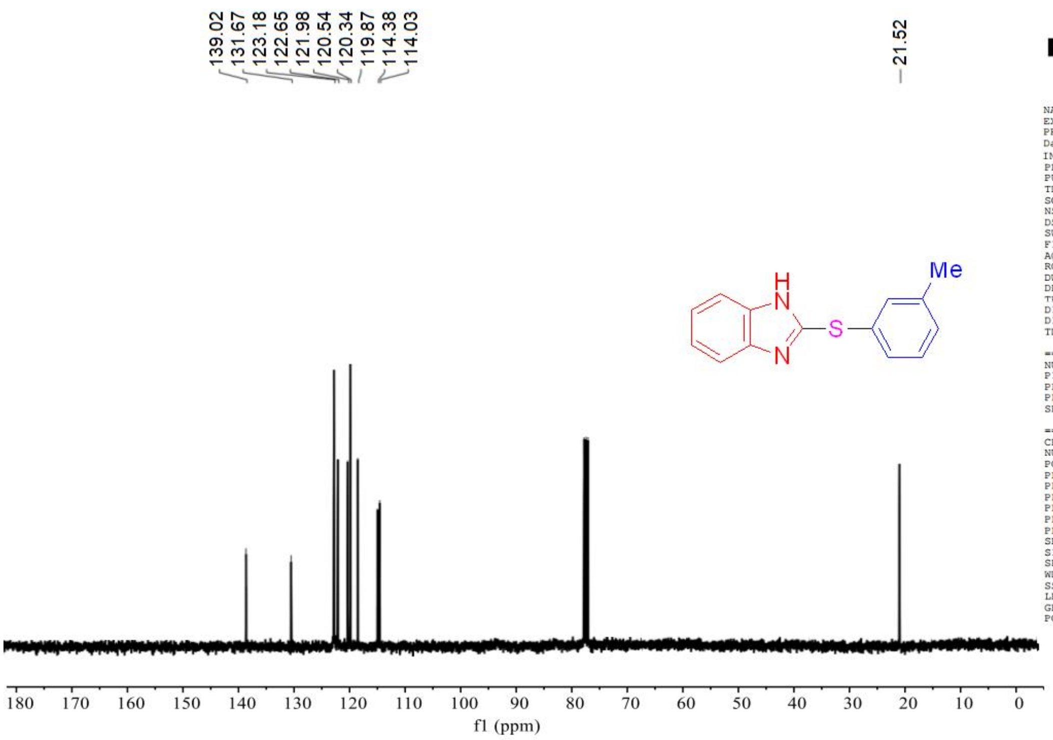

139.02
131.67
123.18
122.65
121.98
120.54
120.34
119.87
114.38
114.03
21.52
H
N
N
S
Me
f1 (ppm)
NAME UN
EXPNO 435
PROCNO 2
Date_ 20240922
INSTRUM spect
PROBHD 5 mm PABBO BB-
PULPROG zgpg
TD 65536
SOLVENT CDCl3
NS 31
DS 0
SWH 25252.525 Hz
FIDRES 0.385323 Hz
AQ 1.2976629 sec
RG 2050
DW 19.800 usec
DE 6.50 usec
TE 293.4 K
D1 3.00000000 sec
D11 0.03000000 sec
TD0 1
======== CHANNEL f1 ========
NUC1 13C
P1 9.00 usec
PL1 -0.90 dB
PL1W 42.02801895 W
SFO1 100.6479784 MHz
======== CHANNEL f2 ========
CPDPRG2 waltz16
NUC2 1H
PCPD2 90.00 usec
PL2 -2.00 dB
PL12 14.16 dB
PL13 17.90 dB
PL2W 11.86359406 W
PL12W 0.28722104 W
PL13W 0.12139934 W
SFO2 400.2216009 MHz
SI 32768
SF 100.6353990 MHz
WDW EM
SSB 0
LB 1.00 Hz
GB 0
PC 1.40

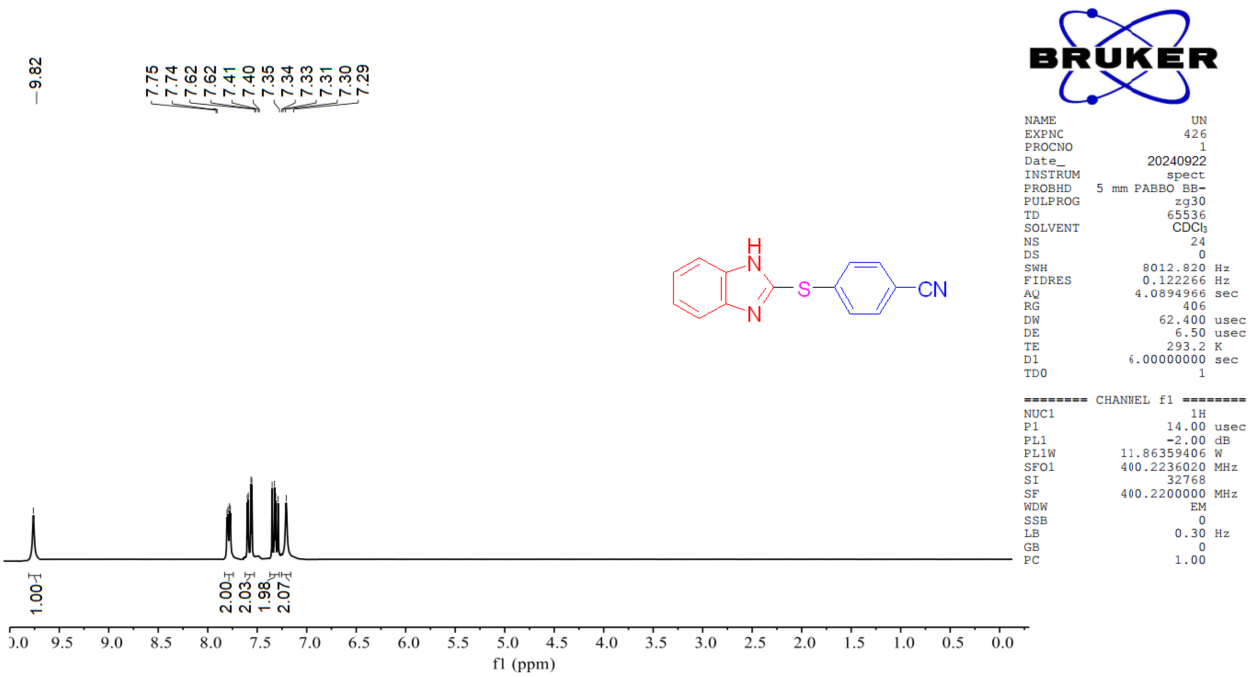

9.82
7.75
7.74
7.62
7.62
7.41
7.40
7.35
7.34
7.33
7.31
7.30
7.29
H
N
N
S
CN
BRUKER
NAME UN
EXPNO 426
PROCNO 1
Date_ 20240922
INSTRUM spect
PROBHD 5 mm PABBO BB-
PULPROG zg30
TD 65536
SOLVENT CDCl3
NS 24
DS 0
SWH 8012.820 Hz
FIDRES 0.122266 Hz
AQ 4.0894966 sec
RG 406
DW 62.400 usec
DE 6.50 usec
TE 293.2 K
D1 6.00000000 sec
TD0 1
======== CHANNEL f1 ========
NUC1 1H
P1 14.00 usec
PL1 -2.00 dB
PL1W 11.86359406 W
SFO1 400.2236020 MHz
SI 32768
SF 400.2200000 MHz
WDW EM
SSB 0
LB 0.30 Hz
GB 0
PC 1.00
1.00
2.00
2.03
1.98
2.07
9.5 9.0 8.5 8.0 7.5 7.0 6.5 6.0 5.5 5.0 4.5 4.0 3.5 3.0 2.5 2.0 1.5 1.0 0.5 0.0
f1 (ppm)

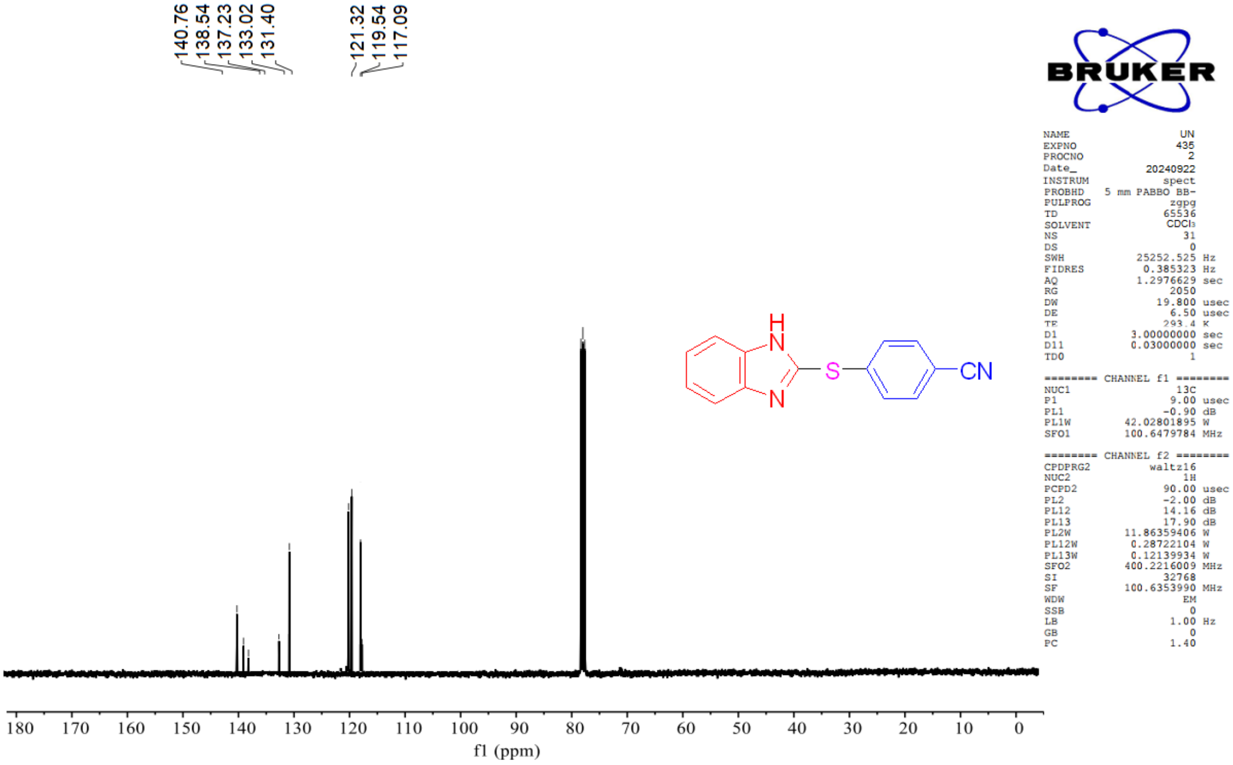

140.76
138.54
137.23
133.02
131.40
121.32
119.54
117.09
BRUKER
NAME UN
EXPNO 435
PROCNO 2
Date_ 20240922
INSTRUM spect
PROBHD 5 mm PABBO BB-
PULPROG zgpg
TD 65536
SOLVENT CDCl3
NS 31
DS 0
SWH 25252.525 Hz
FIDRES 0.385323 Hz
AQ 1.2976629 sec
RG 2050
DW 19.800 usec
DE 6.50 usec
TE 293.4 K
D1 3.00000000 sec
D11 0.03000000 sec
TD0 1
======== CHANNEL f1 ========
NUC1 13C
P1 9.00 usec
PL1 -0.90 dB
PL1W 42.02801895 W
SFO1 100.6479784 MHz
======== CHANNEL f2 ========
CPDPRG2 waltz16
NUC2 1H
PCPD2 90.00 usec
PL2 -2.00 dB
PL12 14.16 dB
PL13 17.90 dB
PL2W 11.86359406 W
PL12W 0.28722104 W
PL13W 0.12139934 W
SFO2 400.2216009 MHz
SI 32768
SF 100.6353990 MHz
WDW EM
SSB 0
LB 1.00 Hz
GB 0
PC 1.40
H
N
N
S
CN
180 170 160 150 140 130 120 110 100 90 80 70 60 50 40 30 20 10 0
f1 (ppm)

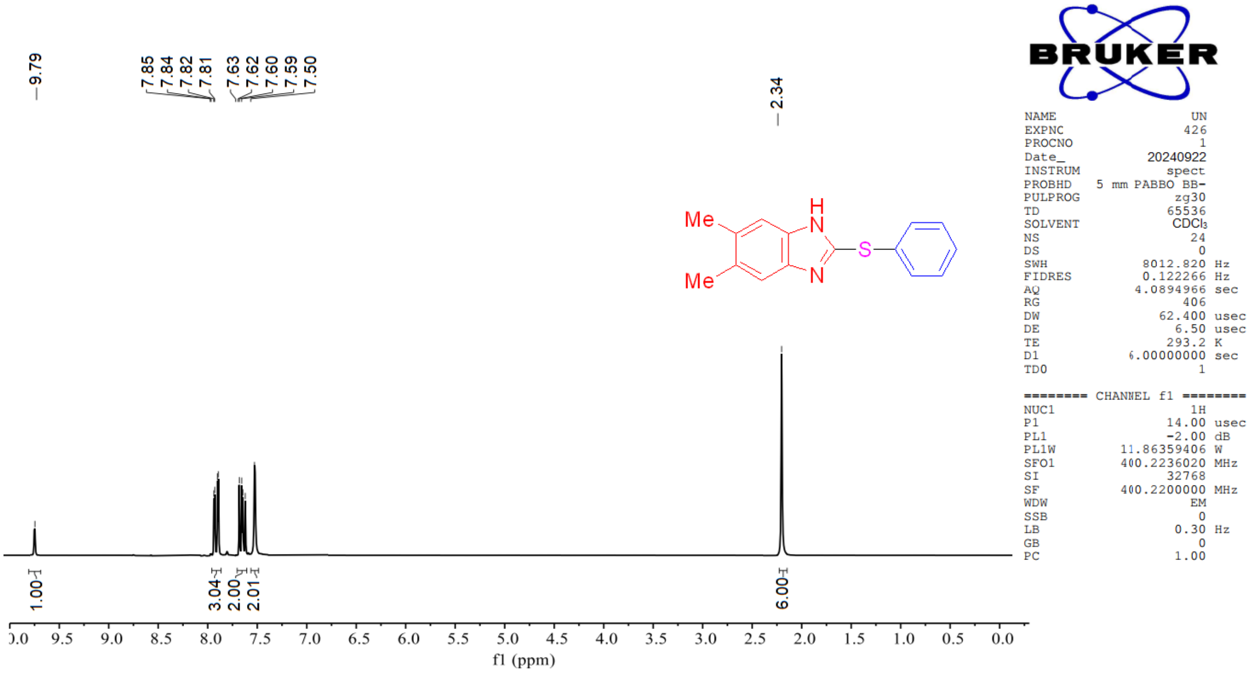
9.79
7.85
7.84
7.82
7.81
7.63
7.62
7.60
7.59
7.50
2.34
Me
Me
H
N
N
S
1.00
3.04
2.00
2.01
6.00
f1 (ppm)
BRUKER
NAME UN
EXPNO 426
PROCNO 1
Date_ 20240922
INSTRUM spect
PROBHD 5 mm PABBO BB-
PULPROG zg30
TD 65536
SOLVENT CDCl3
NS 24
DS 0
SWH 8012.820 Hz
FIDRES 0.122266 Hz
AQ 4.0894966 sec
RG 406
DW 62.400 usec
DE 6.50 usec
TE 293.2 K
D1 6.00000000 sec
TD0 1
======== CHANNEL f1 ========
NUC1 1H
P1 14.00 usec
PL1 -2.00 dB
PL1W 11.86359406 W
SFO1 400.2236020 MHz
SI 32768
SF 400.2200000 MHz
WDW EM
SSB 0
LB 0.30 Hz
GB 0
PC 1.00

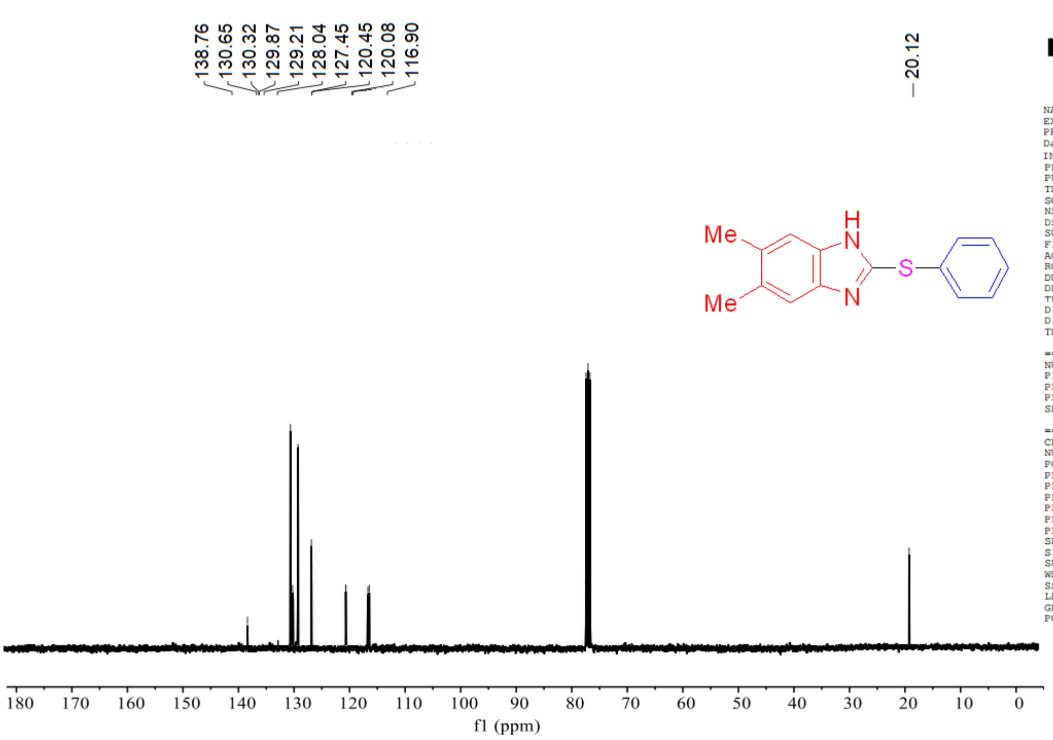
138.76
130.65
130.32
129.87
129.21
128.04
127.45
120.45
120.08
116.90
20.12
Me
Me
H
N
N
S
f1 (ppm)
BRUKER
NAME UN
EXPNO 435
PROCNO 2
Date_ 20240922
INSTRUM spect
PROBHD 5 mm PABBO BB-
PULPROG zgpg
TD 65536
SOLVENT CDCl3
NS 31
DS 0
SWH 25252.525 Hz
FIDRES 0.385323 Hz
AQ 1.2976629 sec
RG 2050
DW 19.800 usec
DE 6.50 usec
TE 293.4 K
D1 3.00000000 sec
D11 0.03000000 sec
TD0 1
======== CHANNEL f1 ========
NUC1 13C
P1 9.00 usec
PL1 -0.90 dB
PL1W 42.02801895 W
SFO1 100.6479784 MHz
======== CHANNEL f2 ========
CPDPRG2 waltz16
NUC2 1H
PCPD2 90.00 usec
PL2 -2.00 dB
PL12 14.16 dB
PL13 17.90 dB
PL2W 11.86359406 W
PL12W 0.28722104 W
PL13W 0.12139934 W
SFO2 400.2216009 MHz
SI 32768
SF 100.6353990 MHz
WDW EM
SSB 0
LB 1.00 Hz
GB 0
PC 1.40

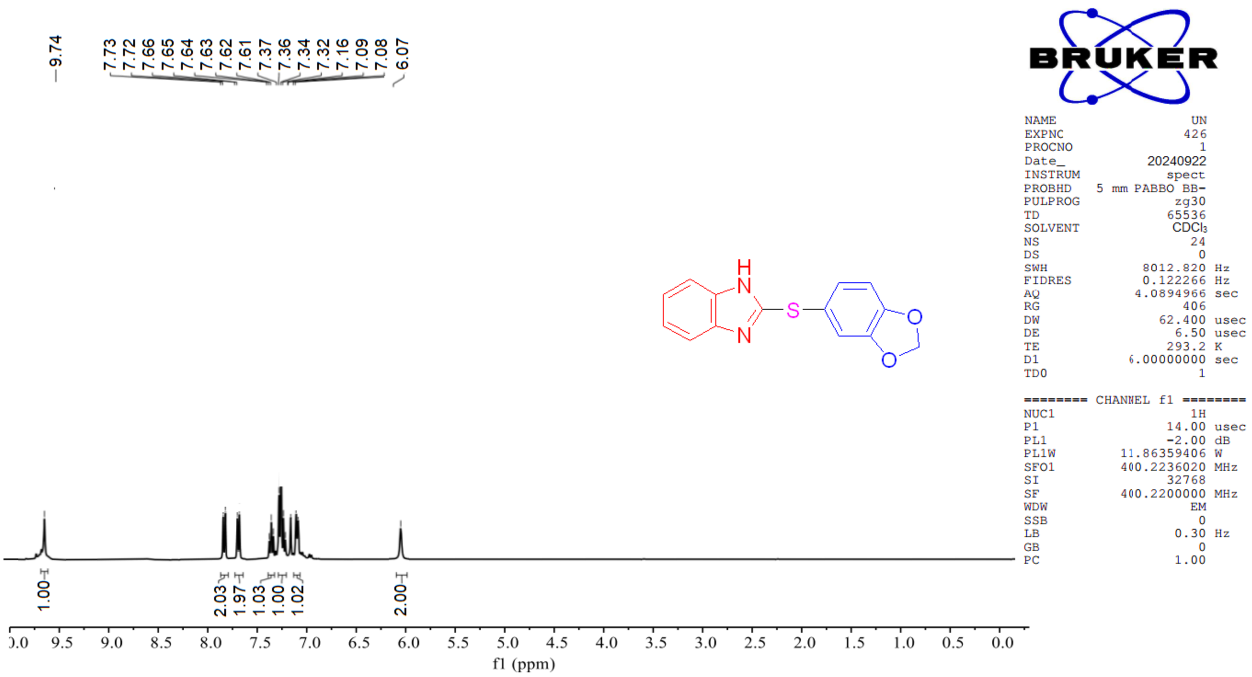
9.74
7.73
7.72
7.66
7.65
7.64
7.63
7.62
7.61
7.37
7.36
7.34
7.32
7.16
7.09
7.08
6.07
1.00
2.03
1.97
1.03
1.00
1.02
2.00
f1 (ppm)
H
N
N
S
O
O
BRUKER
NAME UN
EXPNO 426
PROCNO 1
Date_ 20240922
INSTRUM spect
PROBHD 5 mm PABBO BB-
PULPROG zg30
TD 65536
SOLVENT CDCl3
NS 24
DS 0
SWH 8012.820 Hz
FIDRES 0.122266 Hz
AQ 4.0894966 sec
RG 406
DW 62.400 usec
DE 6.50 usec
TE 293.2 K
D1 6.00000000 sec
TD0 1
======== CHANNEL f1 ========
NUC1 1H
P1 14.00 usec
PL1 -2.00 dB
PL1W 11.86359406 W
SFO1 400.2236020 MHz
SI 32768
SF 400.2200000 MHz
WDW EM
SSB 0
LB 0.30 Hz
GB 0
PC 1.00

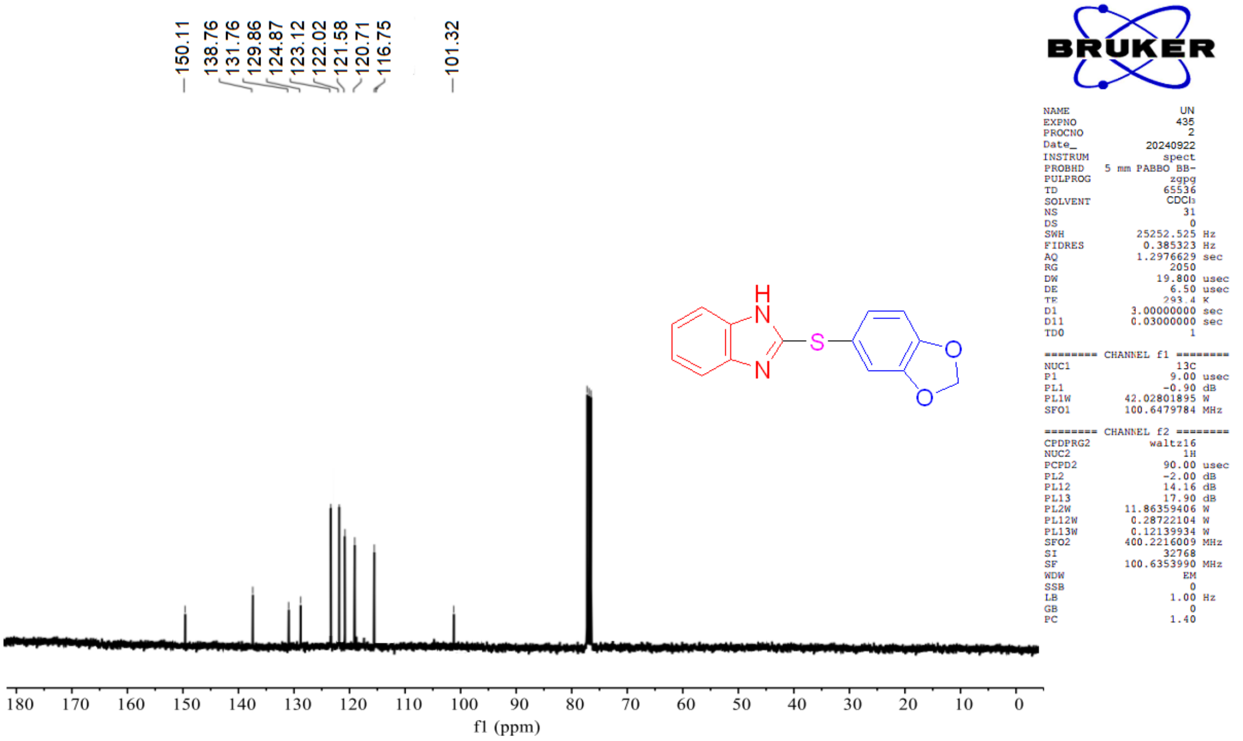
150.11
138.76
131.76
129.86
124.87
123.12
122.02
121.58
120.71
116.75
101.32
f1 (ppm)
H
N
N
S
O
O
BRUKER
NAME UN
EXPNO 435
PROCNO 2
Date_ 20240922
INSTRUM spect
PROBHD 5 mm PABBO BB-
PULPROG zgpg
TD 65536
SOLVENT CDCl3
NS 31
DS 0
SWH 25252.525 Hz
FIDRES 0.385323 Hz
AQ 1.2976629 sec
RG 2050
DW 19.800 usec
DE 6.50 usec
TE 293.4 K
D1 3.00000000 sec
D11 0.03000000 sec
TD0 1
======== CHANNEL f1 ========
NUC1 13C
P1 9.00 usec
PL1 -0.90 dB
PL1W 42.02801895 W
SFO1 100.6479784 MHz
======== CHANNEL f2 ========
CPDPRG2 waltz16
NUC2 1H
PCPD2 90.00 usec
PL2 -2.00 dB
PL12 14.16 dB
PL13 17.90 dB
PL2W 11.86359406 W
PL12W 0.28722104 W
PL13W 0.12139934 W
SFO2 400.2216009 MHz
SI 32768
SF 100.6353990 MHz
WDW EM
SSB 0
LB 1.00 Hz
GB 0
PC 1.40
